# Antigiardial Activity of Novel Guanidine Compounds

**DOI:** 10.1002/cmdc.202200341

**Published:** 2022-09-29

**Authors:** Andrew J. Stevens, Rebecca Abraham, Kelly A. Young, Cecilia C. Russell, Siobhann N. McCluskey, Jennifer R. Baker, Bertha Rusdi, Stephen W. Page, Ryan O'Handley, Mark O'Dea, Sam Abraham, Adam McCluskey

**Affiliations:** ^1^ School of Environmental & Life Sciences The University of Newcastle University Drive Callaghan NSW 2308 Australia; ^2^ Antimicrobial resistance and Infectious Diseases Laboratory, Harry butler Institute Murdoch University 90 South Street Murdoch WA 6150 Australia; ^3^ Neoculi Pty Ltd. Burwood 3125 Vic Australia; ^4^ School of Animal and Veterinary Sciences University of Adelaide, Roseworthy Campus Mudla Wirra Road Roseworthy SA 5371 Australia

## Abstract

From four focused compound libraries based on the known anticoccidial agent robenidine, 44 compounds total were synthesised and screened for antigiardial activity. All active compounds were counter‐screened for antibiotic and cytotoxic action. Of the analogues examined, 21 displayed IC_50_<5 μM, seven with IC_50_<1.0 μM. Most active were 2,2′‐*bis*{[4‐(trifluoromethoxy)phenyl]methylene}carbonimidic dihydrazide hydrochloride (**30**), 2,2′‐*bis*{[4‐(trifluoromethylsulfanyl)phenyl]methylene}carbonimidic dihydrazide hydrochloride (**32**), and 2,2′‐bis[(2‐bromo‐4,5‐dimethoxyphenyl)methylene]carbonimidic dihydrazide hydrochloride (**41**) with IC_50_=0.2 μM. The maximal observed activity was a 5 h IC_50_ value of 0.2 μM for **41**. The clinically used metronidazole was inactive at this timepoint at a concentration of 25 μM. Robenidine off‐target effects at bacteria and cell line toxicity were removed. Analogue **41** was well tolerated in mice treated orally (100 mg/kg). Following 5 h treatment with **41**, no Giardia regrowth was noted after 48 h.

## Graphical abstract

**Rapid and complete killing** of *Giardia* trophozoites: From four focused compound libraries based on the known anticoccidial agent robenidine, 44 compounds were synthesised and screened for antigiardial activity. All active compounds were counter‐screened for antibiotic and cytotoxic action. Of the analogues examined, 21 displayed IC_50_ values less than 5 μM, seven with IC_50_ values less than 1.0 μM.

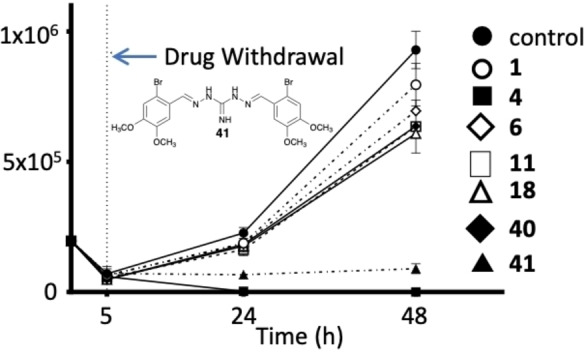

## Introduction

*Giardia duodenalis*, a bi‐nucleate protozoan pathogen, is the most common enteric human parasitic pathogen known, causing up to 1 billion human infections annually.[^1^, ^2^, ^3^, ^4^] Infections are most common in developing nations, but are also prevalent in the developed world. *Giardia* has been added to the World Health Organization neglected diseases initiative.^[5]^ *Giardia* infection occurs via cyst ingestion through a faecal‐oral route or via contaminated food or water.[^5^, ^6^] It results in a mal‐absorptive gastrointestinal disease with acute, chronic and at times re‐occurring symptoms including diarrhoea, bloating and abdominal cramping.^[7]^ Persistent infection, especially in children and immunocompromised hosts, results in long term effects including malnutrition, developmental delay and failure to thrive syndrome.^[8]^

Drug treatment most commonly uses the nitroimidazoles, nitrothiazole, nitrofuran, acridine, benzimidazole, and aminoglycoside compound classes.^[8]^ The most frequently used nitroimidazoles, metronidazole (**1**) and tinidazole (**2**), show an 80–90 % success; while albendazole (**3**), a benzimidazole, has a reported efficacy of 62–95 %. Treatment failures with these drugs are common with side effects including genotoxicity, possible carcinogenesis, nausea, fatigue and general malaise.[^8^, ^9^, ^10^] Disturbingly, resistant organisms have been reported for all the commonly used drugs.[^9^, ^10^, ^11^] This combination of limitations highlights a pressing need for new treatments.

Current antigiardial drug discovery efforts have focused in on the small molecule inhibition of a wide range of protein targets. These span pyruvate‐ferredoxin oxidoreductase, nitroreductase 1 and thioredoxin reductase to activate nitroimidazoles;[^12^, ^13^, ^14^] NADH oxidase for activation of furazolidone;^[15]^ auranofin inhibits thioredoxin reductase;^[15]^ fumagillin inhibits methionine amino‐peptidase;^[16]^ orlistat inhibits gastric lipase;^[17]^ proton‐pump inhibitors have been investigated, with omeprazole inhibiting triosephosphate isomerase, a critical enzyme involved in glucose and glycogen metabolism;[^18^, ^19^] disulfiram inhibits acetaldehyde dehydrogenase;[^20^, ^21^] NBDHEX inhibits glutathione S‐transferase.^[22]^ Other potential targets include antioxidant and metabolic enzymes[^23^, ^24^, ^25^, ^26^, ^27^, ^28^, ^29^] and protein kinases, with the reduced number of core Giardia kinases suggesting limited redundancy, and thus potentially high efficacy.^[30]^

**Figure 1:**
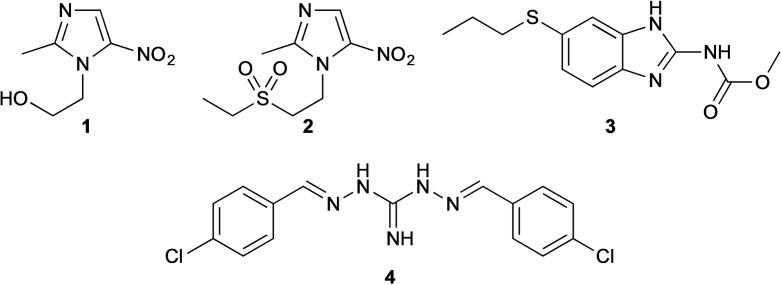
Chemical structures of the clinically used metronidazole (**1**) tinidazole (**2**), albendazole (**3**) and lead robenidine (**4**).

Herein we report on the discovery and development of novel antigiardial agents based on the anticoccicidal agent, robenidine (**4**; Figure 1).[^10^, ^31^]

## Results and Discussion

Preliminary screening of robenidine (**4**) against *Giardia* trophozoites *in vitro* revealed a 5 h IC_50_ of 0.9 μM and a 24 h MIC (minimum inhibitory concentration) of 2.8 μM. Clinically used metronidazole (**1**) was inactive at 25 μM at 5 h, but showed a 24 h IC_50_ of 3.8 μM (Table 1).[^32^, ^33^, ^34^]

**Table 1:** Inhibition of *Giardia duodenalis* by *Library 1* Robenidine analogues possessing mono‐halogenated aromatic rings (**4**–**13**).^[a]^

		Antigiardial activity				
Compound	R	MIC [μM] (24 h)	IC_50_ [μM] (24 h)	Antibacterial activity (Y/N)^[b]^	Toxicity (Y/N) (% growth control)^[c]^	Selectivity ratio^[d]^
Metronidazole	–	8.3	3.8	N	N	>6.6
Robenidine **4**	4‐Cl	2.8	0.9	Y	Y (40.7±22.6)	>27.8
**5**	H	–	–	N	–	–
**6**	4‐Br	8.3	1.7	N	N (97.1±1.6)	>14.7
**7**	4‐F	12.5	6.52	Y	–	–
**8**	3‐Cl	6.25	3.5	Y	–	–
**9**	3‐Br	25	2.9	Y	–	–
**10**	3‐F	>25	>25	Y	–	–
**11**	2‐Br	25	0.8	N	Y (79.1±2.3)	>31.3
**12**	2‐Cl	25	12.37	N	–	–
**13**	2‐F	>25	>25	N	N^[e]^ (93.9±1.9)	–

[a] Toxicity (CaCo‐2 and Vero cells) and antimicrobial assays performed at 25 μM; MIC: minimum inhibitory concentration; ‘–’ not tested, as inhibitory activity at 25 μM<50 %. [b] MSRA and VRE. [c] Percent CaCo‐2 cell growth (25 μM); compounds are indicated as toxic if >20 % cells are affected. [d] Higher ratios indicate a more selective compound. [e] Toxicity assessed with Vero cells, not CaCo‐2 cells.

**Scheme 1:**
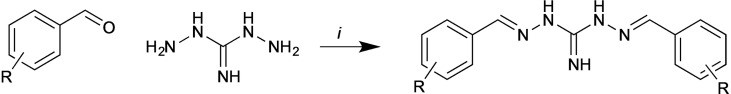
Reagents and conditions: (i) aldehyde (see Table 1 for detail), EtOH, reflux.

While potent, robenidine (**4**) has off target antibacterial activity and cell line toxicity.^[35]^ We sought to address these concerns through the synthesis and subsequent biological screening of focused compound libraries. Robenidine analogues were rapidly accessed through the condensation of a series of benzaldehydes and *N,N*′‐diaminoguanidine hydrochloride (Scheme 1), afforded rapid access to the desired analogues (Table 1). Retention of the *N,N*′‐diaminoguanidine core enabled the SAR activity of the pendent aromatic moieties to be examined in this initial study, as the nature of the robenidine antigiardial activity (what protein target) is unknown.

Limiting *Library 1* to mono‐substituted halogenated benzaldehydes afforded nine analogues (**5**–**13**) in good to excellent yields. Of these, seven returned antigiardia IC_50_ values of <25 μM; three of which displayed no antibacterial activity against methicillin‐resistant *Staphylococcus aureus* (MRSA), vancomycin‐resistant *Enterococcus* (VRE) *or Escherichia coli* (**6**, **11** and **12**); with 4‐Br **6** also showing no cytotoxicity against CaCo‐2 or Vero cells at <10 % cell death at 25 μM. No activity against gram negative bacteria was noted (not shown). In this first library, brominated analogues (**6**, **9** and **11**) displayed the highest levels of Giardia activity, but activity was only marginally enhanced relative to the corresponding Cl‐analogues (**4**, **8** and **12**). Isosteric modification to ‘F’ saw moderate levels of activity (with 2‐F **13**, inactive IC_50_>25 μM).

An extension of our structure activity relationship (SAR) evaluation saw the introduction of alkyl substituents with the synthesis (as per Scheme 1) and evaluation of **14–23**. The screening data for these *Library 2* analogues is presented in Table 2.

**Table 2:** Inhibition *Giardia duodenalis* metabolism by *Library 2* Robenidine analogues possessing alkyl and aromatic substituents (**14**–**23**).^[a]^

		Antigiardial activity				
Compound	R	MIC [μM] (24 h)	IC_50_ [μM] (24 h)	Antibacterial activity (Y/N)^[b]^	Toxicity (Y/N) (% growth control)^[c]^	Selectivity ratio^[d]^
Metronidazole	–	8.3	3.8	N	N	>6.6
**14**	4‐CH_3_	25	6.98‐21.6	Y	–	–
**15**	3‐CH_3_	6.25	4.9	Y	–	–
**16**	2‐CH_3_	25	5.62‐12.98	Y	–	–
**17**	4‐Ph	2.8	0.4	Y	–	–
**18**	2‐Ph	>25	2.8	N	Y (75.4±3.4)	>8.9
**19**	4‐(CH_2_)_2_CH_3_	8.3	0.8	Y	–	–
**20**	4‐(CH_2_)_3_CH_3_	8.3	1.9	Y	–	‐
**21**	4‐CH(CH_3_)_2_	6.25	1.9	Y	–	–
**22**	4‐C(CH_3_)_3_	8.3	3.2	Y	–	–
**23**	4‐CCH	25	1.2	Y	–	–

[a] Toxicity (CaCo‐2 and Vero cells) and antimicrobial assays performed at 25 μM; MIC: minimum inhibitory concentration; ‘–’ not tested, as inhibitory activity at 25 μM<50 %. [b] MSRA and VRE. [c] Percent CaCo‐2 cell growth (25 μM); compounds are indicated as toxic if >20 % cells are affected. [d] Higher ratios indicate a more selective compound.

In each instance the alkyl substituted *Library 2* analogues displayed *Giardia* activity <25 μM. The highest levels of activity were observed with the more hydrophobic substituent with 4‐Ph 17 (IC_50_=0.4 μM) more active than 2‐Ph **18** (IC_50_=2.8 μM), while all other alkyl substituents (excepting the −CH_3_ analogues **14**–**16**) displayed excellent *Giardia* inhibition at 3.2–0.8 μM (**22** and **19** respectively). Acetylenic **23** was well tolerated (IC_50_=1.2 μM). Given the high level of antigiardial activity, each compound was then evaluated for potential antibiotic activity with only 2‐Ph **18** antibiotic inactive, and thus subjected to toxicity assessment with Vero and CaCo‐2 cells. In this instance, **18** displayed toxicity at the 25 μM concentration evaluated. Despite this finding, our data supports a possible dissection of the biological activity profile of this series of compounds, and we note the favourable antigiardial outcome with 2‐disposed analogues (2‐Br **11** and 2‐Ph **18**).

The introduction of a polar or heteroaromatic linked hydrophobic moieties with *Library 3* (**24**–**33**) was largely detrimental to antigiardial activity with only 4‐OCF_3_ **30**, 4‐SCH_3_ **31**, 4‐SCF_3_ **32** and 4‐N(CH_3_)_2_ **33** returning IC_50_ values<2 μM (0.2, 0.9, 0.2 and 1.1 μM, respectively), but with concomitant activity against gram negative bacteria (Table 3). With the observed antibiotic activity, these analogues were not assessed for their toxicity profile.

**Table 3:** Inhibition *Giardia duodenalis* metabolism by *Library 3* Robenidine analogues possessing heteroatom‐substituted aromatic rings (**24**–**33**).^[a]^

		Antigiardial activity				
Compound	R	MLC [μM] (24 h)	IC_50_ [μM] (24 h)	Antibacterial activity (Y/N)^[b]^	Toxicity (Y/N) (% growth control)^[c]^	Selectivity ratio^[d]^
Metronidazole	–	8.3	3.8	N	N	>6.6
**24**	4‐OCH_3_	12.5	5.99	N	Y^[e]^	>4.2
**25**	3‐OCH_3_	>25	>25	Y	–	–
**26**	2‐OCH_3_	>25	>25	Y	–	–
**27**	4‐OH	>25	>25	Y	–	–
**28**	3‐OH	>25	>25	Y	–	–
**29**	2‐OH	>25	>25	Y	–	–
**30**	4‐OCF_3_	2.8	0.2	Y	–	–
**31**	4‐SCH_3_	8.3	0.9	Y	–	–
**32**	4‐SCF_3_	2.8	0.2	Y	–	–
**33**	4‐N(CH_3_)_2_	25	1.1	Y	–	–

[a] Toxicity (CaCo‐2 and Vero cells) and antimicrobial assays performed at 25 μM; MLC: minimum lethal concentration; ‘–’ not tested, as inhibitory activity at 25 μM<50 %. [b] MSRA and VRE. [c] Percent CaCo‐2 cell growth (25 μM); compounds are indicated as toxic if >20 % cells are affected. [d] Higher ratios indicate a more selective compound. [e] Toxicity assessed with Vero cells, not CaCo‐2 cells.

Further library development explored the introduction of additional ring substituents with *Library 4*. The screening outcome for these analogues is presented in Table 4. As speed of action might be anticipated to influence uptake and efficacy of these agents, we explored the time to effective action, noting that essentially 100 % eradication of the Giardia trophozoites was achieved with some analogues in as little as 5 h. As such we determined the efficacy of *Library 4* at a 5 h timepoint.

**Table 4:** Inhibition of *Giardia duodenalis* metabolism by *Library 4* Robenidine analogues possessing di‐, tri‐ and poly‐substituted aromatic rings (**34**–**47**).^[a]^

		Antigiardial activity				
Compound	R	MLC [μM] (24 h)	IC_50_ [μM] (5 h)	Antibacterial activity (Y/N)^[b]^	Toxicity (Y/N) (% growth control)^[c]^	Selectivity ratio^[d]^
34	2,4‐Cl	3.13	2.49	N	Y^[e]^ (55.8±4.3)	>10.0
35	3,5‐Cl	25	11.6	N	–	–
36	2,6‐Cl	–	–	N	–	–
37	2,5‐F	–	–	N	–	–
38	3,4‐F	8.3	1.2	Y	–	–
39	2,3,4,5,6‐F	–	–	N	–	–
40	2‐NHCOCH_3_, 4‐Cl	25	0.8	N	Y (83.1±4.4)	>31.3
41	2‐Br, 4,5‐OCH_3_	2.8	0.2	N	N	>125
42	3‐Br, 4,5‐OCH_3_	3.13	2.7	N	Y^[e]^	>9.3
43	2‐OH, 4‐N(CH_3_)_2_	–	–	Y	–	–
44	3,4‐OCH_3_	>25	24.9	N	–	–
45	3‐NO_2_, 4‐OH	–	–	N	–	–
46	3‐OH, 4‐OCH_3_	–	–	N	–	–
47	3‐OCH_3_, 4‐OH	>25	>25	Y	–	–

[a] Toxicity (CaCo‐2 and Vero cells) and antimicrobial assays performed at 25 μM; MLC: minimum lethal concentration; ‘–’ not tested, as inhibitory activity at 25 μM<50 %. [b] MSRA and VRE. [c] Percent CaCo‐2 cell growth (25 μM); compounds are indicated as toxic if >10 % cells are affected. [d] Higher ratios indicate a more selective compound. [e] Toxicity assessed with Vero cells, not CaCo‐2 cells.

Of the 14 *Library 4* analogues developed, seven (**34**, **35**, **38**, **40**–**42** and **44**) returned antigiardial IC_50_ values of ≤25 μM (Table 4 and ESI†). The introduction of a second −Cl moiety (c.f. **34** vs **4**) was detrimental to activity, but the 3,4‐difluoro **38** was active (IC_50_=1.2 μM). However, the equivalent 2,5‐difluoro **37** and pentafluoro **39** were inactive. Other variations in the phenyl ring substituent level and pattern were largely detrimental to activity (ESI†). Of *Library 4*, only **41** was devoid of antibacterial activity and cell toxicity, and fortuitously this was the most active analogue with a 5 h IC_50_ of 0.2 μM.

This combined with the lack of antibacterial activity and cell toxicity differentiates **41** from **4** as an antigiardial development lead. To further investigate the validity of this compound series as leads in the development of potential antigiardial treatments, disubstituted **41** was examined in a mouse model of *Giardia* infection.

**Figure 2:**
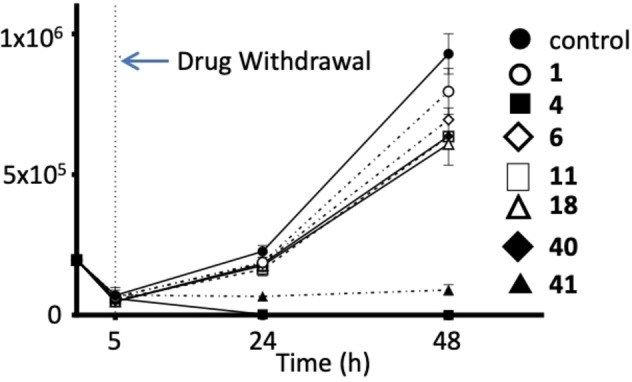
Growth recovery of *Giardia duodenalis* after exposure to selected analogues for 5 h. ‐λ‐ control (no compound); ‐○‐ metronidazole (1); ‐ν‐ Robenidine (**4**); ‐υ‐(**40**); ‐◊‐(**6**); ‐▵‐ (**18**); ‐ (**11**); ‐←‐ (**41**) Cell numbers were determined 24 and 48 h post compound removal.

Our preliminary animal studies revealed no adverse effects with mice treated *per os* with 100 mg/kg **41** per day for 3 days, strongly suggesting that **41** is a viable lead development candidate. Moreover, in washout experiments, the removal of **4** and **41** after *Giardia* death did not result in *Giardia* regrowth after 48 hour (Figure 2).

## Conclusions

From the analogues noted herein, **6**, **11**, **13**, **40** and **41** were identified as potential antigiardial development candidates. Analogue **41** displays the most promising activity and safety profile of the analogues developed herein. All analogues showed the expected high levels of antigiardial activity.

The initial limitations of **4** as an antigiardial agent have been overcome through the development of **41**, with a 5‐fold potency increase (5 h IC_50_=0.2 μM), no antibacterial activity and no observed cytotoxicity in the systems examined. This activity also contrasts the clinically used metronidazole with known antibacterial activity, slow onset of activity (24 h vs 5 h) and 20‐fold lower antigiardial activity. Indeed, **41** shows marked impact on Giardia trophozoites after only 1–2 h (data not shown).

As yet the drug target of these analogues remains undetermined. However, in our antibiotic studies with related compounds we observed that robenidine analogues affected the bacterial cell wall.[^31^, ^32^, ^36^, ^37^, ^38^, ^39^] It is possible that a similar mechanism, against the trophozoite cell wall is in play. This would be in keeping with our electron microscopy observations of gross morphological changes to the dorsal cytoplasmic membrane of trophozoites, including membrane rupture. These effects would adversely affect the ability of the Giardia trophozoite to attach to membrane, interrupting a crucial stage in the parasite lifecycle, with the observed parasite killing.[^31^, ^36^]

## Conflict of interest

S.W.P. is director of Neoculi Pty. Ltd., who are seeking to develop these analogues for use in at‐risk species. The authors declare no other conflicts of interest.

1

## Supporting information

As a service to our authors and readers, this journal provides supporting information supplied by the authors. Such materials are peer reviewed and may be re‐organized for online delivery, but are not copy‐edited or typeset. Technical support issues arising from supporting information (other than missing files) should be addressed to the authors.

Supporting Information

## Data Availability

The data that support the findings of this study are available in the supplementary material of this article.

## References

[1] M. C. Halliez , A. G. Buret , World J. Gastroenterol. 2013, 19, 8974–8985. 24379622 10.3748/wjg.v19.i47.8974 PMC3870550

[2] J. Upcroft , P. Upcroft , BioEssays 1998, 20, 256–263. 9631653 10.1002/(SICI)1521-1878(199803)20:3<256::AID-BIES9>3.0.CO;2-P

[3] P. Upcroft , J. A. Upcroft , Clin. Microbiol. Rev. 2001, 14, 150–164. 11148007 10.1128/CMR.14.1.150-164.2001 PMC88967

[4] M. D. Kirk , S. M. Pires , R. E. Black , M. Caipo , J. A. Crump , B. Devleesschauwer , D. Döpfer , A. Fazil , C. L. Fischer-Walker , T. Hald , A. J. Hall , K. H. Keddy , R. J. Lake , C. F. Lanata , P. R. Torgerson , A. H. Havelaar , F. J. Angulo , PLoS Med. 2015, 12, e1001921. 26633831 10.1371/journal.pmed.1001921 PMC4668831

[5] L. Savioli , H. Smith , A. Thompson , Trends Parasitol. 2006, 22, 203–208. 16545611 10.1016/j.pt.2006.02.015

[6] R. C. A. Thompson , Int. J. Parasitol. 2000, 30, 1259–1267. 11113253 10.1016/s0020-7519(00)00127-2

[7] A. G. Buret , Parasite Paris France 2008, 15, 261–265. 18814692 10.1051/parasite/2008153261

[8] J. M. Wright , L. A. Dunn , P. Upcroft , J. A. Upcroft , Expert Opin. Drug Saf. 2003, 2, 529–541. 14585063 10.1517/14740338.2.6.529

[9] A. Bendesky , D. Menéndez , P. Ostrosky-Wegman , Mutat. Res. 2002, 511, 133–144. 12052431 10.1016/s1383-5742(02)00007-8

[10] L. Jokipii , A. M. Jokipii , J. Infect. Dis. 1979, 140, 984–988. 541526 10.1093/infdis/140.6.984

[11] R. Argüello-García , D. Leitsch , T. Skinner-Adams , M. G. Ortega-Pierres , Adv. Parasitol. 2020, 107, 201–282. 32122530 10.1016/bs.apar.2019.11.003

[12] B. R. E. Ansell , M. J. McConville , S. Y. Ma'ayeh , M. J. Dagley , R. B. Gasser , S. G. Svärd , A. R. Jex , Biotechnol. Adv. 2015, 33, 888–901. 25922317 10.1016/j.biotechadv.2015.04.009

[13] P. S. Hoffman , G. Sisson , M. A. Croxen , K. Welch , W. D. Harman , N. Cremades , M. G. Morash , Antimicrob. Agents Chemother. 2006, 51, 868–876. 17158936 10.1128/AAC.01159-06 PMC1803158

[14] J. Müller , J. Wastling , S. Sanderson , N. Müller , A. Hemphill , Antimicrob. Agents Chemother. 2007, 51, 1979–1986. 17438059 10.1128/AAC.01548-06 PMC1891416

[15] N. Tejman-Yarden , Y. Miyamoto , D. Leitsch , J. Santini , A. Debnath , J. Gut , J. H. McKerrow , S. L. Reed , L. Eckmann , Antimicrob. Agents Chemother. 2013, 57, 2029–2035. 23403423 10.1128/AAC.01675-12 PMC3632933

[16] L. Kulakova , A. Galkin , C. Z. Chen , N. Southall , J. J. Marugan , W. Zheng , O. Herzberg , Antimicrob. Agents Chemother. 2014, 58, 7303–7311. 25267663 10.1128/AAC.03834-14 PMC4249522

[17] J. Hahn , F. Seeber , H. Kolodziej , R. Ignatius , M. Laue , T. Aebischer , C. Klotz , PLoS One 2013, 8, e71597. 23977083 10.1371/journal.pone.0071597 PMC3747212

[18] H. Reyes-Vivas , I. de la M la Mora , A. Castillo-Villanueva , L. Yépez-Mulia , G. Hernández-Alcántara , R. Figueroa-Salazar , I. García-Torres , S. Gómez-Manzo , S. T. Méndez , A. Vanoye-Carlo , J. Marcial-Quino , A. Torres-Arroyo , J. Oria-Hernández , P. Gutiérrez-Castrellón , S. Enríquez-Flores , G. López-Velázquez , Antimicrob. Agents Chemother. 2014, 58, 7072–7082. 25223993 10.1128/AAC.02900-14 PMC4249547

[19] I. García-Torres , I. de la M la Mora , J. Marcial-Quino , S. Gómez-Manzo , A. Vanoye-Carlo , G. Navarrete-Vázquez , B. Colín-Lozano , P. Gutiérrez-Castrellón , E. Sierra-Palacios , G. López-Velázquez , S. Enríquez-Flores , Biochim. Biophys. Acta Gen. Subj. 2016, 1860, 97–107. 10.1016/j.bbagen.2015.10.021 26518348

[20] T. Nash , W. G. Rice , Antimicrob. Agents Chemother. 1998, 42, 1488–1492. 9624499 10.1128/aac.42.6.1488 PMC105627

[21] A. Galkin , L. Kulakova , K. Lim , C. Z. Chen , W. Zheng , I. V. Turko , O. Herzberg , J. Biol. Chem. 2014, 289, 10502–10509. 24558036 10.1074/jbc.M114.553123 PMC4036171

[22] M. Lalle , S. Camerini , S. Cecchetti , R. Finelli , G. Sferra , J. Müller , G. Ricci , E. Pozio , Front. Microbiol. 2015, 06, 544. 10.3389/fmicb.2015.00544 PMC4450592 26082764

[23] Z. Li , L. Kulakova , L. Li , A. Galkin , Z. Zhao , T. E. Nash , P. S. Mariano , O. Herzberg , D. Dunaway-Mariano , Bioorg. Chem. 2009, 37, 149–161. 19640561 10.1016/j.bioorg.2009.06.001 PMC4590290

[24] Z. Li , Z. Liu , D. W. Cho , J. Zou , M. Gong , R. M. Breece , A. Galkin , L. Li , H. Zhao , G. D. Maestas , D. L. Tierney , O. Herzberg , D. Dunaway-Mariano , P. S. Mariano , J. Inorg. Biochem. 2011, 105, 509–517. 21333622 10.1016/j.jinorgbio.2010.12.012 PMC3071891

[25] F. Guo , G. Ortega-Pierres , R. Argüello-García , H. Zhang , G. Zhu , Front. Microbiol. 2015, 6, 753. 26257723 10.3389/fmicb.2015.00753 PMC4510421

[26] D. Leitsch , C. F. Williams , I. Hrdý , Trends Parasitol. 2018, 34, 576–589. 29807758 10.1016/j.pt.2018.04.007

[27] A. Debnath , M. Ndao , S. L. Reed , Gut Microbes 2013, 4, 66–71. 23137963 10.4161/gmic.22596 PMC3555889

[28] E. L. Jarroll , K. Şener , Drug Resist. Updates 2003, 6, 239–246. 10.1016/s1368-7646(03)00065-7 14643294

[29] Y. Miyamoto , L. Eckmann , Front. Microbiol. 2015, 6, 1208. 26635732 10.3389/fmicb.2015.01208 PMC4652082

[30] K. M. Hennessey , T. R. Smith , J. W. Xu , G. C. M. Alas , K. K. Ojo , E. A. Merritt , A. R. Paredez , PLoS Neglected Trop. Dis. 2016, 10, e0005107. 10.1371/journal.pntd.0005107 PMC5091913 27806042

[31] R. J. Abraham , S. Abraham , A. J. Stevens , S. W. Page , A. McCluskey , D. J. Trott , R. M. O'Handley , Int. J. Parasitol. Drugs Drug Resist. 2019, 10, 38–44. 31015151 10.1016/j.ijpddr.2019.04.003 PMC6479099

[32] R. J. Abraham , A. J. Stevens , K. A. Young , C. Russell , A. Qvist , M. Khazandi , H. S. Wong , S. Abraham , A. D. Ogunniyi , S. W. Page , R. O'Handley , A. McCluskey , D. J. Trott , J. Med. Chem. 2016, 59, 2126–2138. 26765953 10.1021/acs.jmedchem.5b01797

[33] E. Bénéré , R. A. I. da Luz , M. Vermeersch , P. Cos , L. Maes , J. Microbiol. Methods 2007, 71, 101–106. 17888535 10.1016/j.mimet.2007.07.014

[34] C. G. Clark , L. S. Diamond , Clin. Microbiol. Rev. 2002, 15, 329–341. 12097242 10.1128/CMR.15.3.329-341.2002 PMC118080

[35] B. Nguyen , M. P. H. Lee , D. Hamelberg , A. Joubert , C. Bailly , R. Brun , S. Neidle , W. D. Wilson , J. Am. Chem. Soc. 2002, 124, 13680–13681. 12431090 10.1021/ja027953c

[36] R. J. Abraham , M. O'Dea , B. Rusdi , S. W. Page , R. O'Handley , S. Abraham , J. Microbiol. Methods 2018, 145, 7–9. 29198594 10.1016/j.mimet.2017.11.025

[37] H. T. Nguyen , L. A. O'Donovan , H. Venter , C. C. Russell , A. McCluskey , S. W. Page , D. J. Trott , A. D. Ogunniyi , Antibiotics 2021, 10, 307. 33802844 10.3390/antibiotics10030307 PMC8002630

[38] A. D. Ogunniyi , M. Khazandi , A. J. Stevens , S. K. Sims , S. W. Page , S. Garg , H. Venter , A. Powell , K. White , K. R. Petrovski , G. Laven-Law , E. G. Tótoli , H. R. Salgado , H. Pi , G. W. Coombs , D. L. Shinabarger , J. D. Turnidge , J. C. Paton , A. McCluskey , D. J. Trott , PLoS One 2017, 12, e0183457. 28873428 10.1371/journal.pone.0183457 PMC5584945

[39] H. Pi , H. T. Nguyen , H. Venter , A. R. Boileau , L. Woolford , S. Garg , S. W. Page , C. C. Russell , J. R. Baker , A. McCluskey , L. A. O'Donovan , D. J. Trott , A. D. Ogunniyi , Front. Microbiol. 2020, 11, 1556. 32849325 10.3389/fmicb.2020.01556 PMC7417630

