# Supplementary material for: Antigiardial Activity of Novel Guanidine Compounds
Source: ChemMedChem. 2022 Sep 29;17(21):e202200341. doi: 10.1002/cmdc.202200341 (PMC9828538; doi:10.1002/cmdc.202200341)
Supplement: Supplementary file 1 — Supporting Information [file CMDC-17-0-s001.pdf]

ChemMedChem

Supporting Information

Antigiardial Activity of Novel Guanidine Compounds

Andrew J. Stevens[+], Rebecca Abraham[+], Kelly A. Young, Cecilia C. Russell[+], Siobhann N. McCluskey, Jennifer R. Baker, Bertha Rusdi, Stephen W. Page, Ryan O'Handley, Mark O'Dea, Sam Abraham,* and Adam McCluskey*

SI experimental

Chemistry - General Methods

All reagents were purchased from Sigma-Aldrich, AK Scientific, Matrix Scientific or Alfa Aesar and were used without purification. All solvents were re-distilled from glass prior to use. Syntheses using microwave irradiation were performed using a Biotage® Initiator+ system.

^{1}H and ^{13}C NMR spectra were recorded on a Bruker Advance™ AMX 400 at 400.13 and 100.62 MHz, respectively and Advance™ AMX 300 at 300.1 and 75.4 MHz, respectively. Chemical shifts (δ) are reported in parts per million (ppm) measured relative to the internal standards. Coupling constants (*J*) are expressed in hertz (Hz). Mass spectra were recorded on a Shimadzu LCMS 2010 EV or Agilent 6100 series single quadrupole LCMS using a mobile phase of 1:1 acetonitrile : H_2O with 0.1% formic acid. The University of Wollongong, Australia, Mass Spectrometry User resource & Research Facility (MSURRF) analysed samples for High Resolution Mass Spectrometry HRMS. Analytical HPLC traces were obtained using a Shimadzu system possessing a SIL-20A auto-sampler, dual LC-20AP pumps, CBM-20A bus module, CTO-20A column heater, and a SPD-20A UV/vis detector. This system was fitted with an Alltima™ C_{18} 5 µm 150 mm × 4.6 mm column with solvent A: 0.06% trifluoroacetic acid (TFA) in water and solvent B: 0.06% TFA in $CH_3CN–H_2O$ (90 : 10). In each case HPLC traces were acquired at a flow rate of 2.0 mL min^{-1}, gradient 10–100 (%B), over 15.0 min, with detection at 220 nm and 254 nm. All samples returned satisfactory analyses. Compound purity was confirmed by a combination of LC-MS (HPLC), micro and/or high resolution mass spectrometry and NMR analysis. All analogues are ≥ 95% purity.

Melting points were recorded on a Büchi Melting Point M-565 instrument. IR spectra were recorded on a PerkinElmer Spectrum Two™ FTIR Spectrometer with the UATR accessories. Thin layer chromatography (TLC) was performed on Merck 60 F254 pre-coated aluminium plates with a thickness of 0.2 mm. Column chromatography was performed under 'flash' conditions on Merck silica gel 60 (230–400 mesh).

General Method A. A suspension of the appropriate aldehyde (2.2 equiv.) and N,N′-diaminoguanidine hydrochloride (1.0 equiv.) in EtOH was heated at reflux for 16 h. The mixture was cooled and diluted with Et_2O to effect crystallization. The resulting precipitate was collected and washed with Et_2O to afford the carbonimidic dihydrazide.

General Method B. A suspension of aldehyde (2.2 equiv.) and N,N′-diaminoguanidine hydrochloride (1.0 equiv.) in EtOH (5 mL) was subjected to microwave irradiation (150 W) at 120 °C for 10 min. Most of the solvent was then removed *in vacuo*, Et_2O (5 mL) was added, and the flask was chilled to effect crystallization. The resulting precipitate was collected and washed with Et_2O (5 mL) to afford the carbonimidic dihydrazide.

*2,2'-Bis[(4-chlorophenyl)methylene]carbonimidic dihydrazide hydrochloride (**4**)*

Synthesized according to General Method A from 4-chlorobenzaldehyde to afford the title compound as a white solid (91%). MP: 258°C (decomp); ^{1}H NMR (400 MHz, DMSO-d_6) δ 12.33 (br. s, 2H), 8.60 (s, 2H), 8.42 (s, 2H), 7.98 (d, *J* = 8.4 Hz, 4H), 7.56 (d, *J* = 8.4 Hz, 4H); ^{13}C NMR (101 MHz, DMSO-d_6) δ 152.9, 147.6 (2 × C), 135.3 (2 × C), 132.3 (2 × C), 129.6 (4 × C), 128.8 (4 × C); LRMS (ESI) *m/z* (%): 332 (100) [*M*-HCl-H]$^-$.

*2,2'-Bis(phenylmethylene)carbonimidic dihydrazide hydrochloride (**5**)*

Synthesized according to General Method B from benzaldehyde to afford the title compound as a white solid (88%). MP: 234–237°C. ^{1}H NMR (300 MHz, DMSO-d_6) δ 12.39 (br. s, 2H), 8.55 (s, 2H), 8.46 (s, 2H), 8.01 – 7.88 (m, 4H), 7.55 – 7.41 (m, 6H); ^{13}C NMR (75 MHz, DMSO-d_6) δ 152.9, 148.9 (2 × C), 133.3 (2 × C), 130.8 (2 × C), 128.8 (4 × C), 127.9 (4 × C); HRMS: *m/z* (calcd for $C_{15}H_{15}N_5$, 265.1327; found 265.1246).

*2,2'-Bis[(4-bromophenyl)methylene]carbonimidic dihydrazide hydrochloride (**6**)*

Synthesized according to General Method B from 4-bromobenzaldehyde to afford the title compound as a white solid (89%). MP: 224–226 °C. ^{1}H NMR (400 MHz DMSO-d_6) δ 12.42 (br. s, 2H), 8.60 (s, 2H), 8.42 (s, 2H), 7.91 (d, J = 8.5 Hz, 4H), 7.69 (d, J = 8.5 Hz, 4H); ^{13}C NMR (101 MHz DMSO-d_6) δ 152.9, 147.7 (2 × C), 132.6 (2 × C), 131.7 (4 × C), 129.7 (4 × C), 124.2 (2 × C); HRMS: m/z calcd for $C_{15}H_{13}Br_2N_5$, 422.9517; found, 422.9320.

*2,2'-Bis[(4-fluorophenyl)methylene]carbonimidic dihydrazide hydrochloride (**7**)*

Synthesized by according to General Method A from 4-fluorobenzaldehyde to afford the title compound as a white solid (69%). MP: 258–260°C. ^{1}H NMR (400 MHz, DMSO-d_6) δ 12.23 (s, 1H), 8.54 (br. s, 1H), 8.42 (br. s, 1H), 8.02 (dd, J =8.4, 5.8 Hz, 2H), 7.34 (t, J = 8.8 Hz, 2H); ^{13}C NMR (101 MHz, DMSO-d_6) δ 163.6 (d, $^{1}J_{CF}$ = 249.5 Hz, 2 × C), 152.9, 147.7 (2 × C), 130.2 (d, $^{3}J_{CF}$ = 8.7 Hz, 4 × C), 129.9 (d, $^{4}J_{CF}$ = 2.9 Hz, 2 × C), 115.8 (d, $^{2}J_{CF}$ = 22.0 Hz, 4 × C); LRMS: (ESI+) m/z: 302 ($C_{15}H_{14}F_2N_5$) [M-Cl]$^{+}$; (ESI-) m/z: 300 ($C_{15}H_{12}F_2N_5$) [M-HCl-H]$^{-}$.

*2,2'-Bis[(3-chlorophenyl)methylene]carbonimidic dihydrazide hydrochloride (**8**)*

Synthesized according to General Method A from 3-chlorobenzaldehyde to afford the title compound as a white solid (82%). MP:252–254°C. ^{1}H NMR (400 MHz, DMSO-d_6) δ 12.38 (br. s, 2H), 8.66 (s, 2H), 8.42 (s, 2H), 8.16 (s, 2H), 7.83 – 7.82 (m, 2H), 7.55 – 7.49 (m, 4H); ^{13}C DEPTQ-135 NMR (101 MHz, DMSO-d_6) δ 153.0, 147.4 (2 × C), 135.5 (2 × C), 133.8 (2 × C), 130.6 (2 × C), 130.4 (2 × C), 127.2 (2 × C), 126.6 (2 × C); LCMS (ESI) m/z: 334 [M-Cl]$^{+}$, 332 [M-HCl-H]$^{-}$.

*2,2'-Bis[(3-bromophenyl)methylene]carbonimidic dihydrazide hydrochloride (**9**)*

Synthesized according to General Method B from 3-bromobenzaldehyde to afford the title compound as a white solid (44%). MP: 248–251°C. ^{1}H NMR (400 MHz, DMSO-d_6) δ 12.45 (br. s, 2H), 8.68 (s, 2H), 8.41 (s, 2H), 8.29 (s, 2H), 7.87 (d, J = 7.9 Hz, 2H), 7.70 – 7.63 (m, 2H), 7.44 (t, J = 7.9 Hz, 2H); ^{13}C NMR (101 MHz, DMSO-d_6) δ 153.0, 147.4 (2 × C), 135.7 (2 × C), 133.3 (2 × C), 130.9 (2 × C), 129.5 (2 × C), 127.6 (2 × C), 122.3 (2 × C); HRMS: m/z (calcd for $C_{15}H_{13}Br_2N_5$, 422.9517; found 422.9413).

*2,2'-Bis[(3-fluorophenyl)methylene]carbonimidic dihydrazide hydrochloride (**10**)*

Synthesized according to General Method A from 3-fluorobenzaldehyde to afford the title compound as a white solid (56%). MP: 260–263°C· ^{1}H NMR (400 MHz, DMSO-d_6) δ 12.41 (br. s, 2H), 8.65 (s, 2H), 8.44 (s, 2H), 7.98 – 7.96 (m, 2H), 7.69 (d, J = 7.7 Hz, 2H), 7.53 (td, J = 8.0, 6.1 Hz, 2H), 7.32 (td, J = 8.4, 2.1 Hz, 2H); ^{13}C DEPTQ-135 NMR (101 MHz, DMSO-d_6) δ 162.5 (d, $^{1}J_{CF}$ = 243.5 Hz, 2 × C), 153.0, 147.6 (br., 2 × C), 135.8 (d, $^{3}J_{CF}$ = 8.3 Hz, 2 × C), 130.9 (d, $^{3}J_{CF}$ = 8.3 Hz, 2 × C), 124.9 (d, $^{4}J_{CF}$ = 2.4 Hz, 2 × C), 117.6 (d, $^{2}J_{CF}$ = 21.5 Hz, 2 × C), 113.3 (d, $^{2}J_{CF}$ = 22.8 Hz, 2 × C); LRMS: (ESI+) m/z: 302 ($C_{15}H_{14}F_2N_5$) [M -Cl]$^{+}$; (ESI-) m/z: 300 ($C_{15}H_{12}F_2N_5$) [M-HCl-H]$^{-}$.

*2,2'-Bis[(2-bromophenyl)methylene]carbonimidic dihydrazide hydrochloride (**11**)*

Synthesized according to General Method B from 2-bromobenzaldehyde to afford the title compound as a white solid (72%). MP: 267–270 °C. ^{1}H NMR (400 MHz DMSO-d_6) δ 8.84 (s, 2H), 8.69 (s, 2H), 8.41 (dd, J = 7.8, 1.7 Hz, 2H), 7.71 – 7.69 (m, 2H), 7.50 – 7.47 (m, 2H), 7.44−7.37 (m, 2H); ^{13}C NMR (150 MHz DMSO-d_6) δ 152.8, 147.3 (2 × C), 133.1 (2 × C), 132.4 (2 × C), 132.1 (2 × C), 128.5 (2 × C), 127.9 (2 × C), 123.9 (2 × C); HRMS: m/z calcd for $C_{15}H_{13}Br_2N_5$, 422.9517; found, 422.9355.

*2,2'-Bis[(2-chlorophenyl)methylene]carbonimidic dihydrazide hydrochloride (**12**)*

Synthesized according to General Method A from 2-chlorobenzaldehyde to afford the title compound as a white solid (51%). MP: 267–270°C. ^{1}H NMR (400 MHz, DMSO-d_6) δ 12.60 (br. s, 2H), 8.87 (s, 2H), 8.67 (s, 2H), 8.42 (dd, J = 7.4, 1.8 Hz, 2H), 7.58 – 7.56 (m, 2H), 7.51 - 7.47 (m, 4H); ^{13}C NMR (101 MHz, DMSO-d_6) δ 152.8, 145.0 (2 × C), 133.6 (2 × C), 132.3 (2 × C),

130.6 (2 × C), 129.9 (2 × C), 128.0 (2 × C), 127.5 (2 × C); LRMS: (ESI+) m/z: 334 ($C_{15}H_{14}Cl_2N_5$) [*M* -Cl]$^+$; (ESI$^-$) m/z: 332 ($C_{15}H_{12}Cl_2N_5$) [*M*-HCl-H]$^-$.

*2,2'-Bis[(2-fluorophenyl)methylene]carbonimidic dihydrazide hydrochloride (**13**)*

Synthesized according to General Method A from 2-fluorobenzaldehyde to afford the title compound as a white solid (54%). MP: 267−269°C. ^{1}H NMR (400 MHz, DMSO-d_6) δ 12.44 (br. s, 2H), 8.70 (br. s, 2H), 8.62 (br. s, 2H), 8.35 (t, *J* = 7.2 Hz, 2H), 7.58 – 7.53 (m, 2H), 7.34 – 7.31 (m, 4H); ^{13}C DEPTQ-135 NMR (101 MHz, DMSO-d_6) δ 161.0 (d, $^1J_{CF}$ = 251.0 Hz, 2 × C), 152.8, 141.7 (br., 2 × C), 132.9 (d, $^3J_{CF}$ = 8.6 Hz, 2 × C), 127.3 (2 × C), 124.8 (d, $^4J_{CF}$ = 3.1 Hz, 2 × C), 120.9 (d, $^3J_{CF}$ = 9.6 Hz, 2 × C), 116.0 (d, $^2J_{CF}$ = 20.7 Hz, 2 × C); LRMS: (ESI+) m/z: 302 ($C_{15}H_{14}F_2N_5$) [*M* -Cl]$^+$; (ESI$^-$) m/z: 300 ($C_{15}H_{12}F_2N_5$) [*M*-HCl-H]$^-$.

*2,2'-Bis[(4-methylphenyl)methylene]carbonimidic dihydrazide hydrochloride (**14**)*

Synthesized according to General Method A from 4-methylbenzaldehyde to afford the title compound as a white solid (90%). MP: 229–232°C. ^{1}H NMR (400 MHz, DMSO-d_6) δ 12.18 (br. s, 2H), 8.44 (s, 2H), 8.39 (s, 2H), 7.82 (d, *J* = 8.0 Hz, 4H), 7.30 (d, *J* = 8.0 Hz, 4H), 2.36 (s, 6H); ^{13}C NMR (101 MHz, DMSO-d_6) δ 152.7, 148.9 (2 x C), 140.8 (2 x C), 130.7 (2 x C), 129.4 (4 x C), 127.9 (4 x C), 21.2 (2 x C); LRMS (ESI) *m/z*: 294 [*M*-Cl]$^+$.

*2,2'-Bis[(3-methylphenyl)methylene]carbonimidic dihydrazide hydrochloride (**15**)*

Synthesized according to General Method A from 3-methylbenzaldehyde to afford the title compound as a white solid (78%). MP: 228–230°C. ^{1}H NMR (400 MHz, DMSO-d_6) δ 12.23 (br. s, 2H), 8.49 (br. s, 2H), 8.40 (br. s, 2H), 7.77 (s, 2H), 7.71 (d, *J* = 7.6 Hz, 2H), 7.37 (t, *J* = 7.6 Hz, 2H), 7.30 (d, *J* = 7.6 Hz, 2H), 2.37 (s, 6H); ^{13}C DEPTQ-135 NMR (101 MHz, DMSO-d_6) δ 152.8, 149.0 (2 × C), 138.1 (2 × C), 133.3 (2 × C), 131.5 (2 × C), 128.7 (2 × C), 128.1 (2 × C), 125.3 (2 × C), 20.9 (2 × C); LRMS (ESI) *m/z*: 294 [*M*-Cl]$^+$, 292 [*M*-HCl-H]$^-$.

*2,2'-Bis[(2-methylphenyl)methylene]carbonimidic dihydrazide hydrochloride (**16**)*

Synthesized according to General Method B from 2-methylbenzaldehyde to afford the title compound as a white solid (96%). MP: 266–268°C. ^{1}H NMR (400 MHz, DMSO-d_6) δ 12.22 (br. s, 2H), 8.78 (s, 2H), 8.45 (s, 2H), 8.19 (d, *J* = 7.7 Hz, 2H), 7.39 – 7.35 (m, 2H), 7.31 – 7.27 (m, 4H), 2.45 (s, 6H); ^{13}C DEPTQ-135 NMR (101 MHz, DMSO-d_6) δ 152.5, 147.5 (2 × C), 137.5 (2 × C), 131.3 (2 × C), 130.8 (2 × C), 130.6 (2 × C), 126.4 (2 × C), 126.1 (2 × C), 18.8 (2 × C); LRMS (ESI) *m/z*: 294 [*M*-Cl]$^+$, 292 [*M*-HCl-H]$^-$.

*2,2'-Bis([1,1'-biphenyl]-4-ylmethylene)carbonimidic dihydrazide hydrochloride (**17**)*

Synthesized according to General Method B from 4-phenylbenzaldehyde to afford the title compound as a yellow solid (81%). MP: 264–266°C. ^{1}H NMR (300 MHz, DMSO-d_6) δ 12.48 (br. s, 2H), 8.62 (s, 2H), 8.51 (s, 2H), 8.04 (d, *J* = 7.5 Hz, 4H), 7.85 – 7.69 (m, 8H), 7.54 – 7.36 (m, 6H); ^{13}C NMR (75 MHz, DMSO-d_6) δ 152.8, 148.4 (2 × C), 142.2 (2 × C), 139.3 (2 × C), 132.5 (2 × C), 129.1 (4 × C), 128.5 (4 × C), 128.0 (2 × C), 127.0 (4 × C), 126.8 (4 × C); HRMS: *m/z* (calcd for $C_{27}H_{23}N_5$, 417.1953; found 417.1831).

*2,2'-Bis([1,1'-biphenyl]-2-ylmethylene)carbonimidic dihydrazide hydrochloride (**18**)*

Synthesized according to General Method B from biphenyl-2-carboxaldehyde to afford the title compound as a yellow solid (89%). MP: 129−133 °C. ^{1}H NMR (400MHz DMSO-d_6) δ 12.27 (br. s, 2H), 8.49 (s, 2H), 8.44−8.27 (m, 4H), 7.57−7.44 (m, 10H), 7.41−7.33 (m, 6H); ^{13}C NMR (101 MHz DMSO-d_6) δ 152.6, 147.2 (2 × C), 142.4 (2 × C), 138.8 (2 × C), 130.6 (4 × C), 130.4 (2 × C), 129.7 (4 × C), 128.7 (4 × C), 127.8 (2 × C), 127.6 (2 × C), 126.8 (2 × C); HRMS: m/z calcd for $C_{27}H_{23}N_5$, 417.1953; found, 417.1709.

*2,2'-Bis[(4-propylphenyl)methylene]carbonimidic dihydrazide hydrochloride (**19**)*

Synthesized according to General Method B from 4-propylbenzaldehyde to afford the title compound as a white solid (77%). MP: 218–222°C. ^{1}H NMR (400 MHz, DMSO-*d_6*) δ 8.60 – 8.30 (m, 4H), 7.84 (d, *J* = 8.1 Hz, 4H), 7.28 (d, *J* = 8.1 Hz, 4H), 2.59 (t, *J* = 7.4 Hz, 4H), 1.64 – 1.54 (m, 4H), 0.88 (t, *J* = 7.4 Hz, 6H); ^{13}C NMR (101 MHz, DMSO-*d_6*) δ 152.8, 148.8 (2 × C), 145.3 (2 × C), 130.9 (2 × C), 128.7 (4 × C), 127.9 (4 × C), 37.2 (2 × C), 23.9 (2 × C), 13.6 (2 × C); HRMS: *m/z* (calcd for $C_{21}H_{27}N_5$, 349.2266; found 349.2112).

*2,2'-Bis[(4-butylphenyl)methylene]carbonimidic dihydrazide hydrochloride (**20**)*

Synthesized according to General Method B from 4-butylbenzaldehyde to afford the title compound as a pale yellow solid (80%). MP: 216–220°C. ^{1}H NMR (400 MHz, DMSO-*d_6*) δ 12.21 (br. s, 2H), 8.44 (s, 2H), 8.39 (s, 2H), 7.83 (d, *J* = 8.2 Hz, 4H), 7.30 (d, *J* = 8.2 Hz, 4H), 2.63 (t, *J* = 7.7 Hz, 4H), 1.61 – 1.52 (m, 4H), 1.36 – 1.26 (m, 4H), 0.90 (t, *J* = 7.3 Hz, 6H); ^{13}C NMR (101 MHz, DMSO-*d_6*) δ 152.7, 148.8 (2 × C), 145.5 (2 × C), 130.9 (2 × C), 128.7 (4 × C), 127.9 (4 × C), 34.8 (2 × C), 32.9 (2 × C), 21.7 (2 × C), 13.8 (2 × C). HRMS: *m/z* (calcd for $C_{23}H_{31}N_5$, 377.2579; found 377.2336).

*2,2'-Bis{[4-(1-methylethyl)phenyl]methylene}carbonimidic dihydrazide hydrochloride (**21**)*

Synthesized according to General Method B from 4-isopropylbenzaldehyde to afford the title compound as a white solid (28%). MP: 193–196°C. ^{1}H NMR (400 MHz, DMSO-*d_6*) δ 8.62 – 8.29 (m, 4H), 7.85 (d, *J* = 8.2 Hz, 4H), 7.33 (d, *J* = 8.2 Hz, 4H), 2.98 – 2.87 (m, 2H), 1.21 (d, *J* = 6.9 Hz, 12H); ^{13}C NMR (101 MHz, DMSO-*d_6*) δ 152.7, 151.4 (2 × C), 148.8 (2 × C), 131.0 (2 × C), 128.0 (4 × C), 126.7 (4 × C), 33.4 (2 × C), 23.6 (4 × C); HRMS: *m/z* (calcd for $C_{21}H_{27}N_5$, 349.2266; found 349.2112).

*2,2'-Bis{[4-(1,1-dimethylethyl)phenyl]methylene}carbonimidic dihydrazide hydrochloride (**22**)*

Synthesized according to General Method B from 4-*tert*-butylbenzaldehyde to afford the title compound as a white solid (50%). MP: 150–155°C. ^{1}H NMR (300 MHz, DMSO-*d_6*) δ 8.56 – 8.32 (m, 4H), 7.85 (d, *J* = 8.3 Hz, 4H), 7.49 (d, *J* = 8.3 Hz, 4H), 1.31 (s, 18H); ^{13}C NMR (75 MHz, DMSO-*d_6*) δ 153.7 (2 × C), 152.7, 148.8 (2 × C), 130.7 (2 × C), 127.8 (4 × C), 125.6 (4 × C), 34.7 (2 × C), 31.0 (6 × C); HRMS: *m/z* (calcd for $C_{23}H_{31}N_5$, 377.2579; found 377.2454).

*2,2'-Bis[(3-ethynylphenyl)methylene]carbonimidic dihydrazide hydrochloride (**23**)*

Synthesized according to General Method B from 4-ethynylbenzaldehyde to afford the title compound as a yellow solid (93%). MP: 220°C (Decomp.). ^{1}H NMR (400 MHz, DMSO-*d_6*) δ 12.27 (br. s, 2H), 8.58 (s, 2H), 8.42 (s, 2H), 7.96 (d, *J* = 8.3 Hz, 4H), 7.58 (d, *J* = 8.3 Hz, 4H), 4.39 (s, 2H); ^{13}C NMR (101 MHz, DMSO-*d_6*) δ 152.9, 148.0 (2 × C), 133.7 (2 × C), 132.0 (4 × C), 128.0 (4 × C), 123.7 (2 × C), 83.2 (2 × C), 83.0 (2 × C); LRMS: *m/z* (calcd for $C_{19}H_{15}N_5$, 313.13; found 313.80).

*2,2'-Bis[(4-methoxyphenyl)methylene]carbonimidic dihydrazide hydrochloride (**24**)*

Synthesized according to General Method A from 4-methoxybenzaldehyde to afford the title compound as a white solid. MP: 218–220°C. ^{1}H NMR (400 MHz, DMSO-*d_6*) δ 12.00 (br. s, 2H), 8.34 (br. s, 4H), 7.87 (d, *J* = 8.8 Hz, 4H), 7.04 (d, *J* = 8.8 Hz, 4H), 3.83 (s, 6H); ^{13}C DEPTQ-135 NMR (DMSO-*d_6*) δ 161.4 (2 × C), 152.6, 148.5 (2 × C), 129.6 (4 × C), 125.9 (2 × C), 114.2 (4 × C), 55.4 (2 × C); LRMS: (ESI+) m/z: 326 ($C_{17}H_{20}N_5O_2$) [*M*-Cl]$^{+}$; (ESI-) m/z: 324 ($C_{17}H_{18}N_5O_2$) [*M*-HCl-H]$^{-}$.

*2,2'-Bis[(3-methoxyphenyl)methylene]carbonimidic dihydrazide hydrochloride (**25**)*

Synthesized according to General Method A from 3-methoxybenzaldehyde to afford the title compound as a white solid (87%). MP: 204–206 °C. ^{1}H NMR (400 MHz, DMSO-*d_6*) δ 12.16 (br. s, 2H), 8.53 (br. s, 2H), 8.39 (br. s, 2H), 7.57 (s, 2H), 7.46–7.38 (m, 4H), 7.07 (dd, *J* = 8.0, 1.5 Hz, 2H), 3.84 (s, 6H); ^{13}C DEPTQ-135 NMR (101 MHz, DMSO-*d_6*) δ 159.6 (2 × C), 152.8,

148.8 (2 × C), 134.6 (2 × C), 129.9 (2 × C), 120.9 (2 × C), 116.7 (2 × C), 112.3 (2 × C), 55.4 (2 × C); LRMS: (ESI+) m/z: 326 ($C_{17}H_{20}N_5O_2$) [*M*-Cl]$^+$; (ESI-) m/z: 324 ($C_{17}H_{19}N_5O_2$) [*M*-HCl-H]$^-$

*2,2'-Bis[(2-methoxyphenyl)methylene]carbonimidic dihydrazide hydrochloride (**26**)*

Synthesized according to General Method A from 2-methoxybenzaldehyde to afford the title compound as a white solid (70%). MP: 234–236°C. ^{1}H NMR (400 MHz, DMSO-d_6) δ 12.16 (br. s, 2H), 8.74 (s, 2H), 8.41 (s, 2H), 8.22 (dd, *J* = 7.6, 1.6 Hz, 2H), 7.48 (dt, *J* = 7.9, 1.8 Hz, 2H), 7.14 (d, *J* = 8.0 Hz, 2H), 7.05 (t, *J* = 7.6 Hz, 2H), 3.88 (s, 6H); ^{13}C NMR (101 MHz, DMSO-d_6) δ 158.0 (2 × C), 152.5, 144.3 (2 × C), 132.5 (2 × C), 126.6 (2 × C), 121.2 (2 × C), 120.6 (2 × C), 111.9 (2 × C), 55.8 (2 × C); LRMS: (ESI+) m/z: 326 ($C_{17}H_{20}N_5O_2$) [*M*-Cl]$^+$; (ESI-) m/z: 324 ($C_{17}H_{18}N_5O_2$) [*M*-HCl-H]$^-$.

*2,2'-Bis[(4-hydroxyphenyl)methylene]carbonimidic dihydrazide hydrochloride (**27**)*

Synthesized according to General Method B from 4-hydroxybenzaldehyde to afford the title compound as a yellow solid (18%). MP: 246–248°C. ^{1}H NMR (400 MHz, DMSO-d_6) δ 11.92 (br. s, 2H), 10.13 (br. s, 2H), 8.28 (s, 4H), 7.75 (d, *J* = 8.5 Hz, 4H), 6.86 (d, *J* = 8.5 Hz, 4H); ^{13}C NMR (101 MHz, DMSO-d_6) δ 160.1 (2 × C), 152.4, 148.8 (2 × C), 129.6 (4 × C), 124.3 (2 × C), 115.6 (4 × C); LRMS: *m/z* (calcd for $C_{15}H_{15}N_5O_2$, 297.12; found 297.90).

*2,2'-Bis[(3-hydroxyphenyl)methylene]carbonimidic dihydrazide hydrochloride (**28**)*

Synthesized according to General Method B from 3-hydroxybenzaldehyde to afford the title compound as a pale yellow solid (61%). M.P. 121–123°C. ^{1}H NMR (300 MHz, DMSO-d_6) δ 8.00 (s, 2H), 7.26 – 7.08 (m, 6H), 6.98 – 6.43 (m, 4H); ^{13}C DEPTQ-135 NMR (75 MHz, DMSO-d_6)* δ 157.6 (2 × C), 143.8 (2 × C), 136.8 (2 × C), 129.5 (2 × C), 118.0 (2 × C), 116.1 (2 × C), 113.0 (2 × C); HRMS: *m/z* (calcd for $C_{15}H_{15}N_5O_2$, 297.1226; found 297.1114). * Carbon imide signal not observed.

*2,2'-Bis[(2-hydroxyphenyl)methylene]carbonimidic dihydrazide hydrochloride (**29**)*

Synthesized according to General Method B from 2-hydroxybenzaldehyde as a white solid (95%). MP: 263–266°C. ^{1}H NMR (300 MHz, DMSO-d_6) δ 12.10 (br. s, 2H), 10.25 (s, 2H), 8.70 (s, 2H), 8.36 (s, 2H), 8.07 (d, *J* = 7.6 Hz, 2H)*, 7.29 (t, *J* = 7.4 Hz, 2H), 6.97 (d, *J* = 8.1 Hz, 2H), 6.88 (t, *J* = 7.4 Hz, 2H); ^{13}C NMR (75 MHz, DMSO-d_6) δ 156.8 (2 × C), 152.4, 145.3 (2 × C), 132.1 (2 × C), 126.9 (2 × C), 119.6 (2 × C), 119.2 (2 × C), 116.2 (2 × C); HRMS: *m/z* (calcd for $C_{15}H_{15}N_5O_2$, 297.1226; found 297.1125). * Poorly resolved doublet gives reduced coupling constant.

*2,2'-Bis{[4-(trifluoromethoxy)phenyl]methylene}carbonimidic dihydrazide hydrochloride (**30**)*

Synthesized according to General Method B from 4-trifluoromethoxybenzaldehyde to afford the title compound as a white solid (69%). MP: 258–261°C. ^{1}H NMR (400 MHz, DMSO-d_6) δ 12.49 (br. s, 2H), 8.65 (s, 2H), 8.49 (s, 2H), 8.10 (d, *J* = 8.6 Hz, 4H), 7.47 (d, *J* = 8.3 Hz, 4H); ^{13}C NMR (101 MHz, DMSO-d_6) δ 153.0, 149.8 (d, $^3J_{CF3}$ = 1.6 Hz, 2 × C), 147.4 (2 × C), 132.6 (2 × C), 129.9 (4 × C), 121.2 (4 × C), 120.0 (q, $^1J_{CF3}$ = 256.9 Hz, 2 × C); LRMS: *m/z* (calcd for $C_{17}H_{13}F_6N_5O_2$, 433.10; found 433.80).

*2,2'-Bis{[4-(methylsulfanyl)phenyl]methylene}carbonimidic dihydrazide hydrochloride (**31**)*

Synthesized according to General Method B from 4-thiomethylbenzaldehyde to afford the title compound as an off-white solid (73%). MP: 242–245°C. ^{1}H NMR (400 MHz, DMSO-d_6) δ 12.17 (br. s, 2H), 8.46 (s, 2H), 8.37 (s, 2H), 7.86 (d, *J* = 8.2 Hz, 4H), 7.34 (d, *J* = 8.2 Hz, 4H), 2.53 (s, 6H); ^{13}C DEPTQ-135 NMR (101 MHz, DMSO-d_6) δ 152.7, 148.4 (2 × C), 142.0 (2 × C), 129.7 (2 × C), 128.2 (4 × C), 125.4 (4 × C), 14.2 (2 × C); LRMS: *m/z* (calcd for $C_{17}H_{19}N_5S_2$, 357.11; found 357.95).

*2,2'-Bis{[4-(trifluoromethylsulfanyl)phenyl]methylene}carbonimidic dihydrazide hydrochloride (**32**)*

Synthesized according to General Method B from 4-thiotrifluoromethylbenzaldehyde to afford the title compound as a white solid (48%). MP: 195°C (Decomp.). ^{1}H NMR (400 MHz, DMSO-d_6) δ 12.68 (br. s, 2H), 8.73 (s, 2H), 8.53 (s, 2H), 8.10 (d,

J = 8.2 Hz, 4H), 7.79(d, J = 8.2 Hz, 4H); ^{13}C DEPTQ-135 NMR (101 MHz, DMSO-d_6) δ 153.1, 147.4 (2 × C), 136.20 (4 × C), 136.16 (2 × C), 129.5 (q, $^{1}J_{CF3}$ = 309.2 Hz, 2 × C), 129.1 (4 × C), 125.2 – 125.1 (Unresolved quartet, 2 × C); LRMS: *m/z* (calcd for $C_{17}H_{13}F_6N_5S_2$, 465.05; found 465.60).

*2,2'-Bis{4-(dimethylamino)phenyl]methylene}carbonimidic dihydrazide hydrochloride (**33**)*

Synthesized according to General Method B from 4-(dimethylamino)benzaldehyde to afford the title compound as an orange solid (80%). MP: 161–164°C. ^{1}H NMR (400 MHz, DMSO-d_6) δ 11.92 (br. s, 2H), 8.24 (s, 2H), 8.16 (s, 2H), 7.71 (d, J = 8.9 Hz, 4H), 6.74 (d, J = 8.9 Hz, 4H), 2.98 (s, 12H); ^{13}C NMR (101 MHz, DMSO-d_6) δ 152.1, 151.8 (2 × C), 149.0 (2 × C), 129.2 (4 × C), 120.5 (2 × C), 111.5 (4 × C), 39.7 (4 × C); HRMS: *m/z* (calcd for $C_{19}H_{25}N_7$, 351.2171; found 351.1966).

*2,2'-Bis[(2,4-dichlorophenyl)methylene]carbonimidic dihydrazide hydrochloride (**34**)*

Synthesized according to General Method B from 2,4-dichlorobenzaldehyde to afford the title compound as a yellow solid (47%). MP: 277–280°C. ^{1}H NMR (DMSO-d_6) δ 12.84 (br. s, 2H), 8.84 (s, 2H), 8.74 (s, 2H), 8.46 (d, J = 8.6 Hz, 2H), 7.71 (d, J = 1.4 Hz, 2H), 7.54 (dd, J = 8.6, 1.4 Hz, 2H); ^{13}C NMR (DMSO-d_6) δ 152.7 (2 × C), 144.0, 135.9 (2 × C), 134.3 (2 × C), 129.7 (2 × C), 129.3 (2 × C), 129.3 (2 × C), 127.8 (2 × C); LRMS: (ESI+) m/z: 404 ($C_{15}H_{11}Cl_4N_5$) [M -Cl]$^+$.

*2,2'-Bis[(3,5-dichlorophenyl)methylene]carbonimidic dihydrazide hydrochloride (**35**)*

Synthesized according to General Method B from 3,5-dichlorobenzaldehyde to afford the title compound as a white solid (84%). MP: 277–280°C. ^{1}H NMR (DMSO-d_6) δ 12.67 (br. s, 2H), 8.81 (s, 2H), 8.40 (s, 2H), 8.06 (d, J = 1.8 Hz, 4H), 7.68 (t, J = 1.8 Hz, 2H); ^{13}C NMR (DMSO-d_6) δ 153.1, 146.1 (2 × C), 136.9 (2 × C), 134.6 (4 × C), 129.7 (2 × C), 126.2 (4 × C); LRMS: (ESI+) m/z: 404 ($C_{15}H_{12}Cl_4N_5$) [M-Cl]$^+$; (ESI-) m/z: 402 ($C_{15}H_{10}Cl_4N_5$) [M-HCl-H]$^-$.

*2,2'-Bis[(2,6-dichlorophenyl)methylene]carbonimidic dihydrazide hydrochloride (**36**)*

Synthesized according to General Method B from 2,6-dichlorobenzaldehyde to afford the title compound as a white solid (52mg, 63%). MP: 215–217°C. ^{1}H NMR (DMSO-d_6) δ 12.58 (br. s, 2H), 8.62 (s, 2H), 8.21 (s, 2H), 7.60 (d, J = 8.0 Hz, 4H), 7.51 – 7.47 (m, 2H); ^{13}C NMR (101 MHz, DMSO-d_6) δ 153.0 (2 × C), 145.1, 134.1 (4 × C), 131.9 (2 × C), 129.9 (2 × C), 129.0 (4 × C); LRMS: (ESI+) m/z: 404 ($C_{15}H_{12}Cl_4N_5$) [M-Cl]$^+$; (ESI-) m/z: 402 ($C_{15}H_{10}Cl_5N_5$) [M-HCl-H]$^-$.

*2,2'-Bis[(2,5-difluorophenyl)methylene]carbonimidic dihydrazide hydrochloride (**37**)*

Synthesized according to General Method B from 2,5-difluorophenylbenzaldehyde to afford the title compound as a white solid (34%). MP: 276–278°C. ^{1}H NMR (DMSO-d$_6$) δ 12.83 (br. s, 2H), 8.76 (s, 2H), 8.69 (s, 2H), 8.31–8.21 (m, 2H), 7.45–7.31 (m, 4H); ^{13}C NMR (DMSO-d$_6$) δ 158.5 (dd, $^{1,4}J_{CF}$ = 240.2, 1.2 Hz, 2 × C), 157.2 (dd, $^{1,4}J_{CF}$ = 247.2, 1.4 Hz, 2 × C), 152.8, 140.7 (2 × C), 122.5 (dd, $^{2,3}J_{CF}$ = 12.2, 8.9 Hz, 2 × C), 119.4 (dd, $^{2,3}J_{CF}$ = 25.1, 9.0 Hz, 2 × C), 117.8 (dd, $^{2,3}J_{CF}$ = 24.0, 8.8 Hz, 2 × C), 113.0 (dd, $^{2,3}J_{CF}$ = 25.9, 2.3 Hz, 2 × C); HRMS: m/z calcd for $C_{15}H_{11}F_4N_5$, 337.0951; found, 338.0877.

*2,2'-Bis[(3,4-difluorophenyl)methylene]carbonimidic dihydrazide hydrochloride (**38**)*

Synthesized according to General Method B from 3,4-difluorobenzaldehyde to afford the title compound as a white solid (76%). MP: 286°C (Decomp.). ^{1}H NMR (DMSO-d$_6$) δ 8.68 (br. s, 2H), 8.43 (s, 2H), 8.28–8.16 (m, 2H), 7.77–7.64 (m, 2H), 7.58–7.46 (m, 2H); ^{13}C NMR (DMSO-d$_6$) δ 153.0, 150.8 (dd, $^{1,2}J_{CF}$ = 250.6, 13.0 Hz, 2 × C), 149.9 (dd, $^{1,2}J_{CF}$ = 245.9, 13.2 Hz, 2 × C), 146.6 (2 × C), 131.2 (dd, $^{3,4}J_{CF}$ = 6.4, 3.4 Hz, 2 × C), 126.0 (dd, $^{3,4}J_{CF}$ = 6.4, 2.8 Hz, 2 × C), 117.8 (d, $^{2}J_{CF}$ = 17.7 Hz, 2 × C), 115.7 (d, $^{2}J_{CF}$ = 18.5 Hz, 2 × C); HRMS: m/z calcd for $C_{15}H_{11}F_4N_5$, 337.0951; found, 338.0943.

*2,2'-Bis[(2,3,4,5,6-pentafluorophenyl)methylene]carbonimidic dihydrazide hydrochloride (**39**)*

Synthesized according to General Method B from 2,3,4,5,6-pentafluorobenzaldehyde to afford the title compound as a white solid (60%). MP: 234−236 °C. ^{1}H NMR (DMSO-d_6) δ 8.64 (s, 2H), 8.36 (s, 2H); ^{13}C DEPTQ-135 NMR (DMSO-d_6) δ 152.8, 144.7 (d, J_{CF} = 259.3 Hz, 4 × C)*, 141.4 (d, J_{CF} = 254.8 Hz, 2 × C)*, 138.6 (2 × C), 137.4 (d, J_{CF} = 249.6 Hz, 4 × C)*, 108.9 (td, J_{CF} = 12.6, 3.8 Hz, 2 × C); HRMS: m/z calcd for $C_{15}H_5F_{10}N_5$, 445.0358; found, 446.0306. *Further splitting due to C-F coupling is observed but not quantifiable.

*2,2'-Bis[(2-acetylamino-4-chlorophenyl)methylene]carbonimidic dihydrazide hydrochloride (**40**)*

A solution of 2-amino-4-chlorobenzaldehyde (204.8 mg, 1.316 mmol) and acetic anhydride (1.0 mL, 1.08 g, 11.9 mmol, 9.1 eq.) in CH_2Cl_2 (25 mL) was heated at reflux for 62 h. The solution was then concentrated *in vacuo* to afford *N*-(5-chloro-2-formylphenyl)acetamide as an orange/brown crystalline material that was used without further purification. The title compound was synthesized according to General Method A from crude *N*-(5-chloro-2-formylphenyl)acetamide (2.3 eq.). The crude product was recrystallised from EtOH to afford the title compound as an off-white crystalline solid (18%, 2 steps). MP: 259°C decomp. ^{1}H NMR (DMSO-d_6) δ 12.29 (s, 1H), 10.29 (s, 1H), 8.55 (s, 1H), 8.45 (s, 1H), 8.13 (d, *J* = 8.5 Hz, 1H), 7.71 (s, 1H), 7.35 (dd, *J* = 8.5, 1.8 Hz, 1H), 2.12 (s, 3H); ^{13}C NMR (DMSO-d_6) δ 169.2 (2 × C), 152.8, 146.4 (2 × C), 138.4 (2 × C), 135.0 (2 × C), 129.7 (2 × C), 125.0 (2 × C), 124.7 (2 × C), 124.3 (2 × C), 23.6 (2 × C); LRMS ESI –ve m/z 448 (M-Cl)$^+$.

*2,2′-Bis[(2-bromo-4,5-dimethoxyphenyl)methylene]carbonimidic dihydrazide hydrochloride (**41**)*

Synthesized according to General Method B from 2-bromo-4,5-dimethoxybenzaldehyde to afford the title compound as a white solid (83%). MP: 233−235 °C. ^{1}H NMR (400 MHz, DMSO-d_6) δ 12.36 (br. s, 2H), 8.69 (br. s, 2H), 8.58 (br. s, 2H), 7.81 (s, 2H), 7.24 (s, 2H), 3.89 (s, 6H), 3.85 (s, 6H); ^{13}C NMR (101 MHz, DMSO-d_6) δ 152.4, 151.9 (2 × C), 148.7 (2 × C), 147.5 (2 × C), 124.1 (2 × C), 115.9 (2 × C), 115.5 (2 × C), 110.1 (2 × C), 56.2 (2 × C), 56.1 (2 × C); HRMS: m/z calcd for $C_{19}H_{21}Br_2N_5O_4$, 542.9940; found, 543.9882.

*2,2′-Bis[(3-bromo-4,5-dimethoxyphenyl)methylene]carbonimidic Dihydrazide Hydrochloride (**42**)*

Synthesized according to General Method B from 3-bromo-4,5-dimethoxybenzaldehyde to afford the title compound as a white solid (45%). MP: 223−224 °C. ^{1}H NMR (DMSO-d_6) δ 8.66 (br. s, 2H), 8.37 (br. s, 2H), 7.79 (d, *J* = 1.6 Hz, 2H), 7.68−7.62 (m, 2H), 3.92 (s, 6H), 3.78 (s, 6H); ^{13}C NMR (DMSO-d_6) δ 153.6 (2 × C), 152.8, 147.5 (2 × C), 147.2 (2 × C), 130.7 (2 × C), 123.8 (2 × C), 117.1 (2 × C), 111.8 (2 × C), 60.3 (2 × C), 56.5 (2 × C); HRMS: m/z calcd for $C_{19}H_{21}Br_2N_5O_4$, 542.9940; found, 543.9850.

*2,2′-Bis{[4-(dimethylamino)-2-hydroxyphenyl]methylene}carbonimidic dihydrazide hydrochloride (**43**)*

Synthesized according to General Method A from 4-(dimethylamino)saliclaldehyde to afford the title compound as a brown solid (62%). MP: 254 °C (Decomp.). ^{1}H NMR (DMSO-d_6) δ 11.63 (s, 1H), 9.84 (s, 1H), 8.46 (s, 1H), 8.02 (s, 1H), 7.74 (d, J = 8.8 Hz, 1H), 6.30 (d, J = 7.4 Hz*, 1H), 6.17 (s, 1H), 2.94 (s, 6H); ^{13}C NMR (DMSO-d_6) δ 158.0 (2 × C), 153.1 (2 × C), 151.4, 146.4 (2 × C), 128.4 (2 × C), 107.5 (2 × C), 104.4 (2 × C), 97.7 (2 × C), 39.7 (4 × C)*. *Signal eclipsed by DMSO; LRMS: m/z calcd for $C_{19}H_{25}N_7O_2$, 383.21; found, 383.70. *Poorly resolved doublet gives reduced coupling constant.

*2,2′-Bis[(3,4-dimethoxyphenyl)methylene]carbonimidic dihydrazide hydrochloride (**44**)*

Synthesized according to General Method B from 3,4-dimethoxybenzaldehyde to afford the title compound as a pale yellow solid (99%). MP: 218−220 °C. ^{1}H NMR (DMSO-d_6) δ 12.09 (br. s, 2H), 8.44 (s, 2H), 8.34 (s, 2H), 7.63 (d, *J* = 1.5 Hz, 2H), 7.33 (dd, *J* = 8.4, 1.5 Hz, 2H), 7.03 (d, *J* = 8.4 Hz, 2H), 3.86 (s, 6H), 3.81 (s, 6H); ^{13}C NMR (DMSO-d_6) δ 152.5, 151.4 (2 × C), 149.2 (2 × C), 148.9 (2 × C), 126.0 (2 × C), 122.9 (2 × C), 111.4 (2 × C), 109.5 (2 × C), 55.9 (2 × C), 55.7 (2 × C); HRMS: m/z calcd for $C_{19}H_{23}N_5O_4$, 385.1750; found, 386.1739.

*2,2'-Bis[(4-hydroxy-3-nitrophenyl)methylene]carbonimidic Dihydrazide Hydrochloride (**45**)*

Synthesized according to General Method B from 4-hydroxy-3-nitrobenzaldehyde to afford the title compound as a white solid (80%). MP: 269 °C (dec). ^{1}H NMR (DMSO-d_6) δ 8.56 (s, 2H), 8.42 (d, *J* = 2.0 Hz, 2H), 8.38 (s, 2H), 8.09 (dd, *J* = 8.7, 2.0 Hz, 2H), 7.29 (d, *J* = 8.7 Hz, 2H); ^{13}C NMR (DMSO-d_6) δ 153.6 (2 × C), 152.8, 146.9 (2 × C), 137.6 (2 × C), 133.7 (2 × C), 124.7 (2 × C), 124.4 (2 × C), 119.3 (2 × C); HRMS: m/z calcd for $C_{15}H_{13}N_7O_6$, 388.0917; found, 387.0927.

*2,2'-Bis[(3-hydroxy-4-methoxyphenyl)methylene]carbonimidic dihydrazide hydrochloride (**46**)*

Synthesized according to General Method B from 3-hydroxy-4-methoxybenzaldehyde to afford the title compound as a yellow solid (61%). MP: 158−161 °C. ^{1}H NMR (DMSO-d_6) δ 9.21 (br. s, 2H), 8.42−8.17 (m, 4H), 7.43 (d, *J* = 1.9 Hz, 2H), 7.25 (dd, *J* = 8.4, 1.9 Hz, 2H),6.99 (d, *J* = 8.4 Hz, 2H), 3.83 (s, 6H); ^{13}C NMR (DMSO-d_6) δ 152.5, 150.3 (2 × C), 148.9 (2 × C), 146.7 (2 × C), 126.2 (2 × C), 120.9 (2 × C), 113.8 (2 × C), 111.7 (2 × C), 55.7 (2 × C); HRMS: m/z calcd for $C_{17}H_{19}N_5O_4$, 357.1437; found, 358.1371.

*2,2'-Bis[(4-hydroxy-3-methoxyphenyl)methylene]carbonimidic dihydrazide hydrochloride (**47**)*

Synthesized according to General Method B from 4-hydroxy-3-methoxybenzaldehyde to afford the title compound as a yellow solid (69%). MP: 200−203 °C. ^{1}H NMR (DMSO-d_6) δ 9.77 (br. s, 2H), 8.36 (s, 2H), 8.29 (s, 2H), 7.58 (d, *J* = 1.5 Hz, 2H), 7.23 (dd, *J* = 8.1, 1.2 Hz, 2H), 6.87 (d, *J* = 8.1 Hz, 2H), 3.86 (s, 6H); ^{13}C NMR (DMSO-d_6) δ 152.4, 149.7 (2 × C), 149.0 (2 × C), 148.1 (2 × C), 124.7 (2 × C), 122.9 (2 × C), 115.4 (2 × C), 110.4 (2 × C), 55.9 (2 × C); HRMS: m/ z calcd for $C_{17}H_{19}N_5O_4$, 357.1437; found, 358.1447.

2,2'-Bis[(4-chlorophenyl)methylene]carbonimidic dihydrazide hydrochloride (**4**)

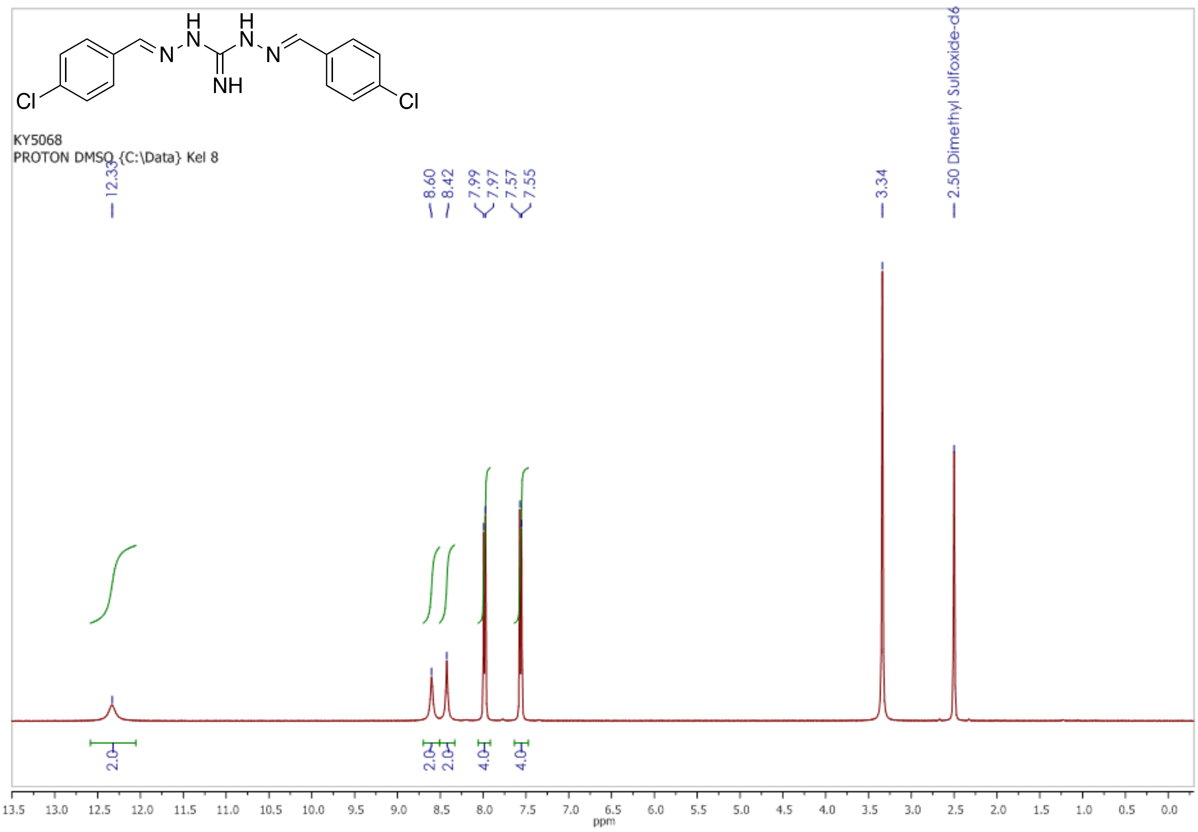

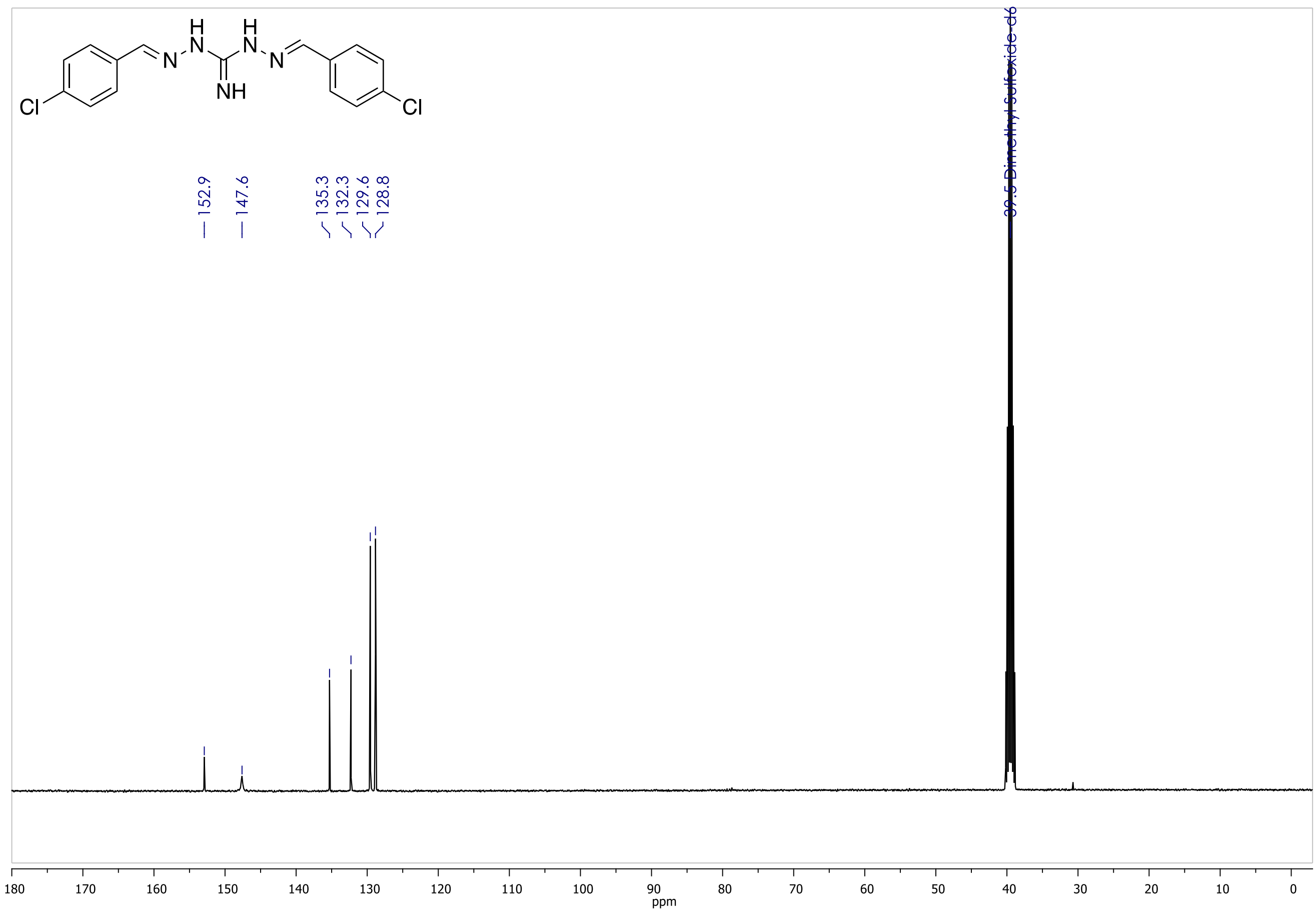

2,2'-Bis(phenylmethylene)carbonimidic dihydrazide hydrochloride (**5**)

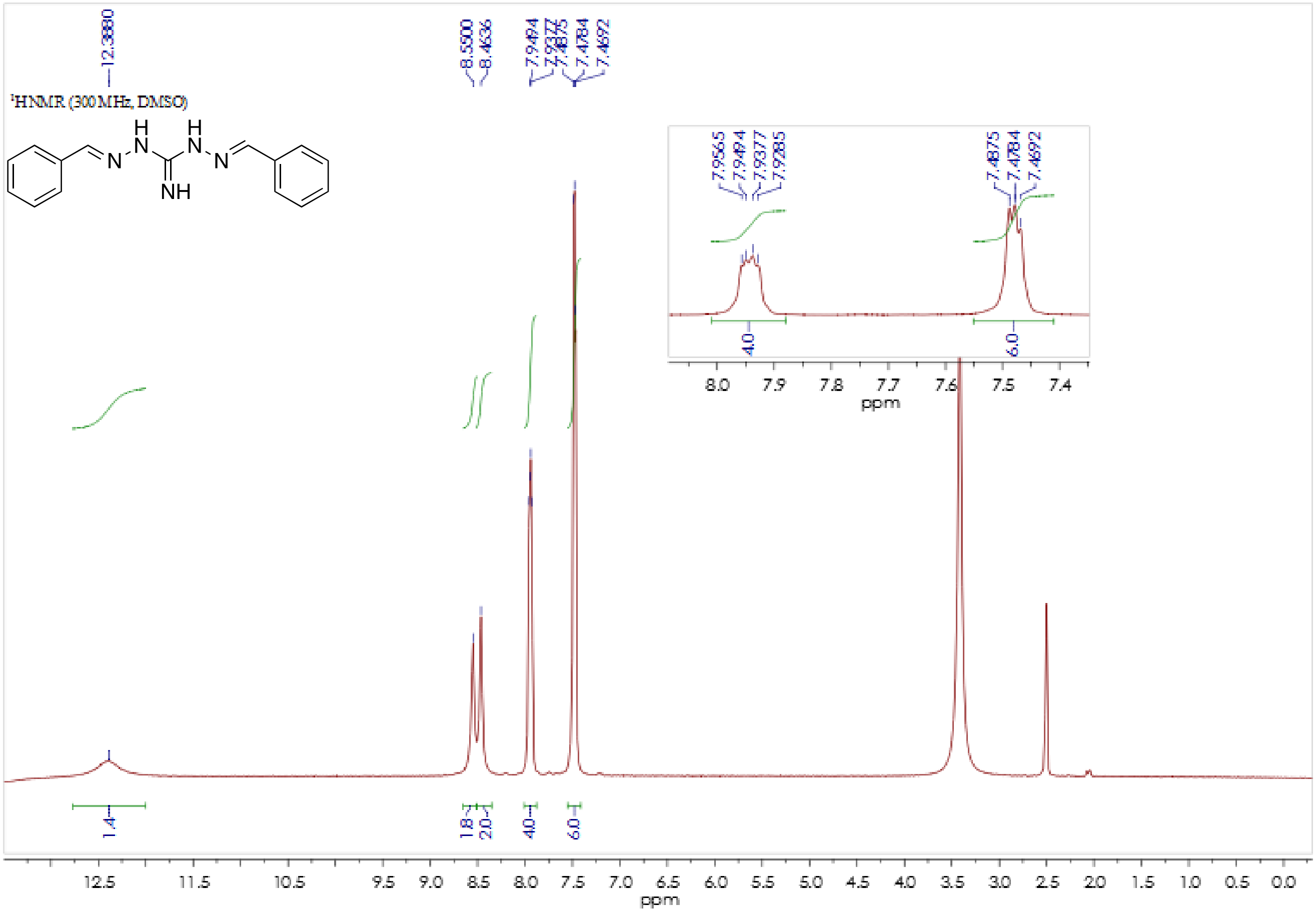

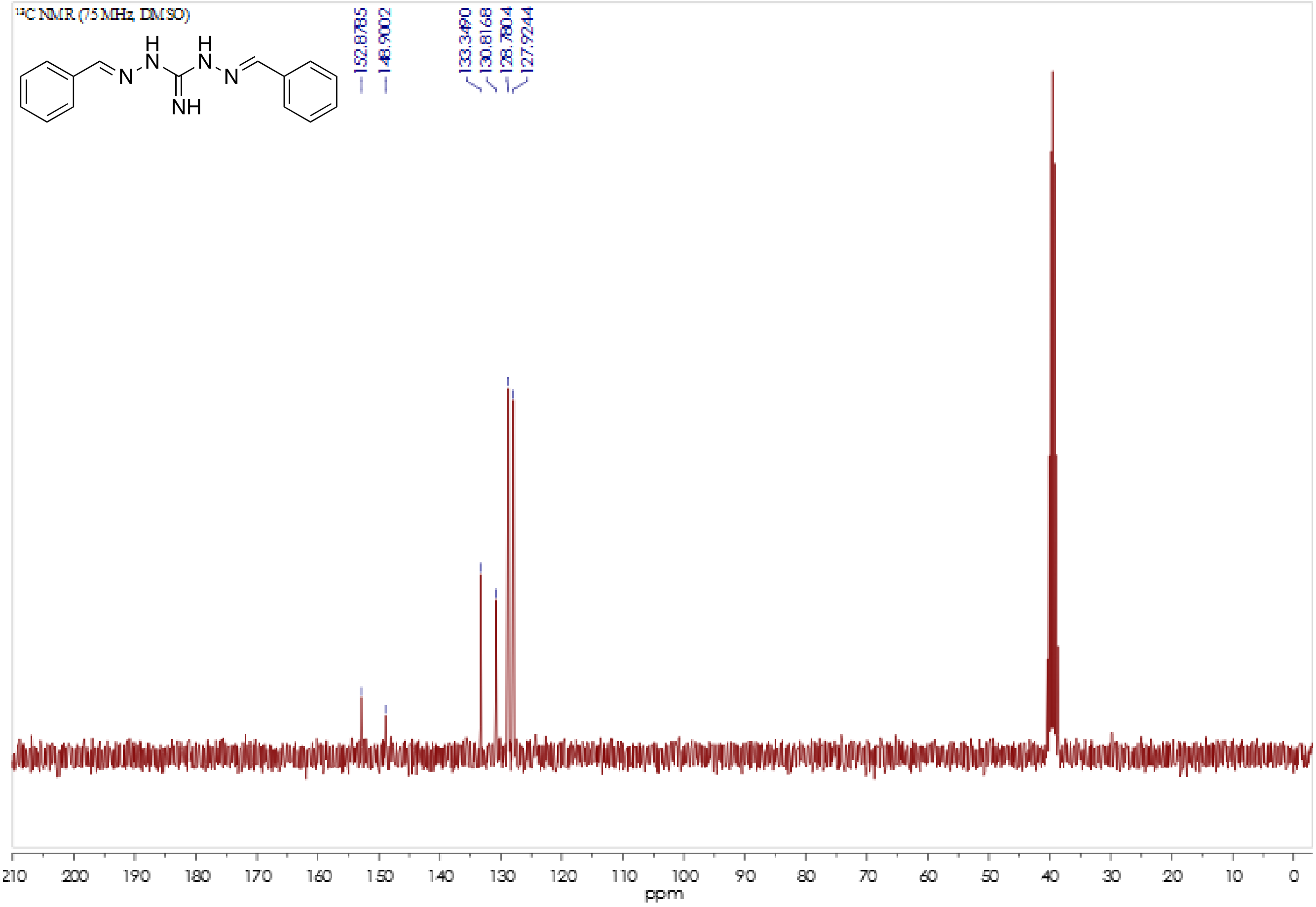

2,2'-Bis[(4-bromophenyl)methylene]carbonimidic dihydrazide hydrochloride (**6**)

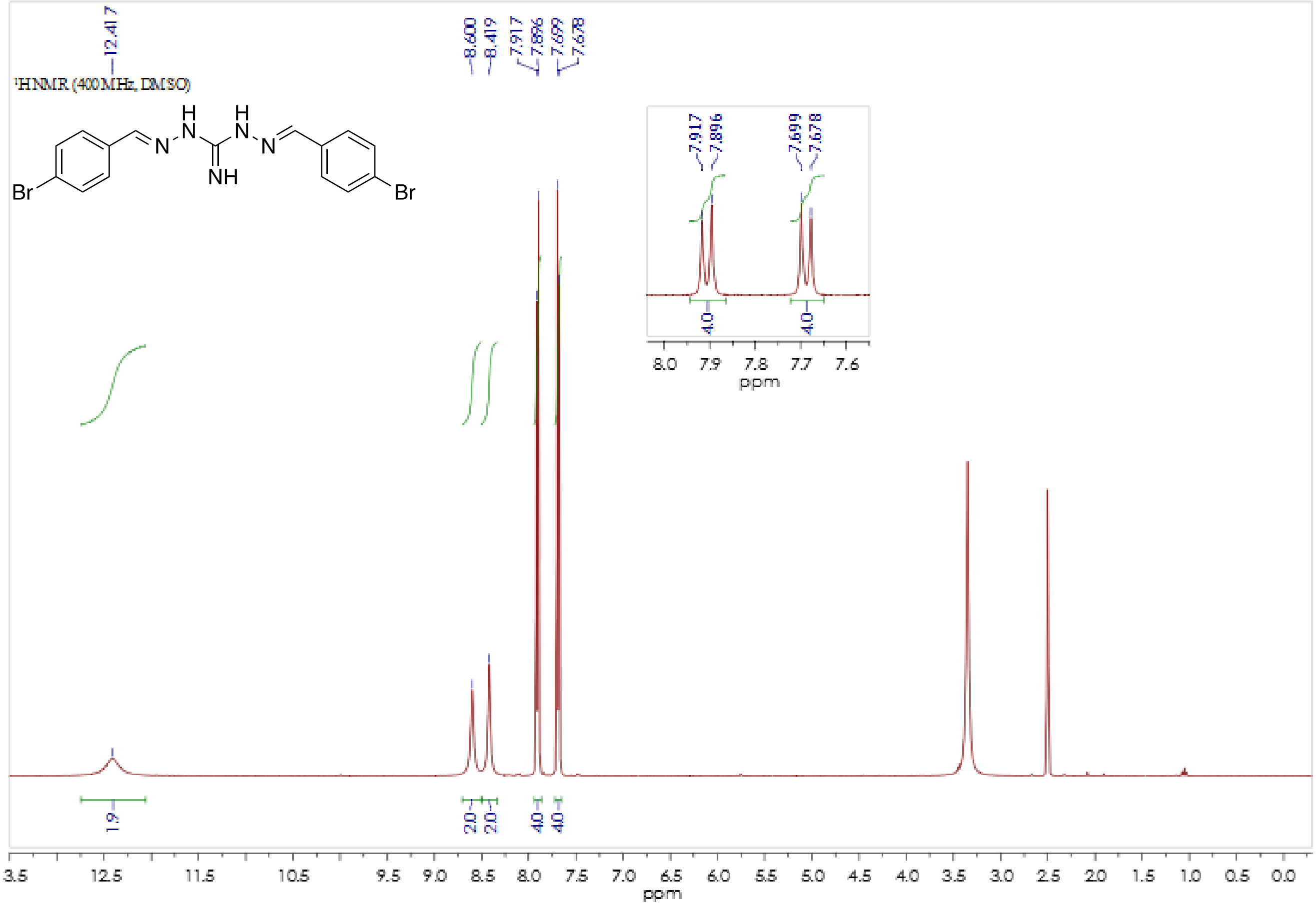

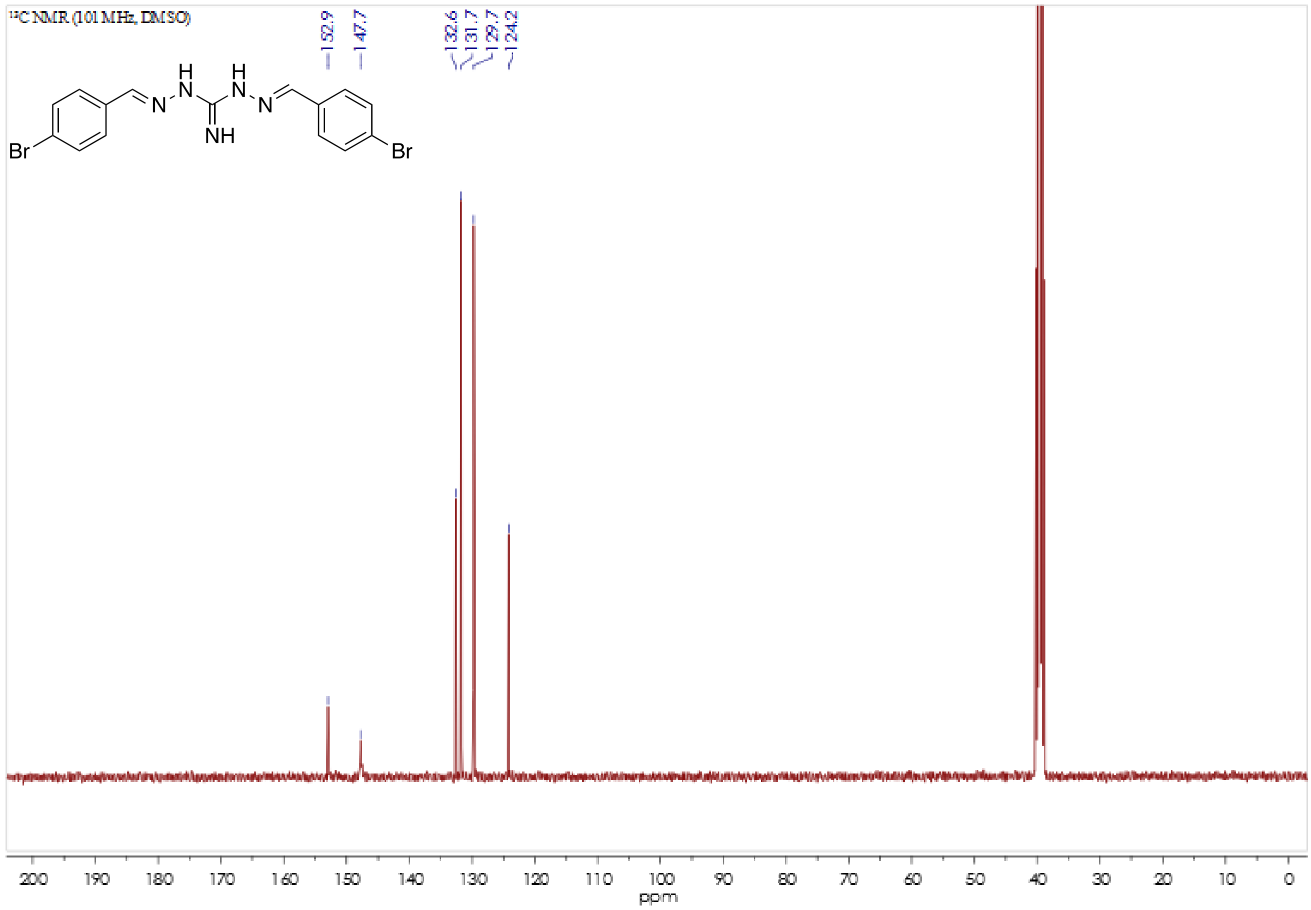

2,2'-Bis[(4-fluorophenyl)methylene]carbonimidic dihydrazide hydrochloride (**7**)

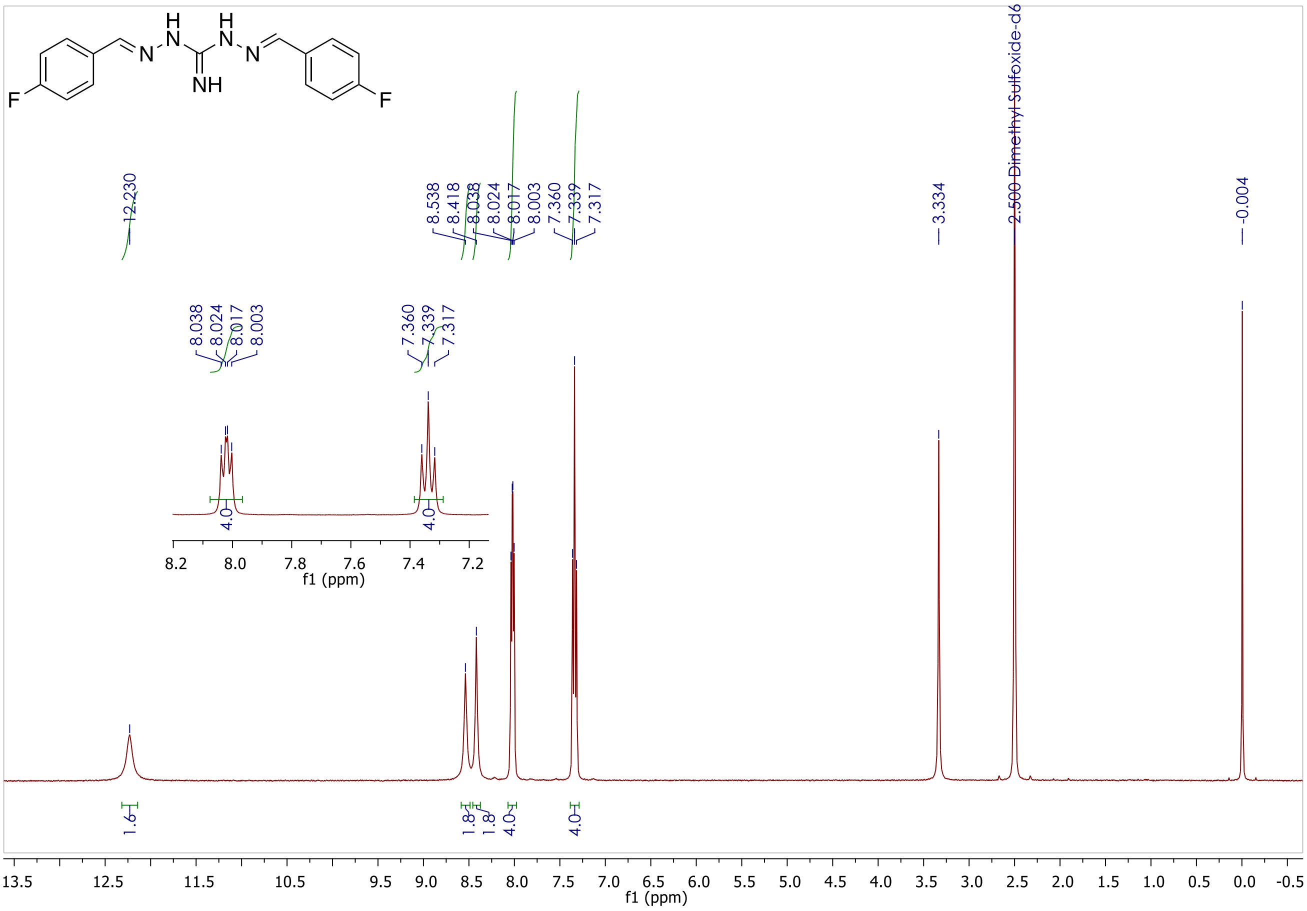

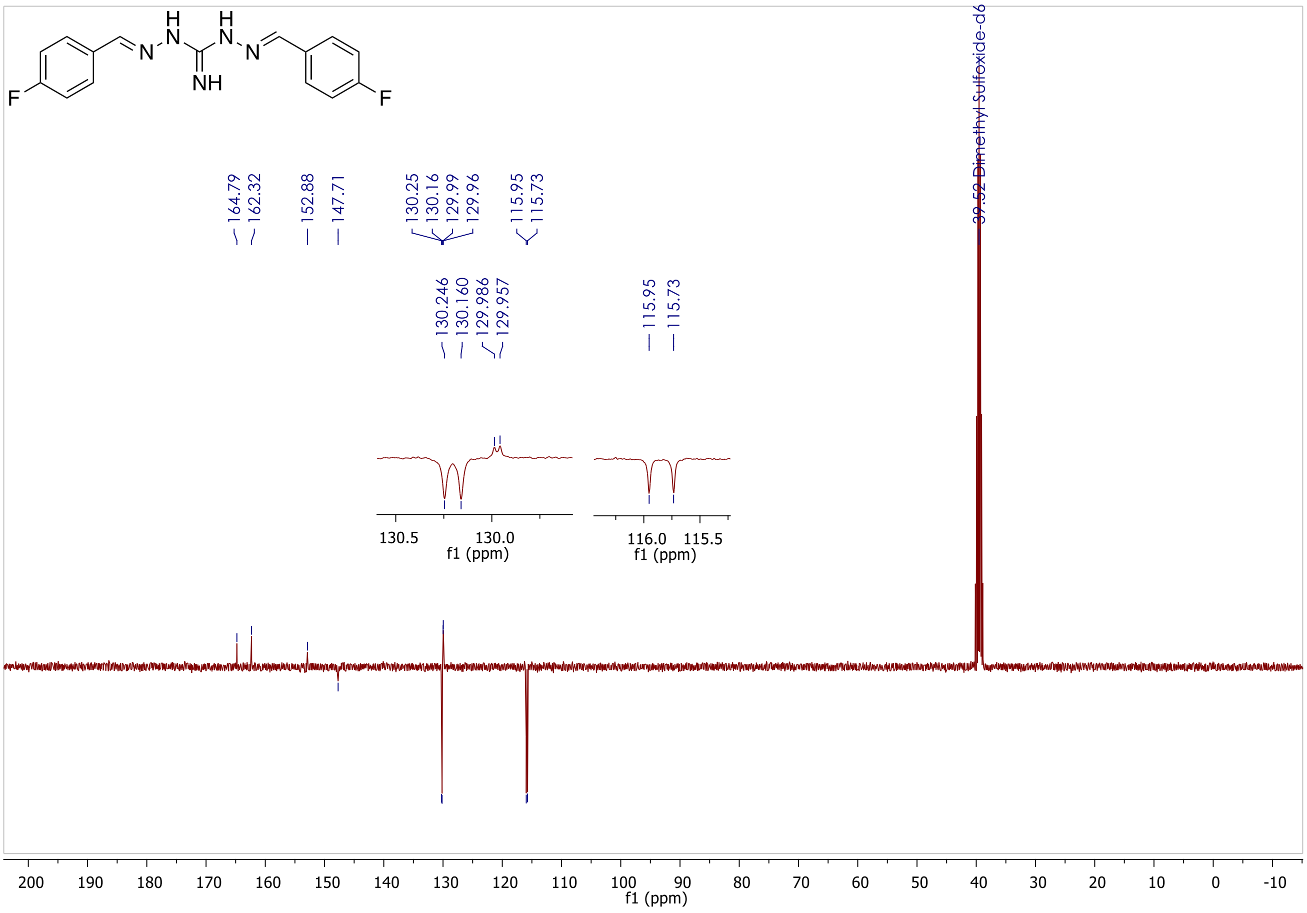

2,2'-Bis[(3-chlorophenyl)methylene]carbonimidic dihydrazide hydrochloride (**8**)

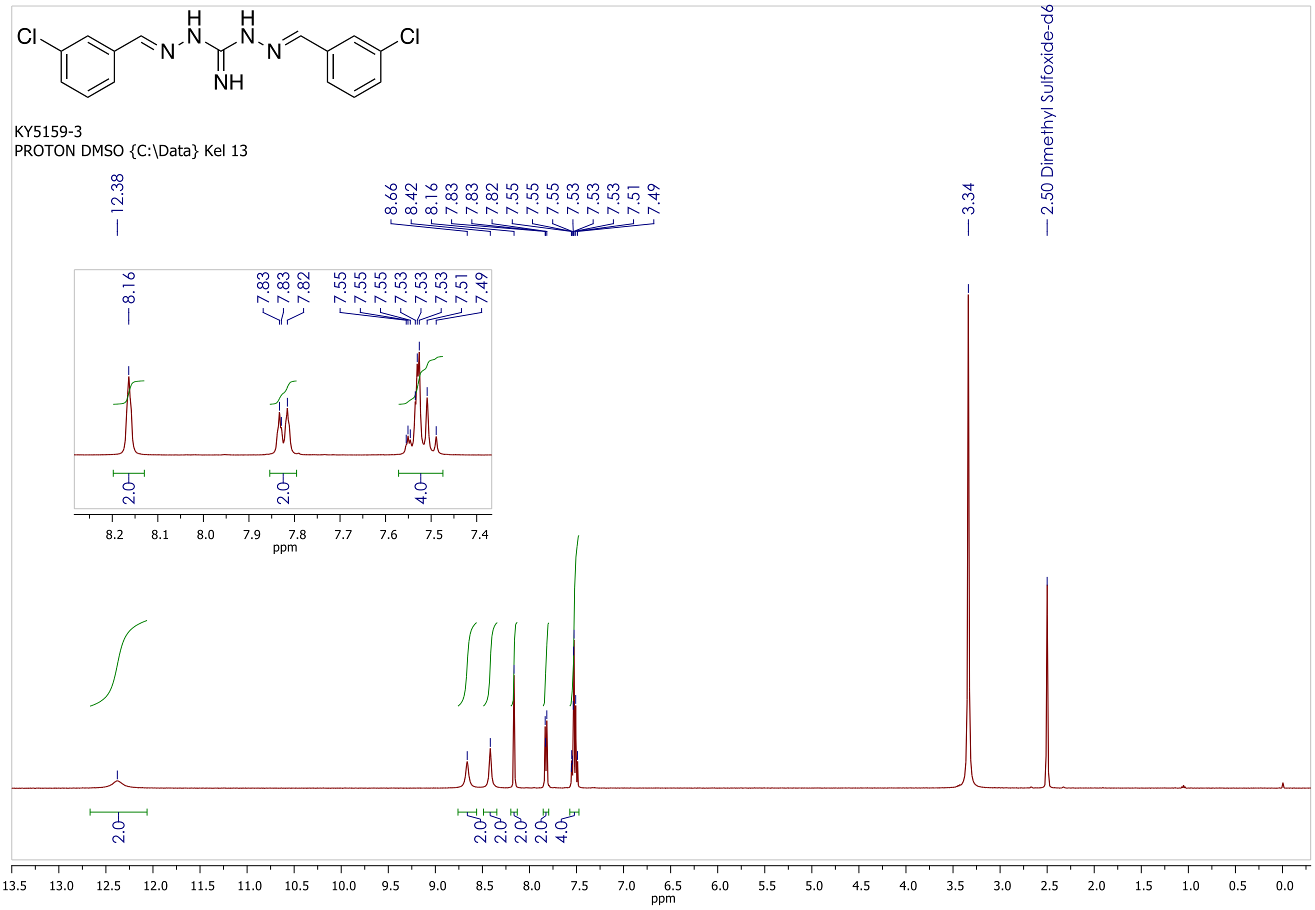

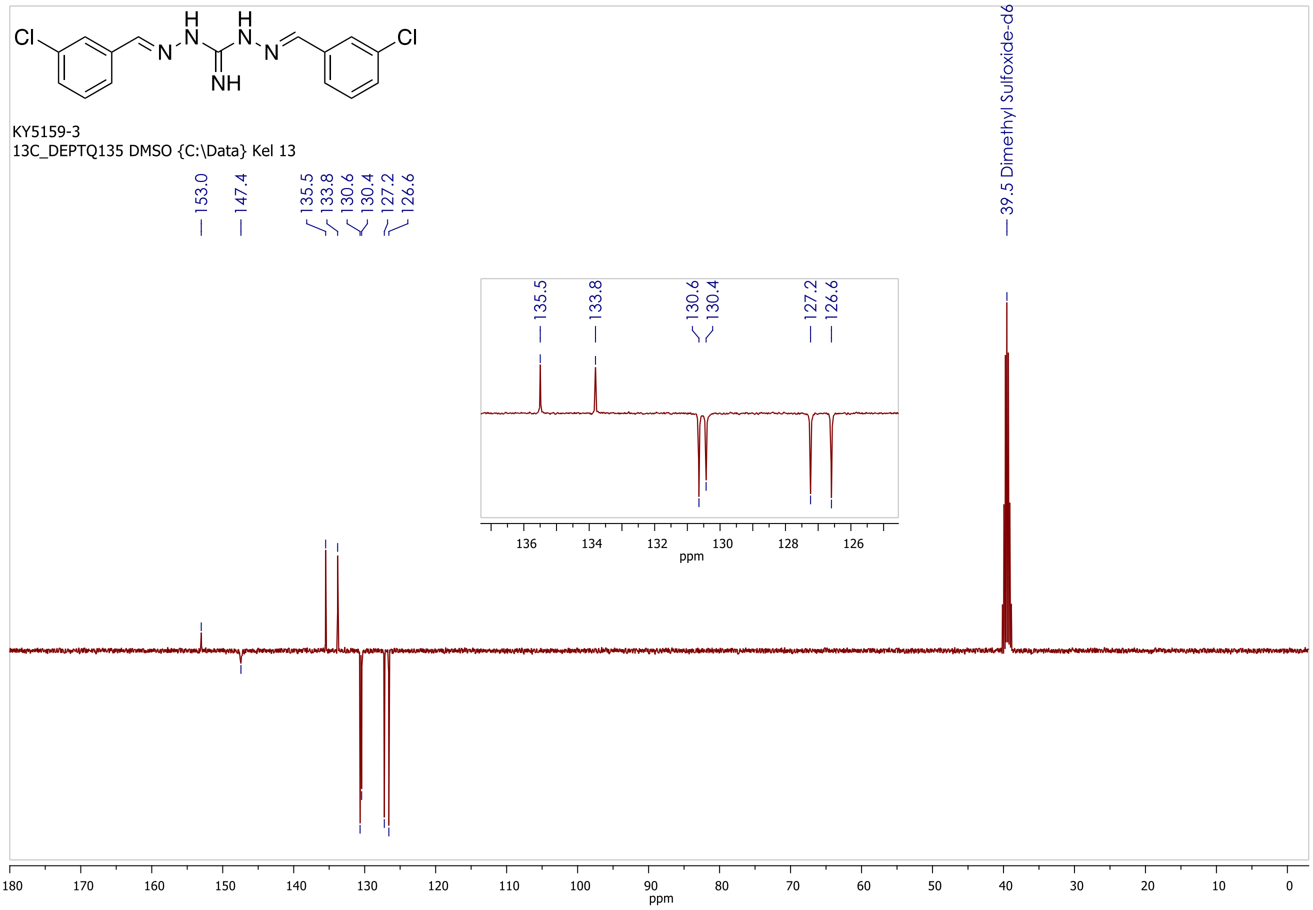

2,2'-Bis[(3-bromophenyl)methylene]carbonimidic dihydrazide hydrochloride (**9**)

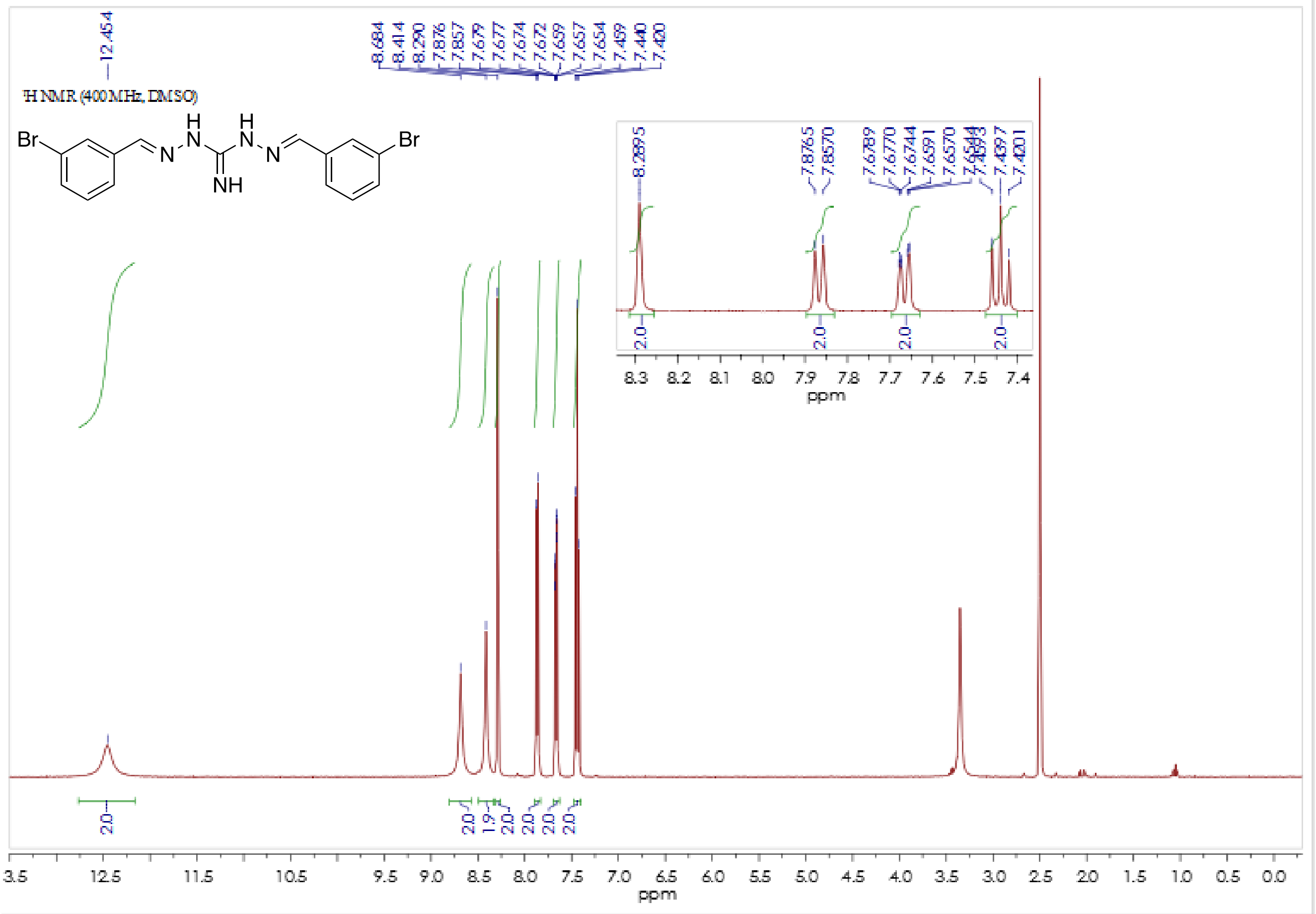

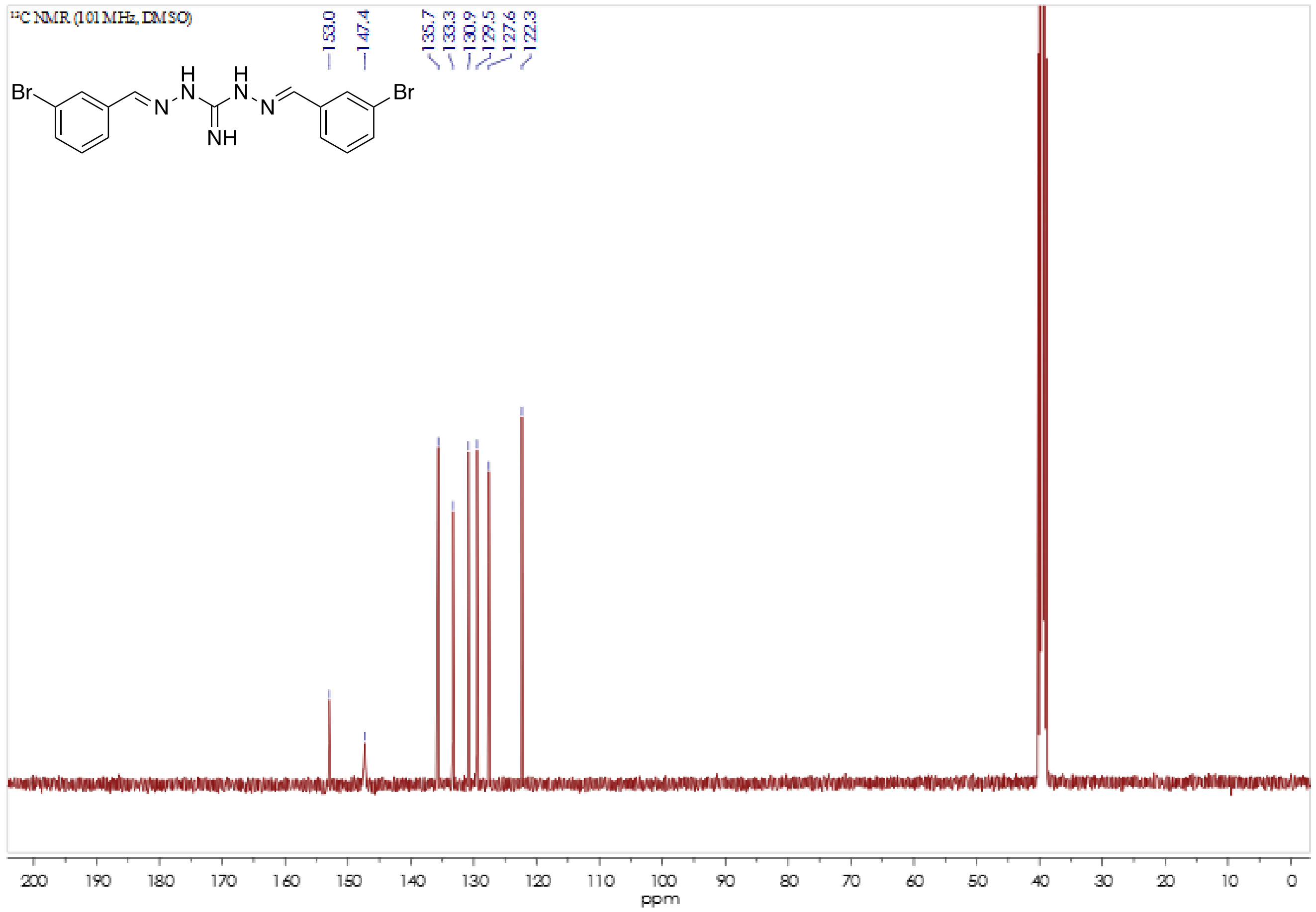

2,2'-Bis[(3-fluorophenyl)methylene]carbonimidic dihydrazide hydrochloride (**10**)

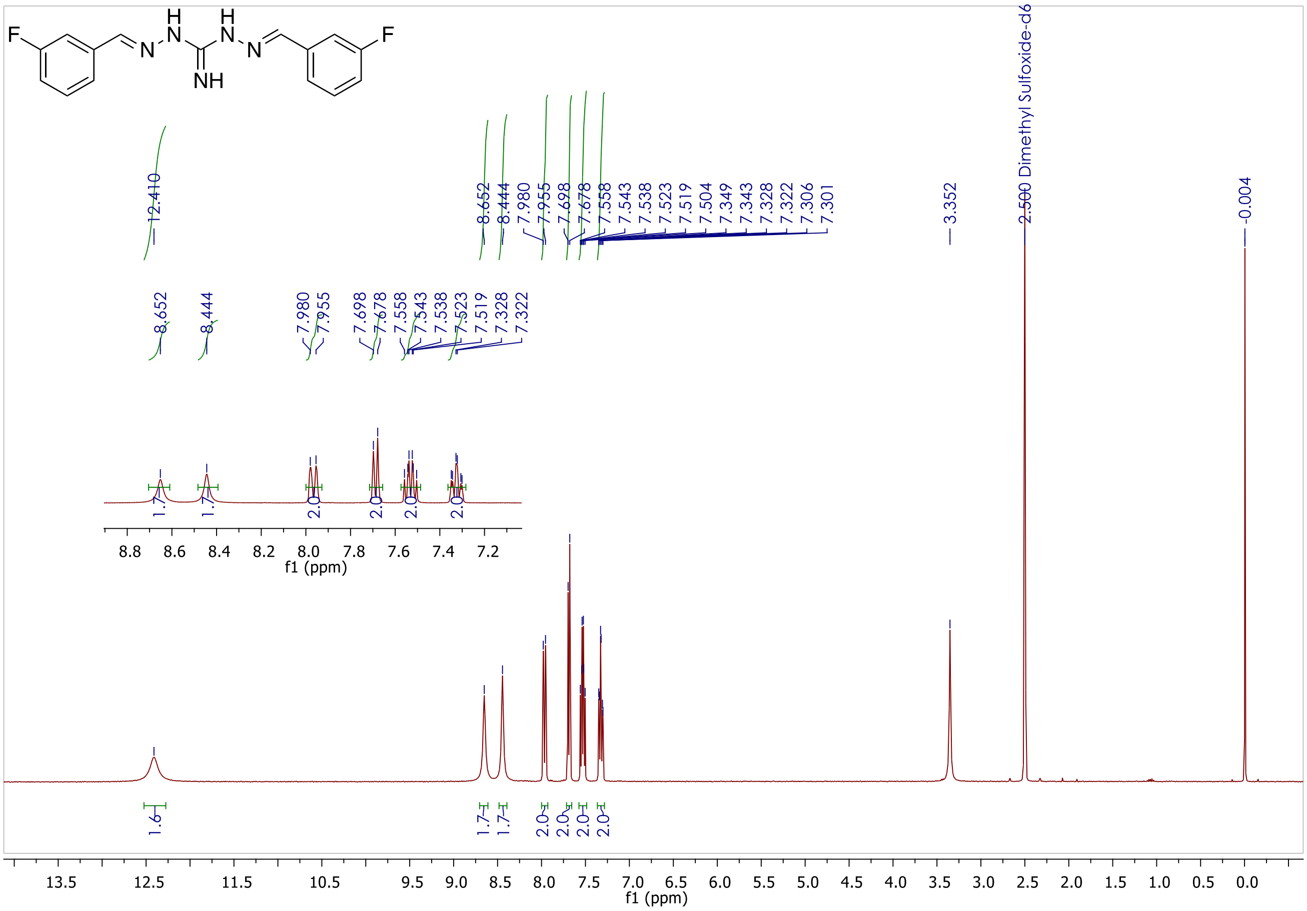

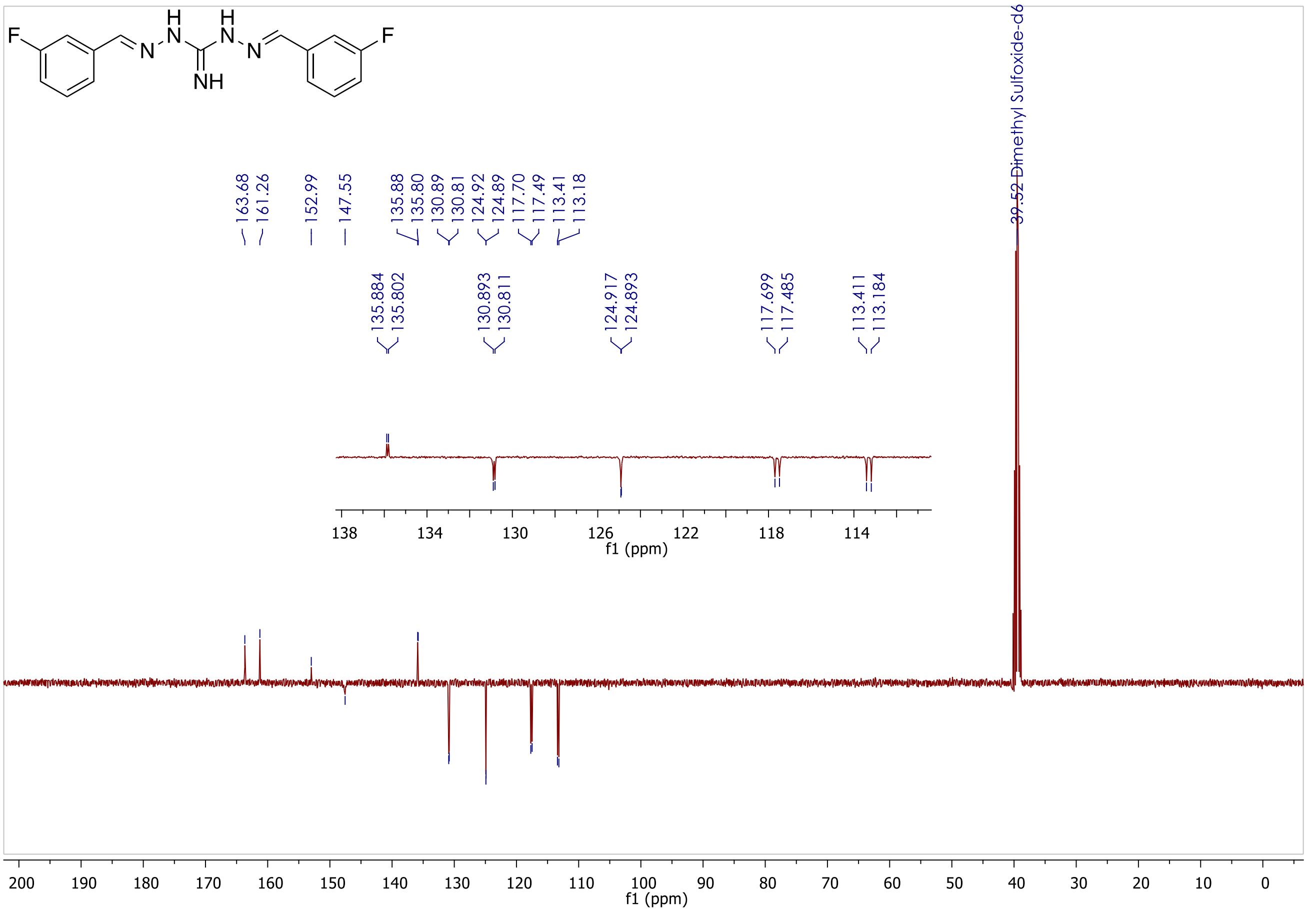

2,2'-Bis[(2-bromophenyl)methylene]carbonimidic dihydrazide hydrochloride (**11**)

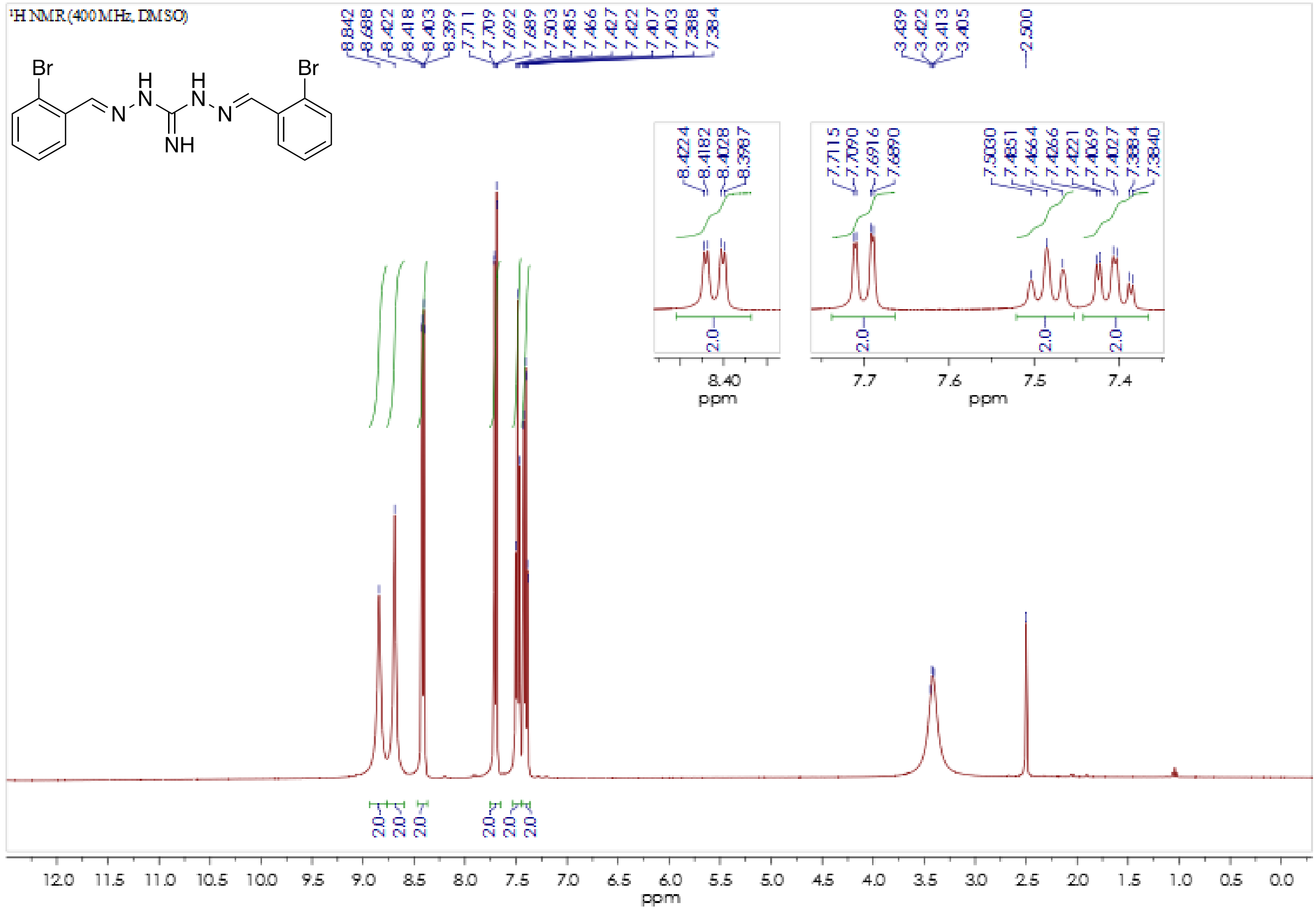

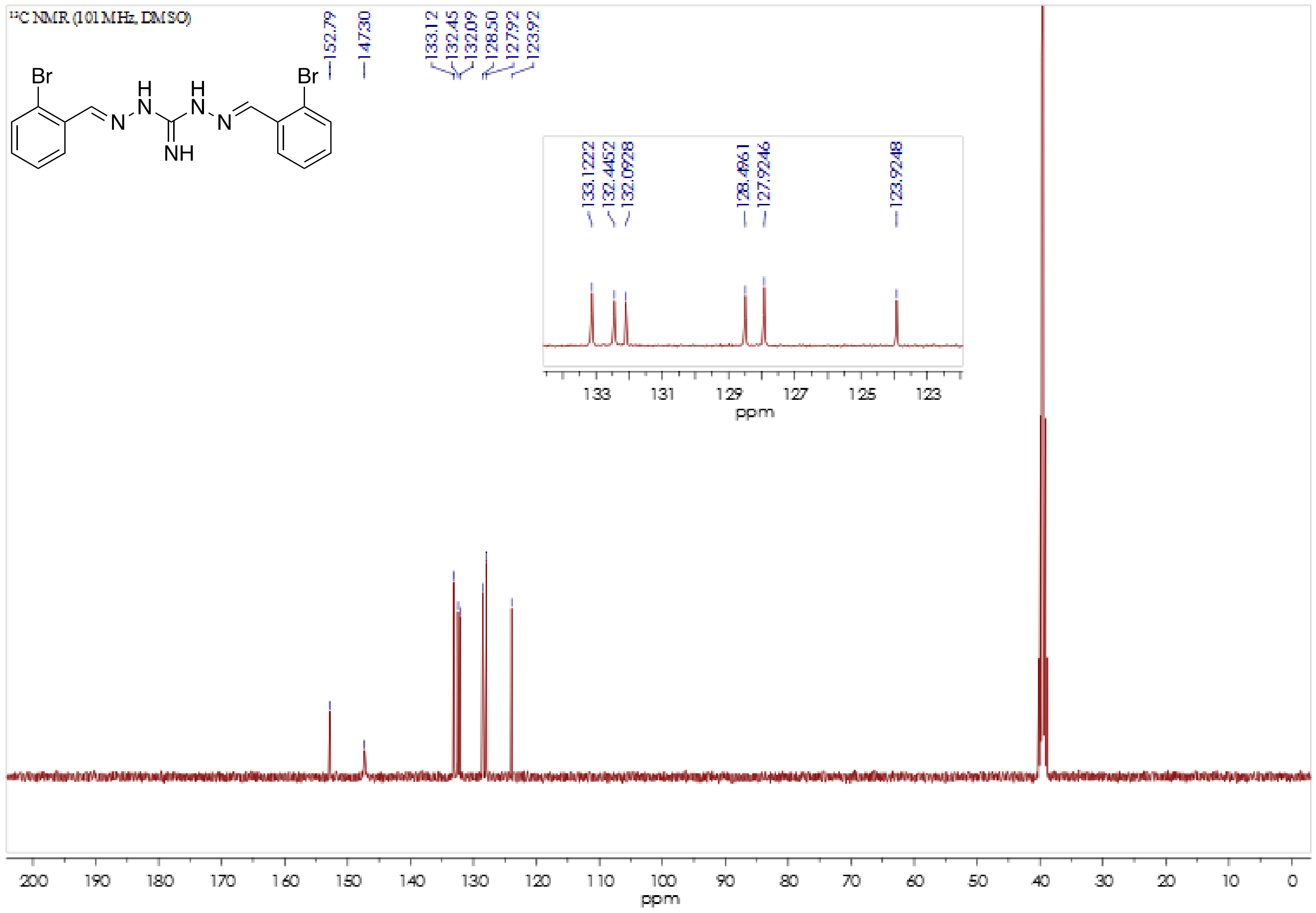

2,2'-Bis[(2-chlorophenyl)methylene]carbonimidic dihydrazide hydrochloride (**12**)

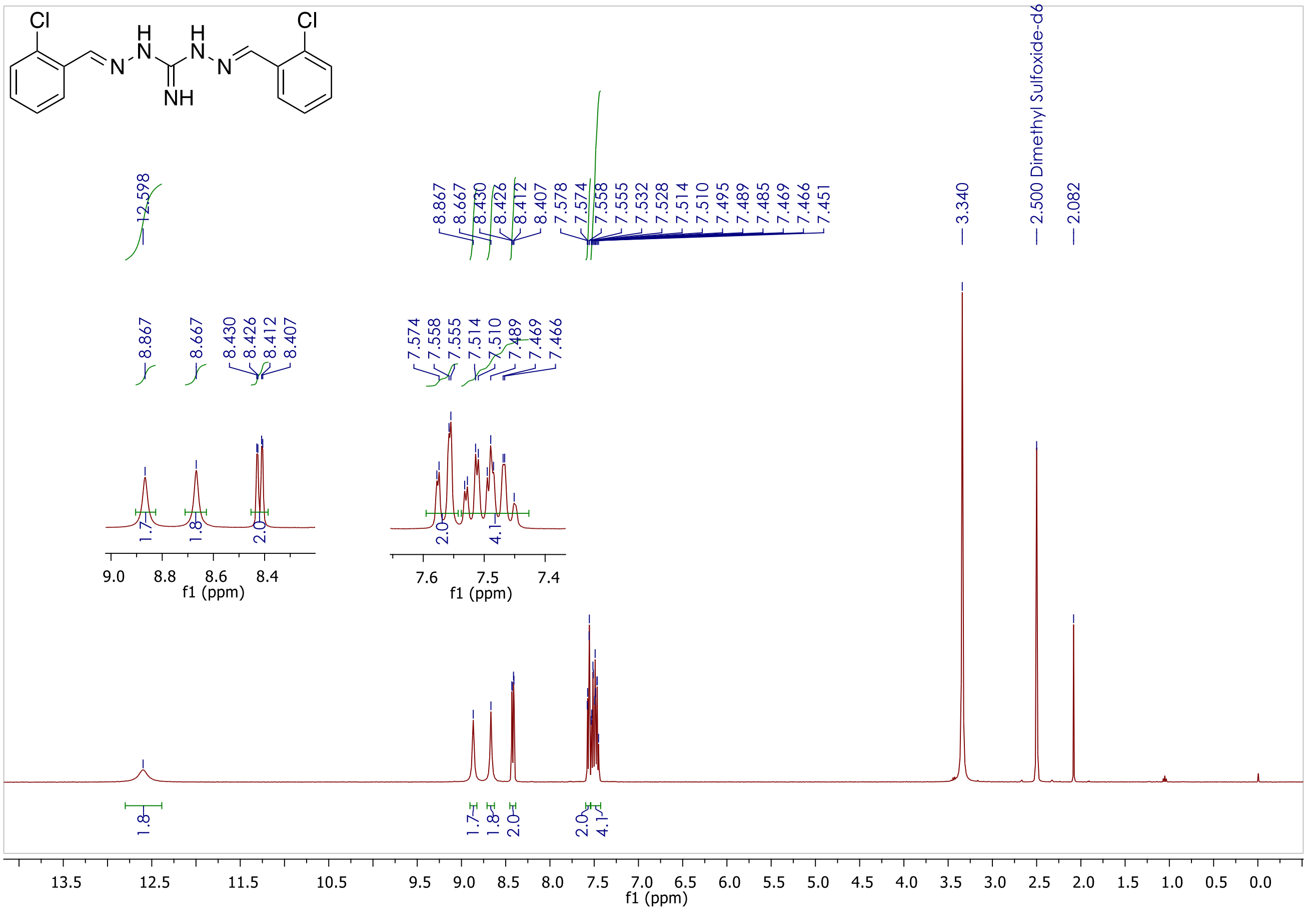

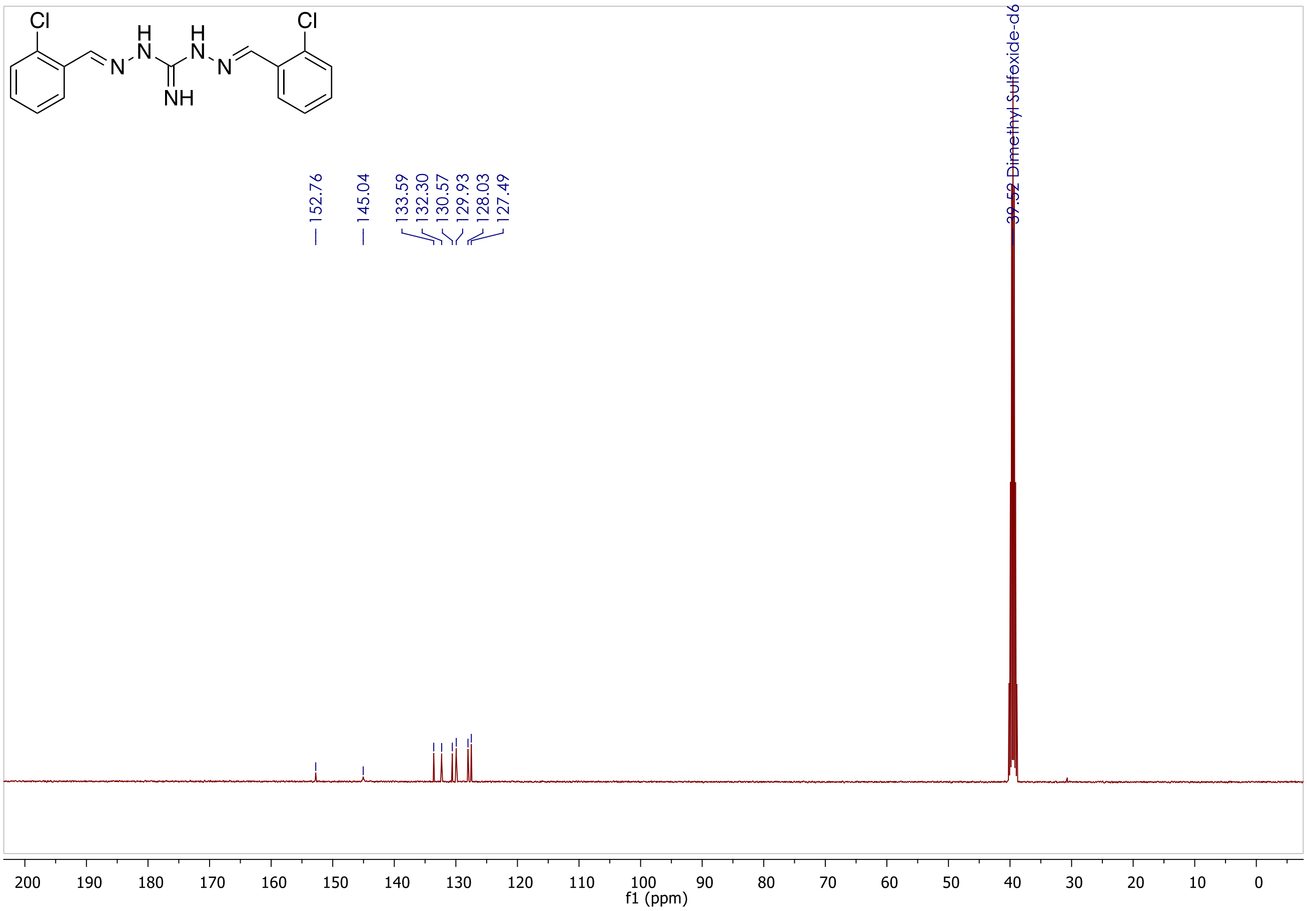

2,2'-Bis[(2-fluorophenyl)methylene]carbonimidic dihydrazide hydrochloride (**13**)

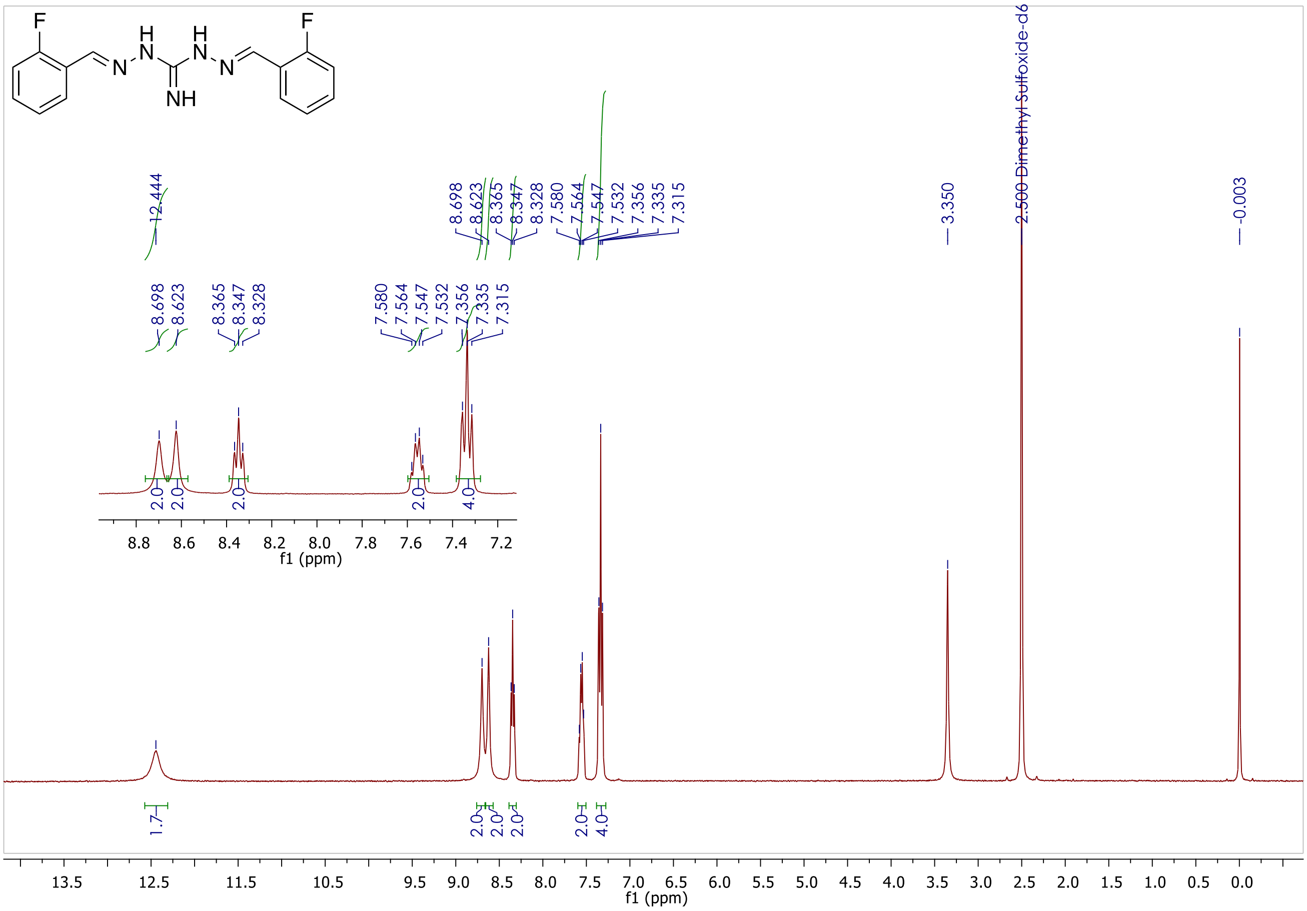

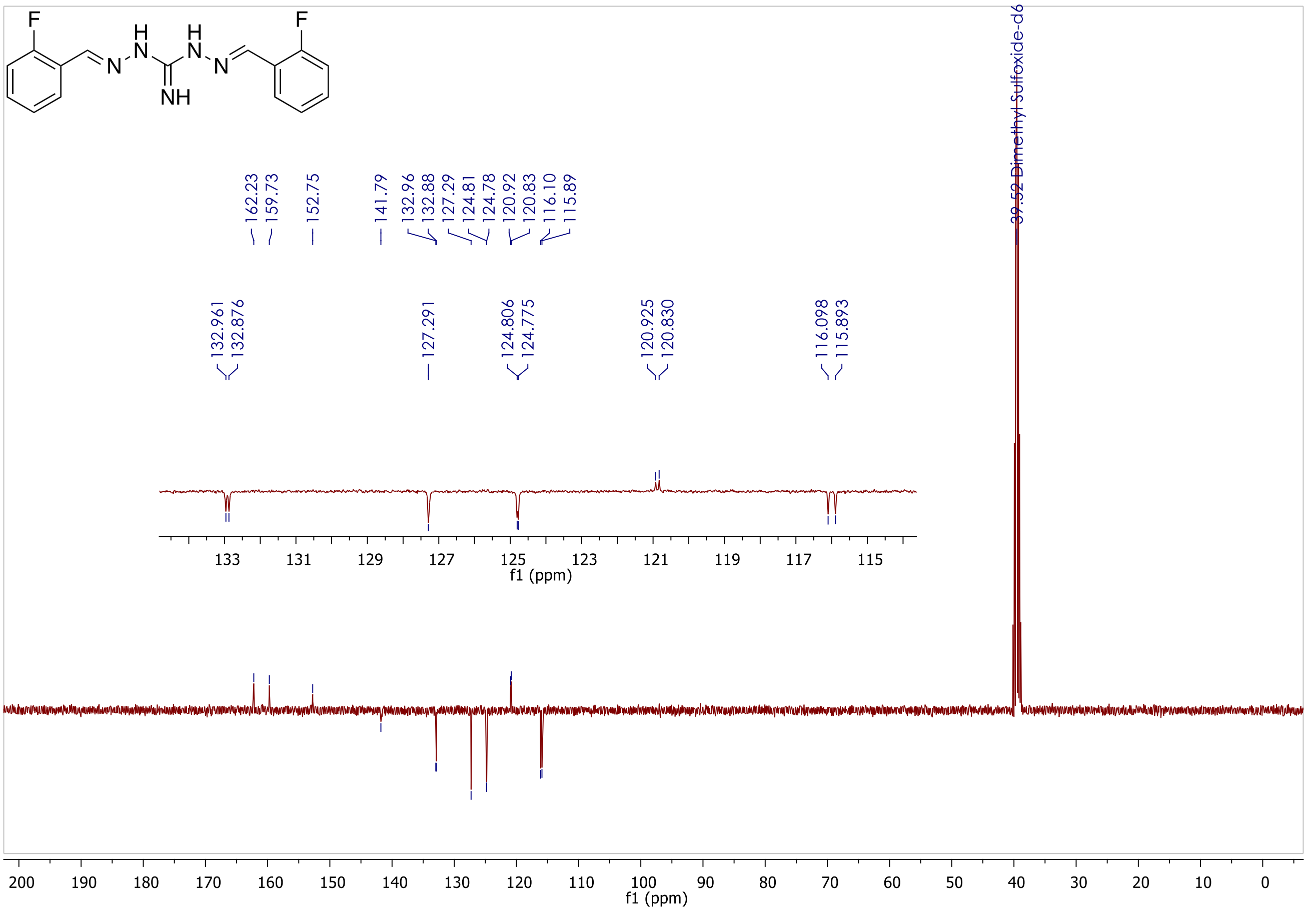

2,2'-Bis[(4-methylphenyl)methylene]carbonimidic dihydrazide hydrochloride (**14**)

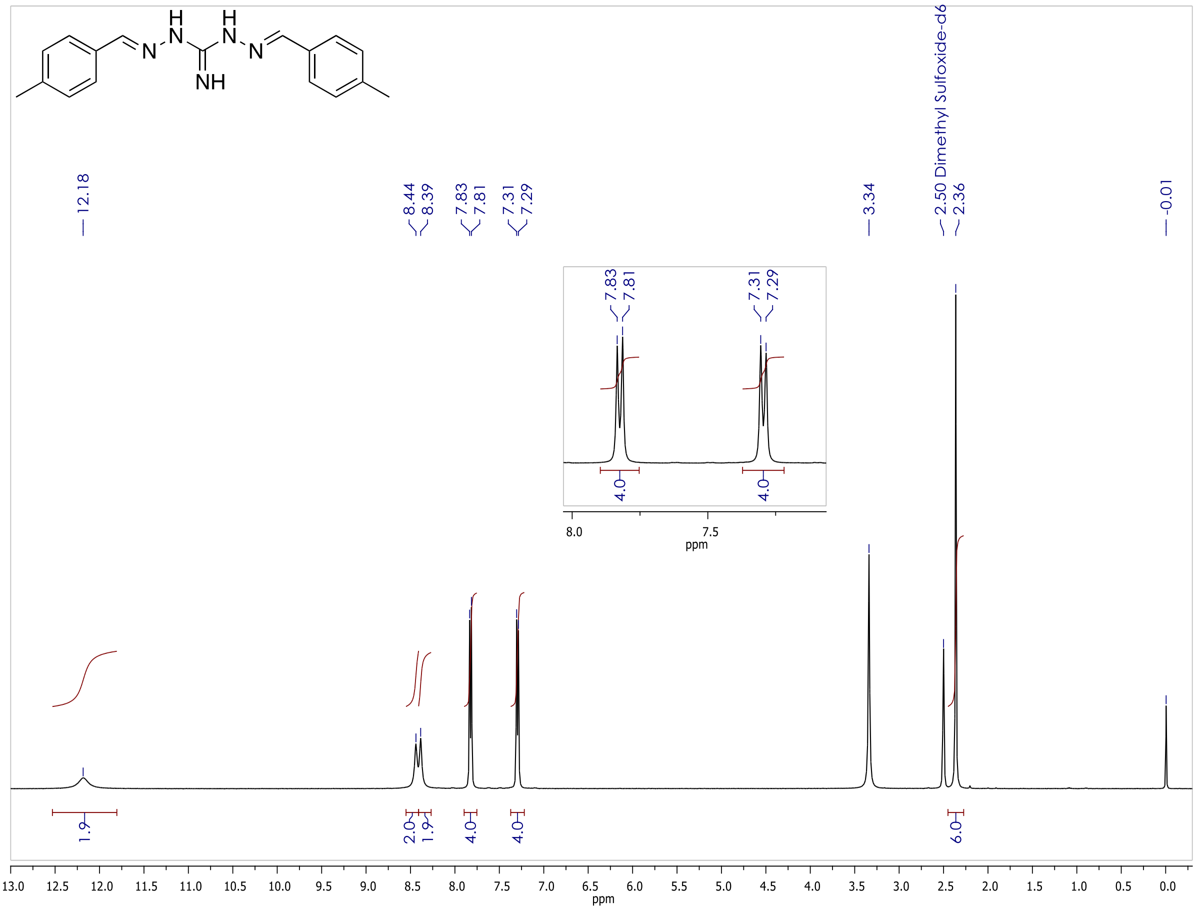

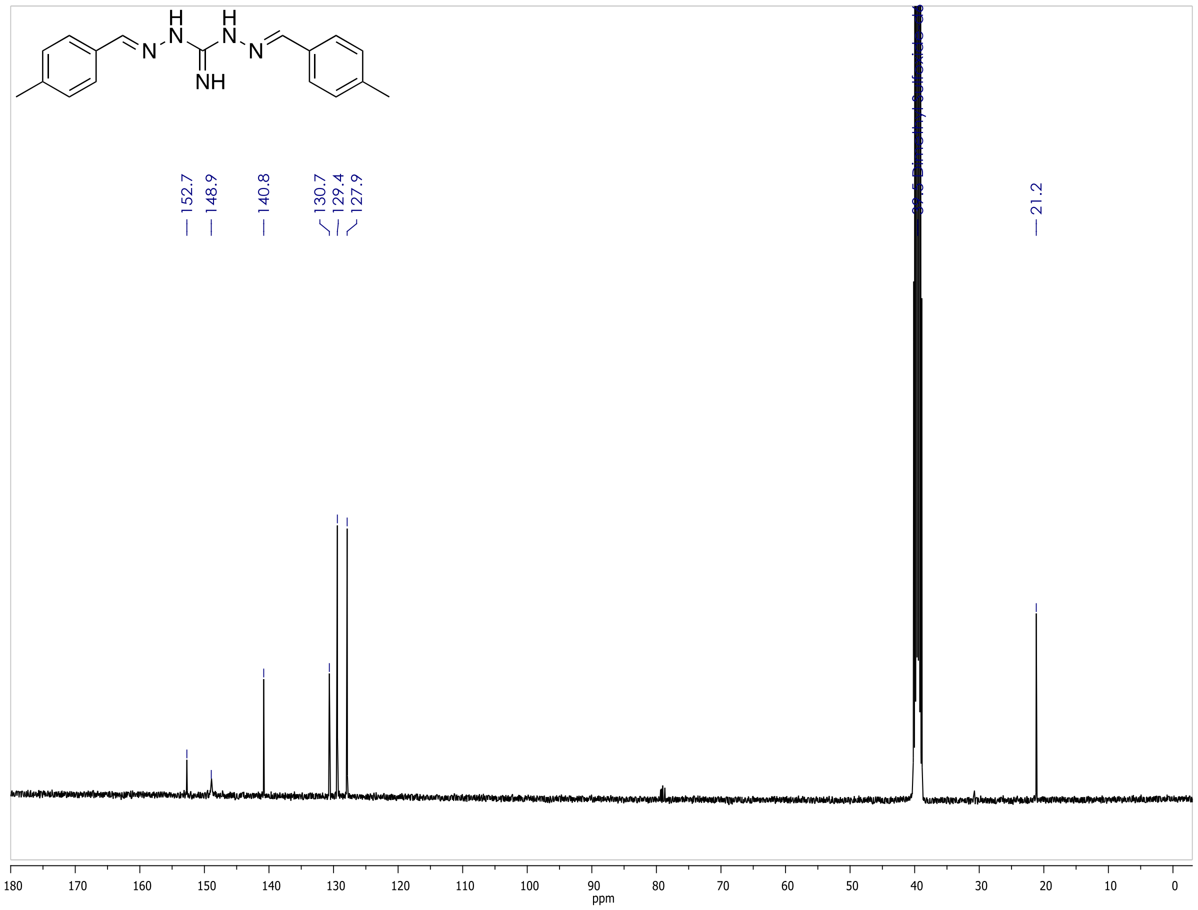

2,2'-Bis[(3-methylphenyl)methylene]carbonimidic dihydrazide hydrochloride (**15**)

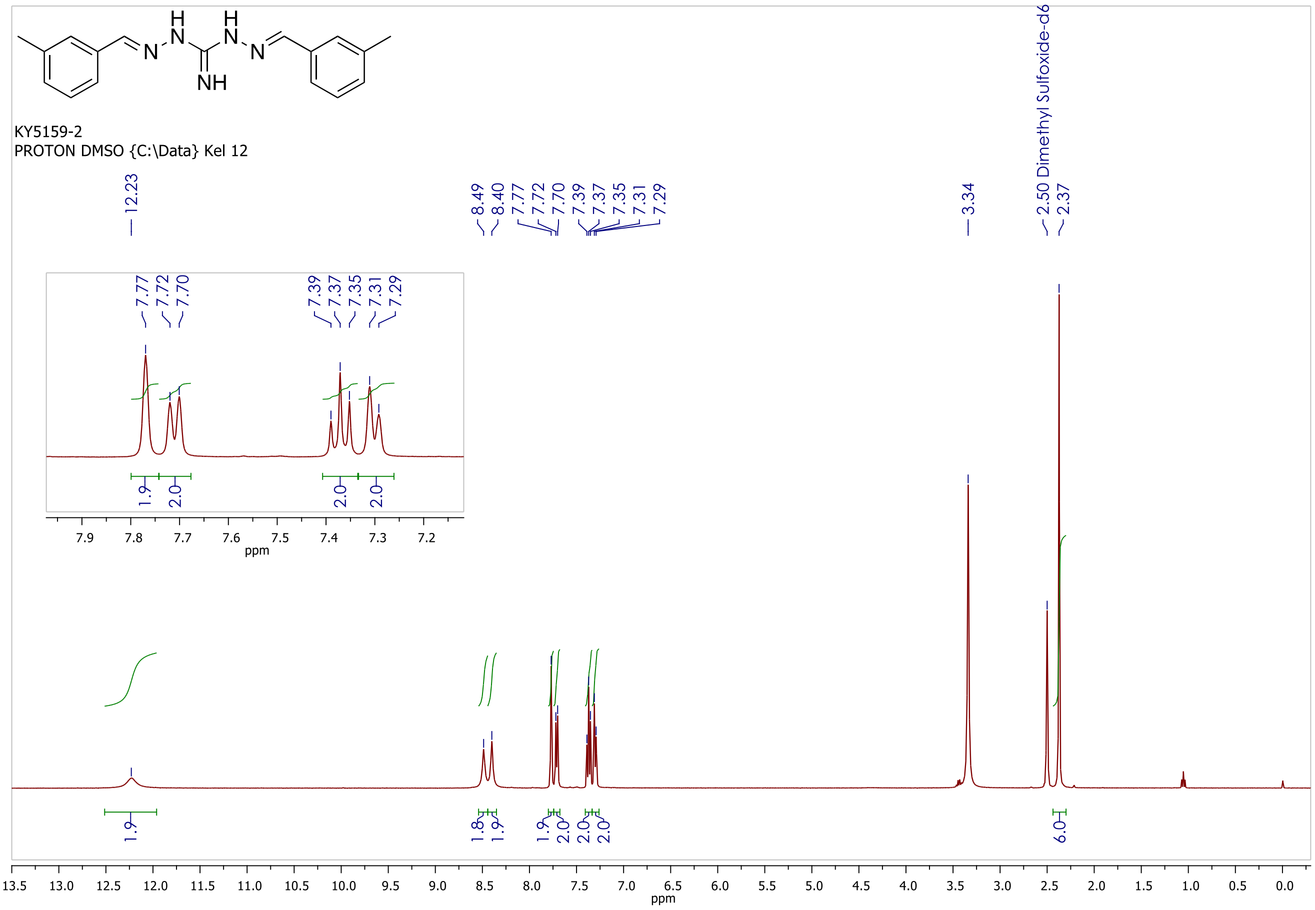

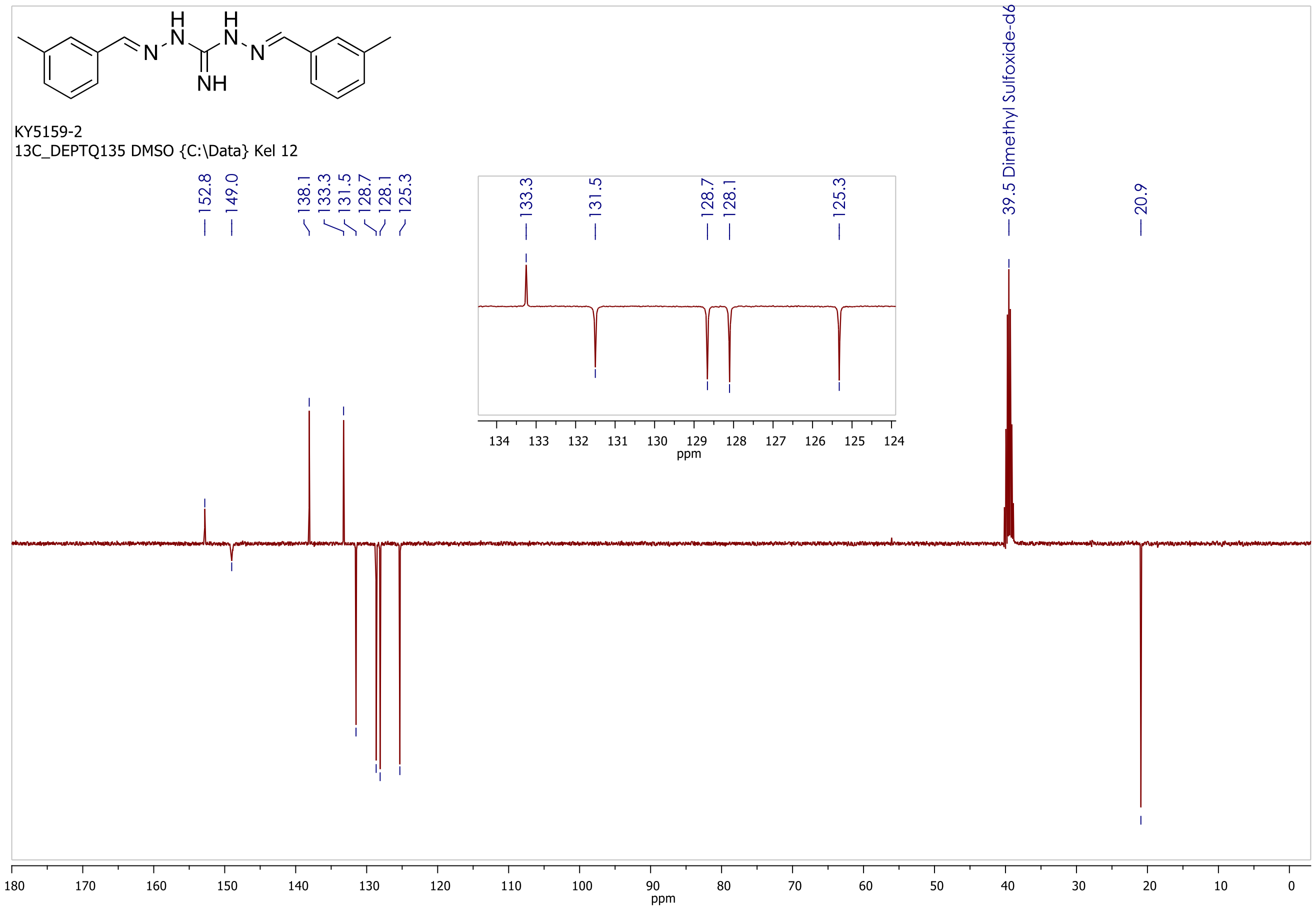

2,2'-Bis[(2-methylphenyl)methylene]carbonimidic dihydrazide hydrochloride (**16**)

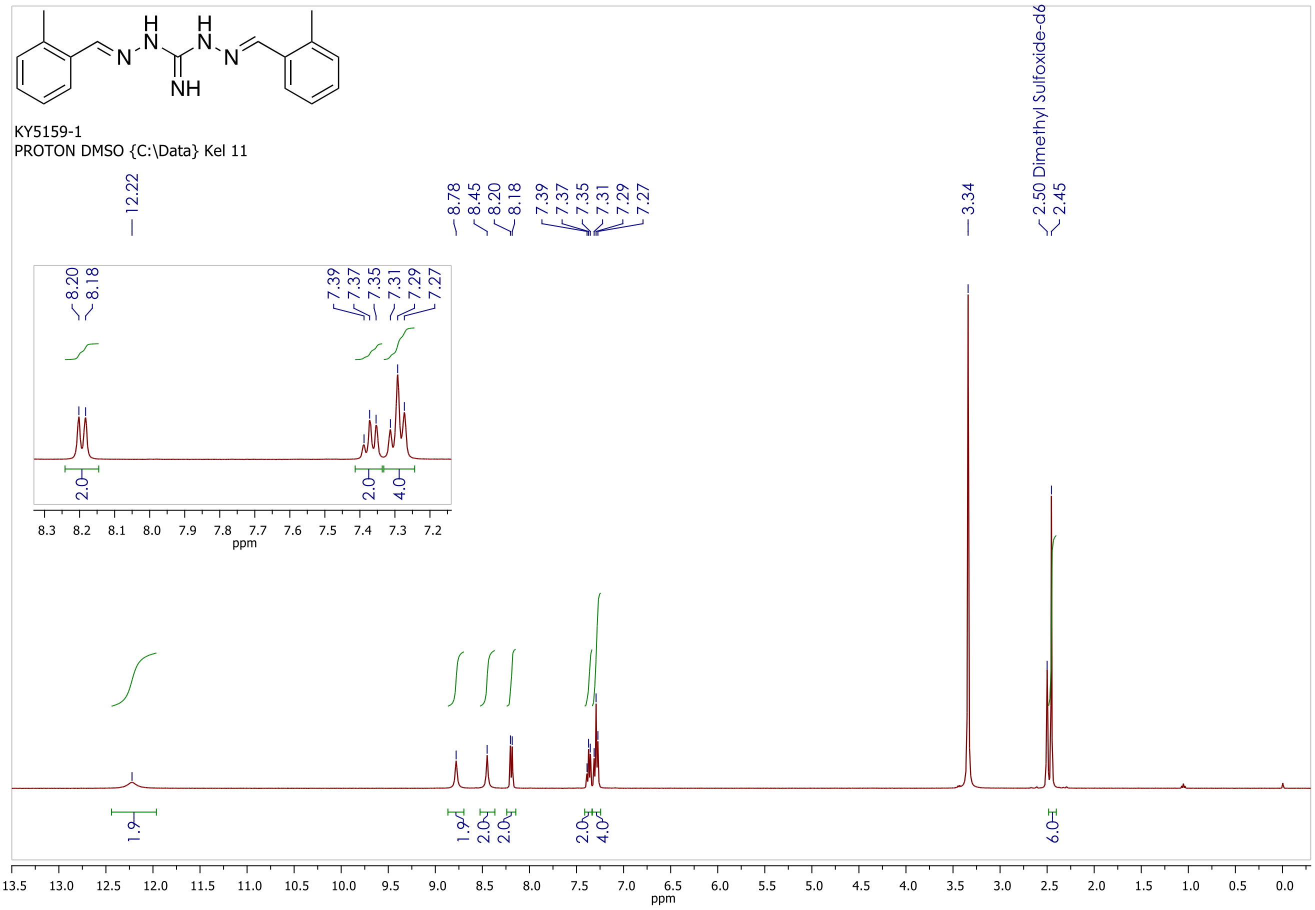

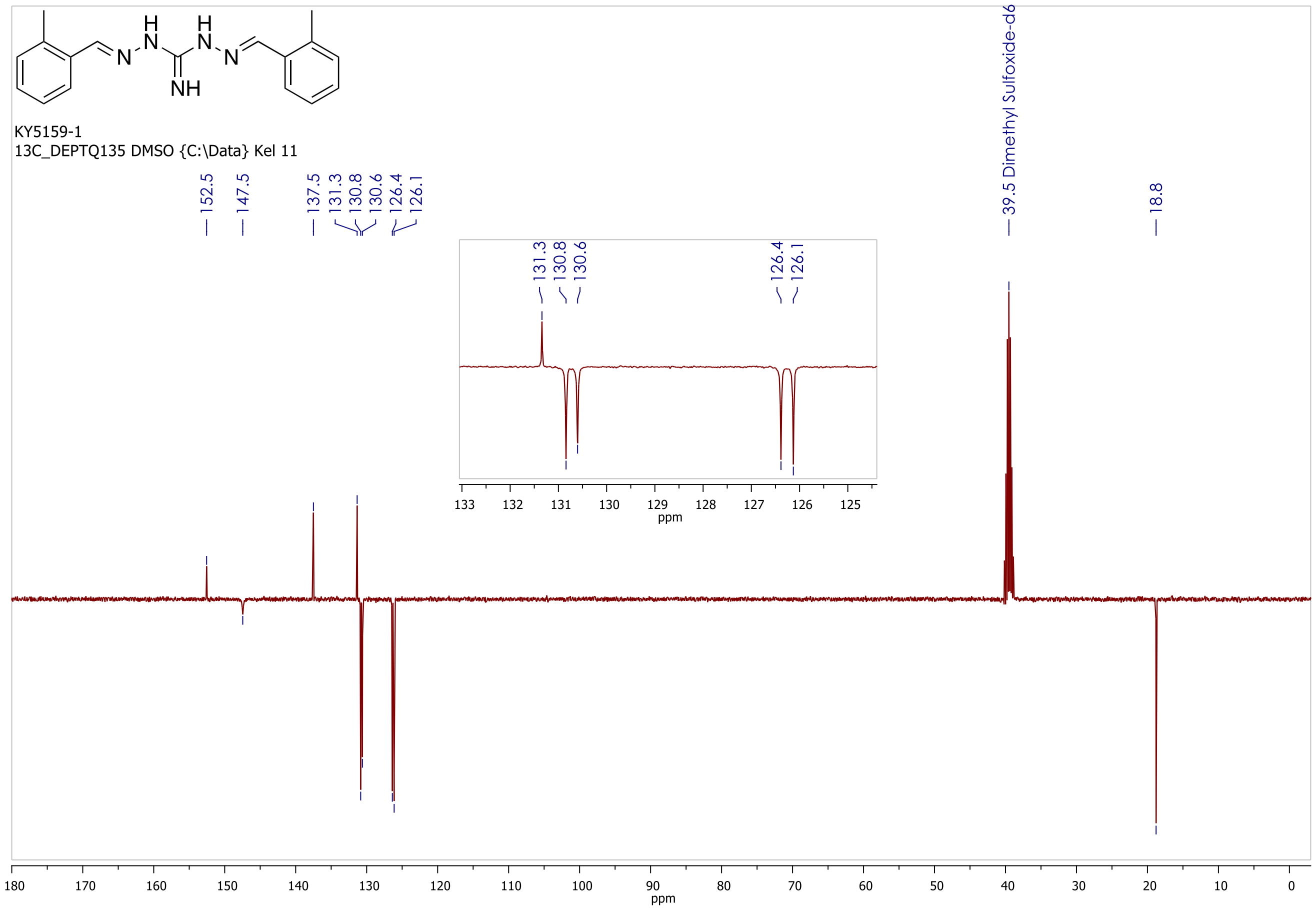

2,2'-Bis([1,1'-biphenyl]-4-ylmethylene)carbonimidic dihydrazide hydrochloride (**17**)

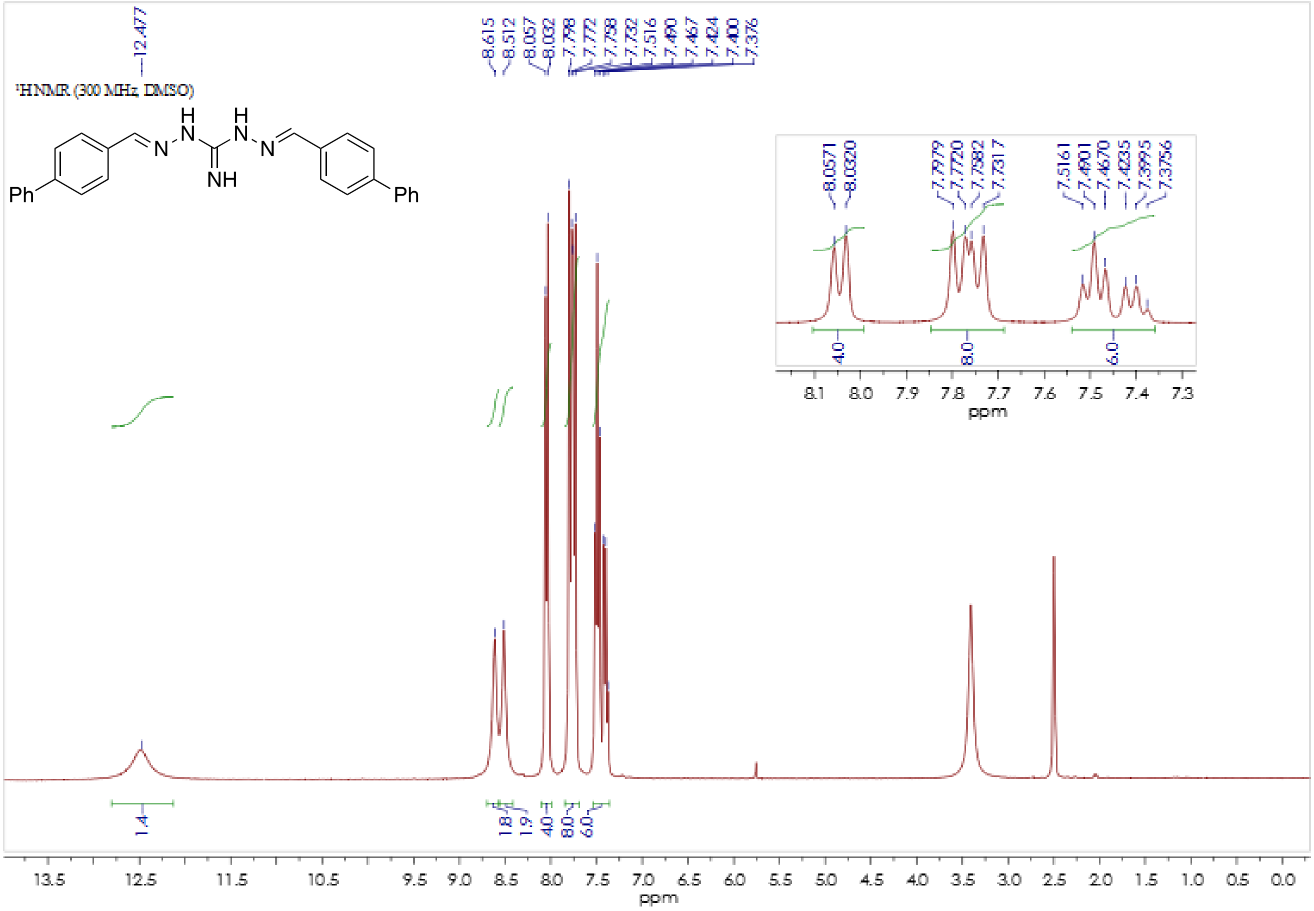

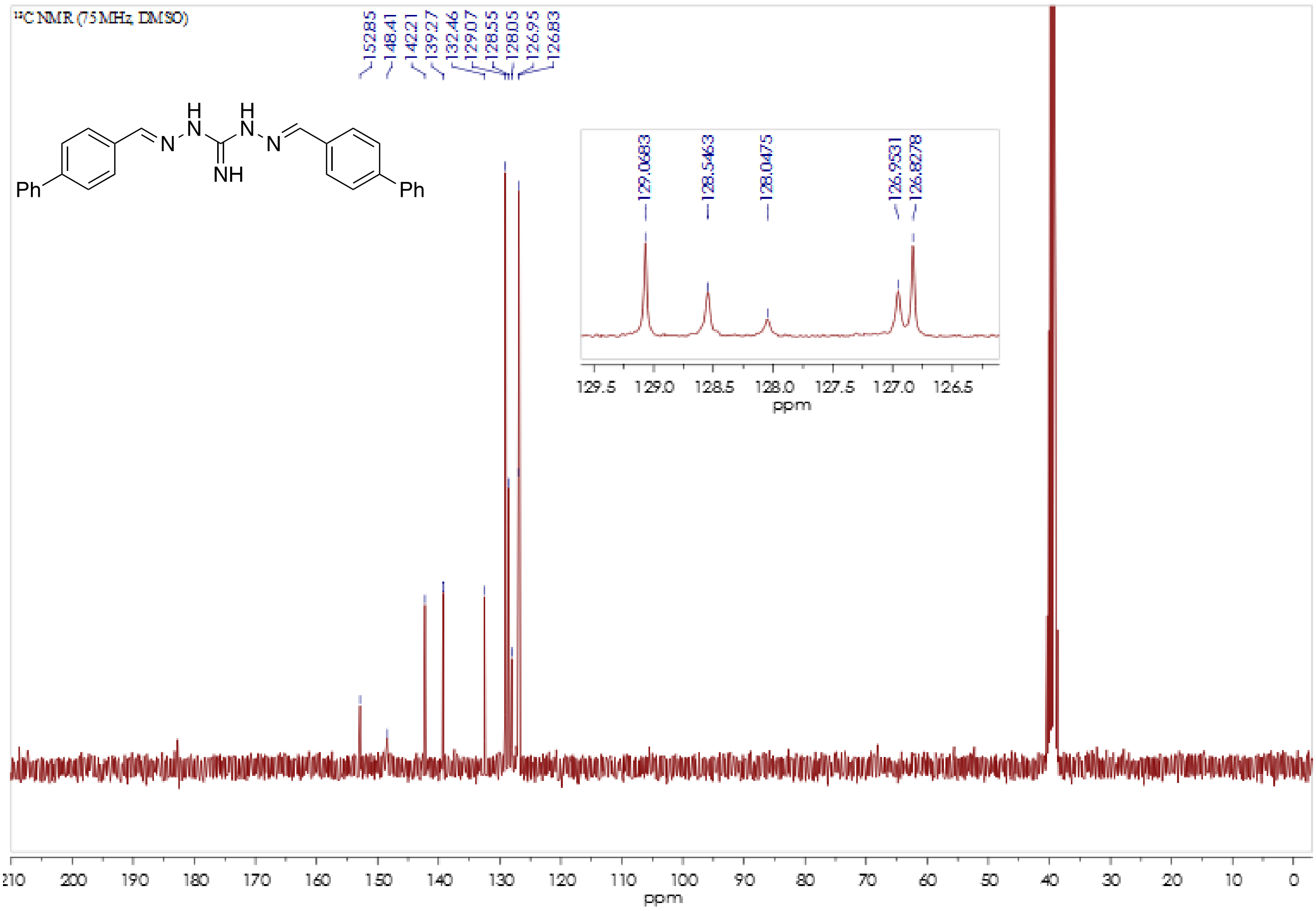

2,2'-Bis([1,1'-biphenyl]-2-ylmethylene)carbonimidic dihydrazide hydrochloride (**18**)

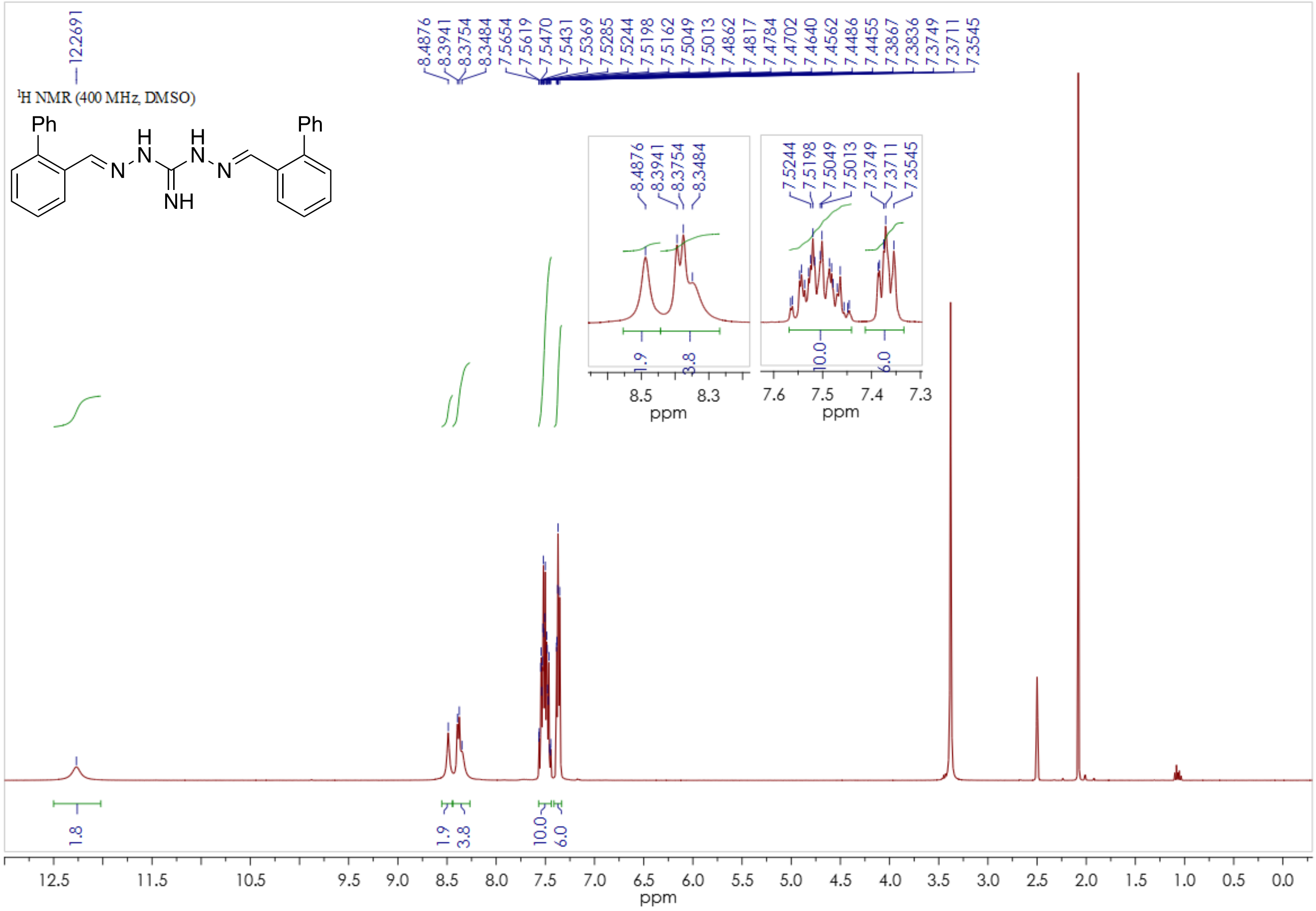

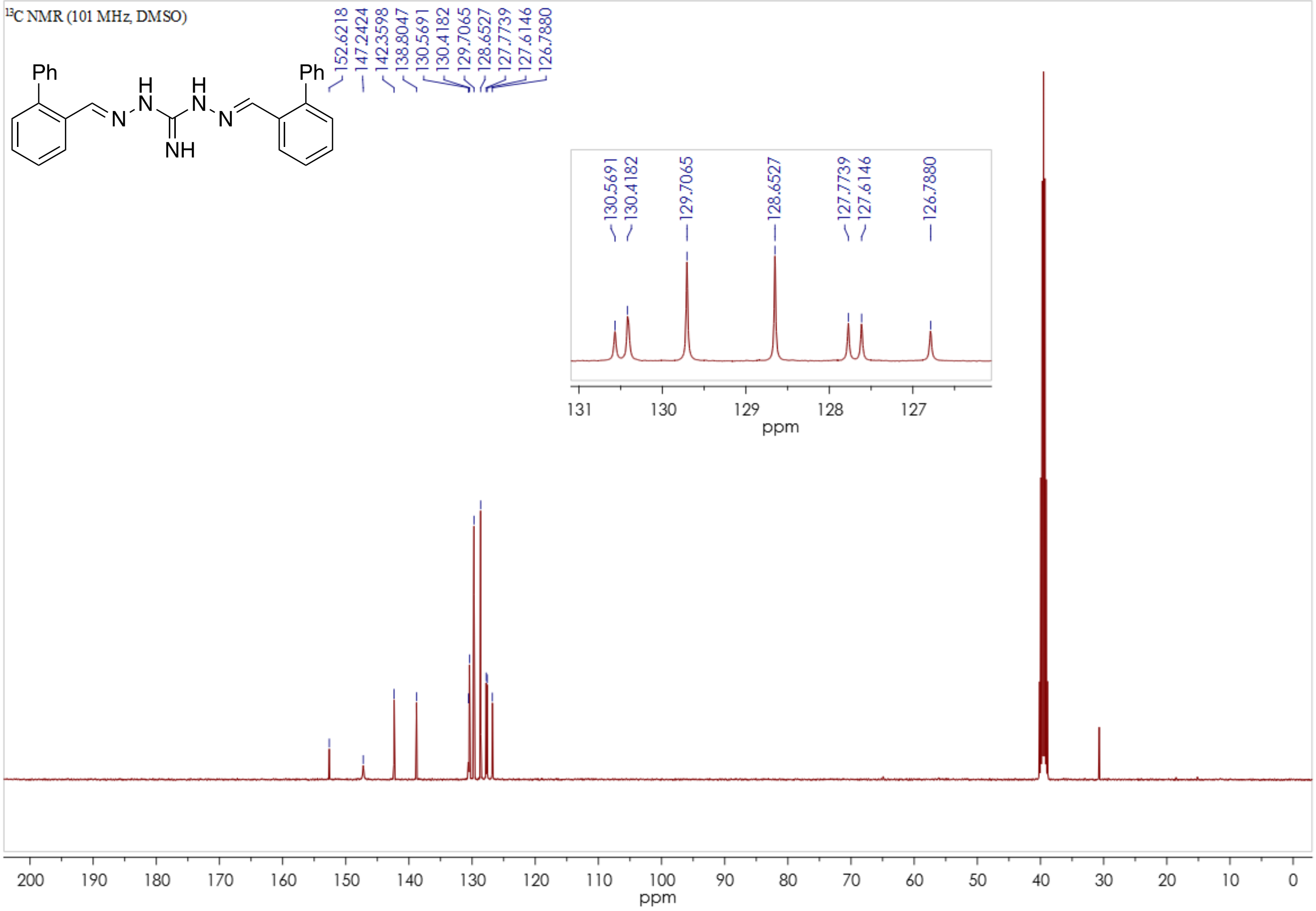

2,2'-Bis[(4-propylphenyl)methylene]carbonimidic dihydrazide hydrochloride (**19**)

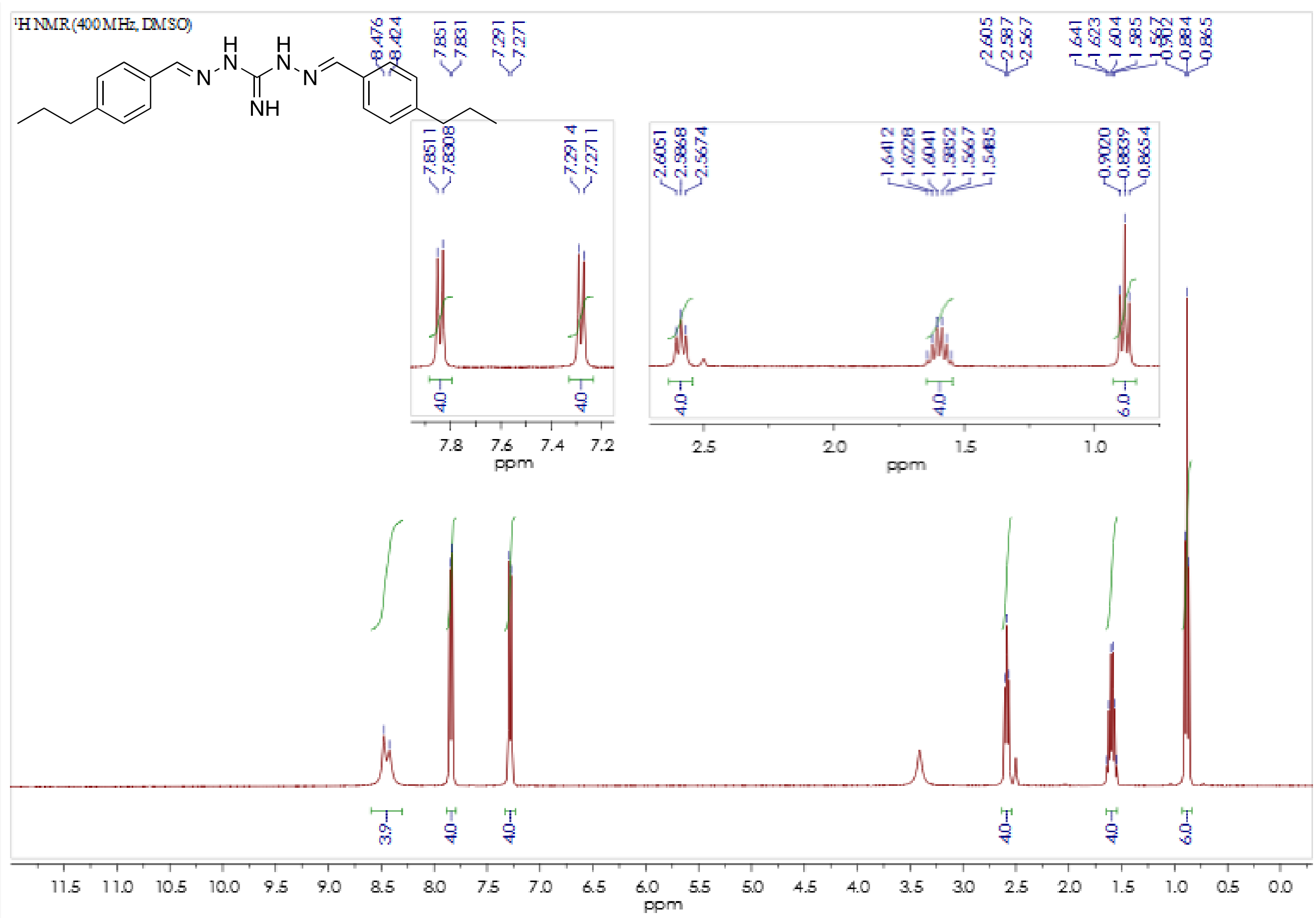

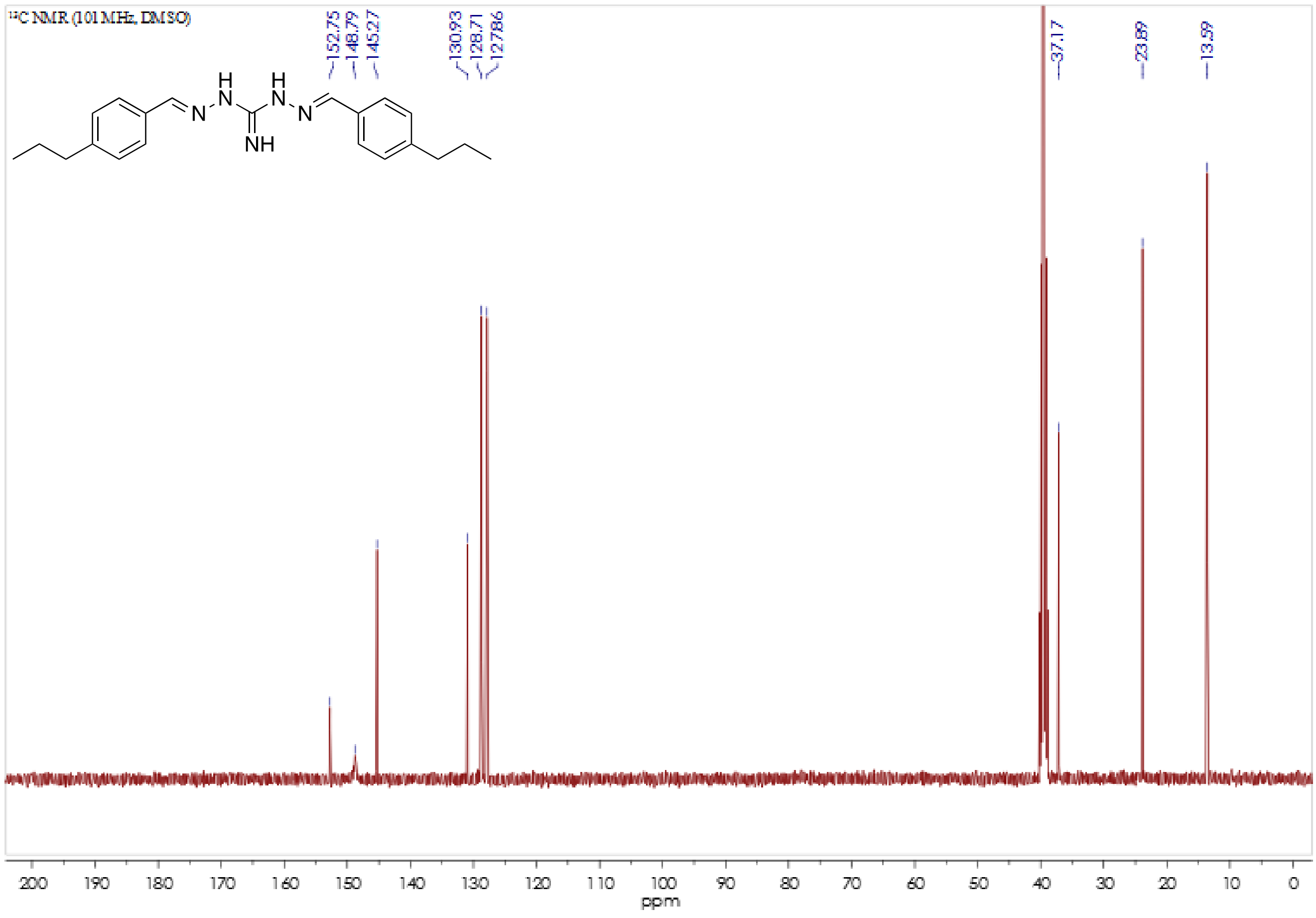

2,2'-Bis[(4-butylphenyl)methylene]carbonimidic dihydrazide hydrochloride (**20**)

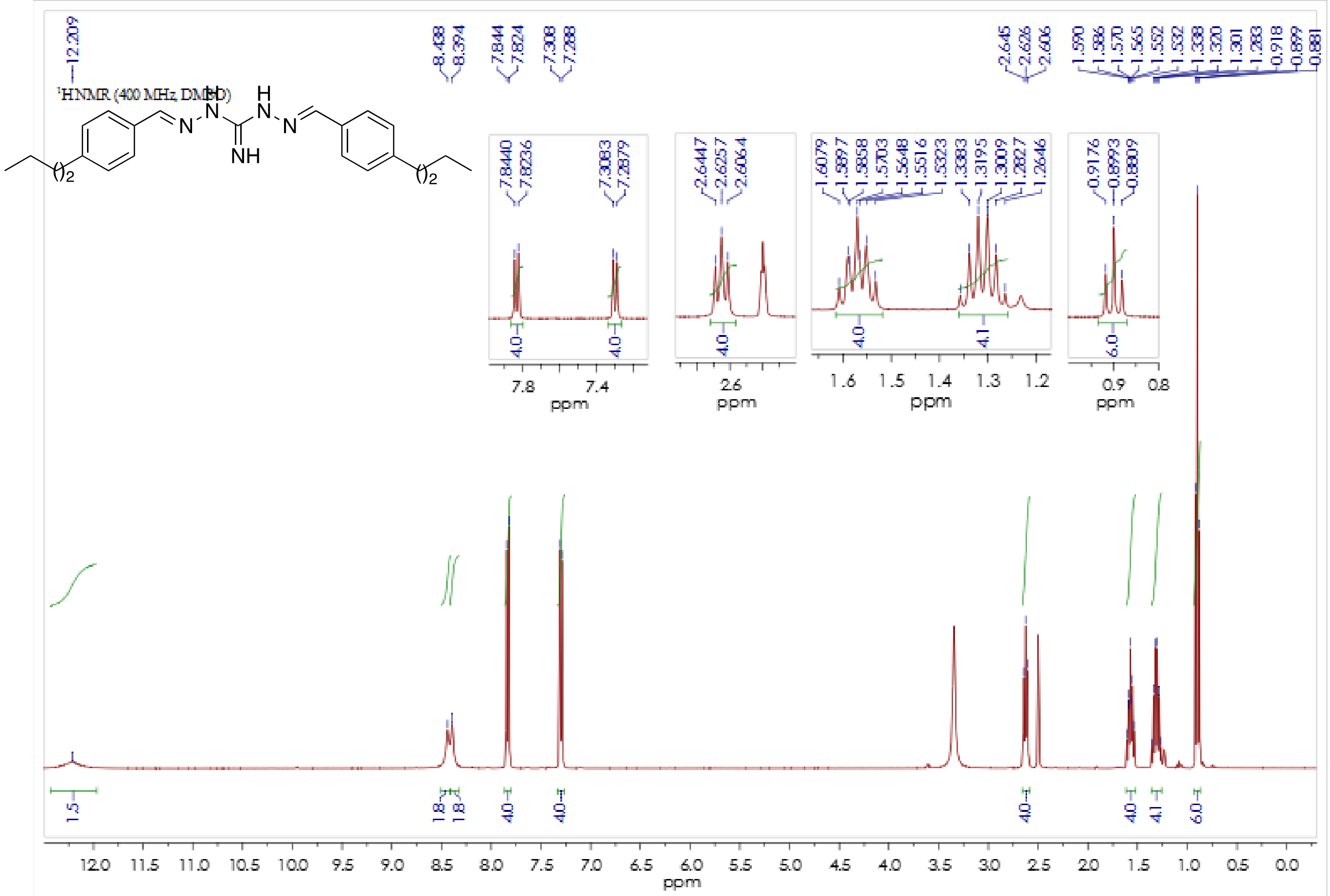

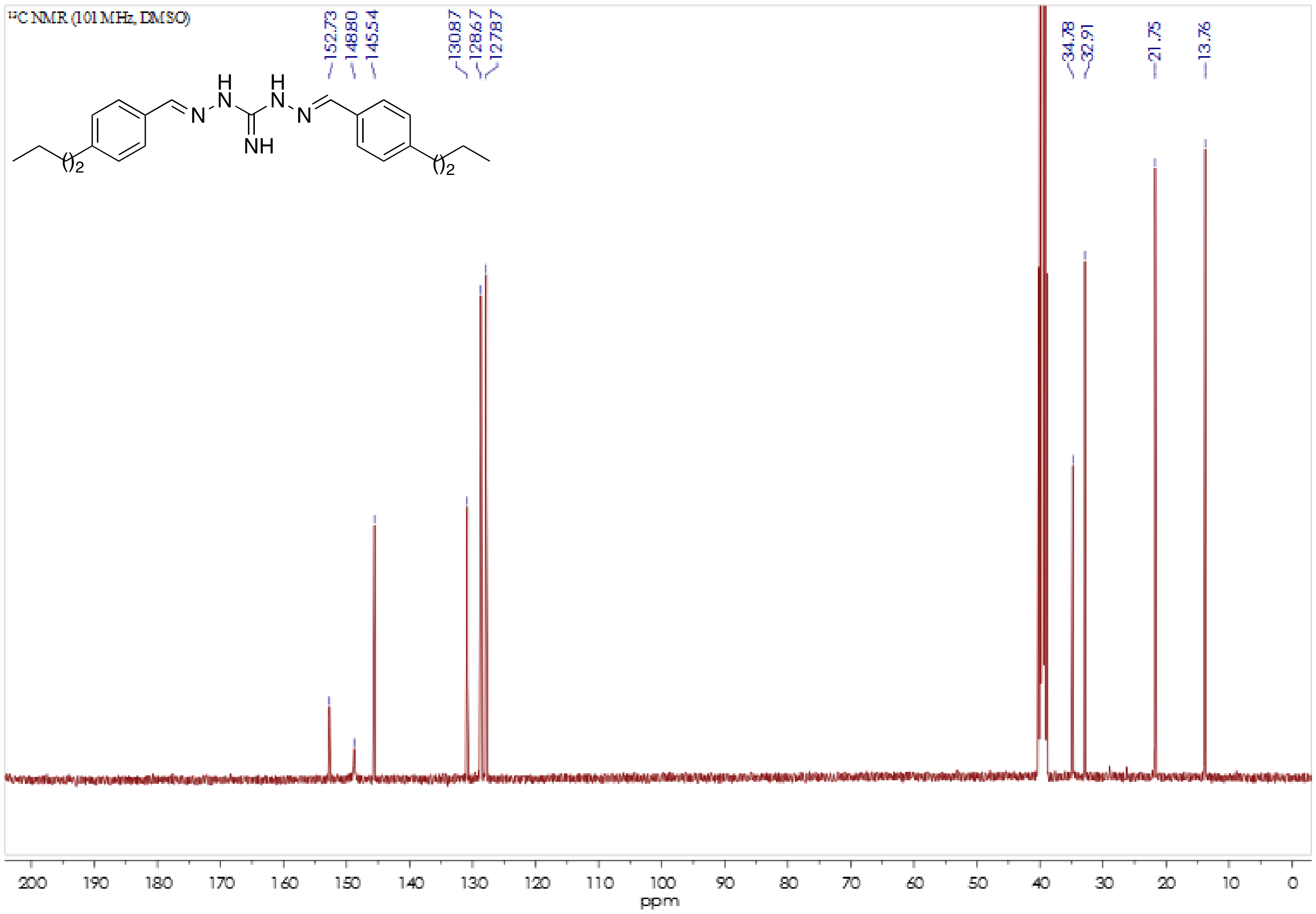

2,2'-Bis{[4-(1-methylethyl)phenyl]methylene}carbonimidic dihydrazide hydrochloride (**21**)

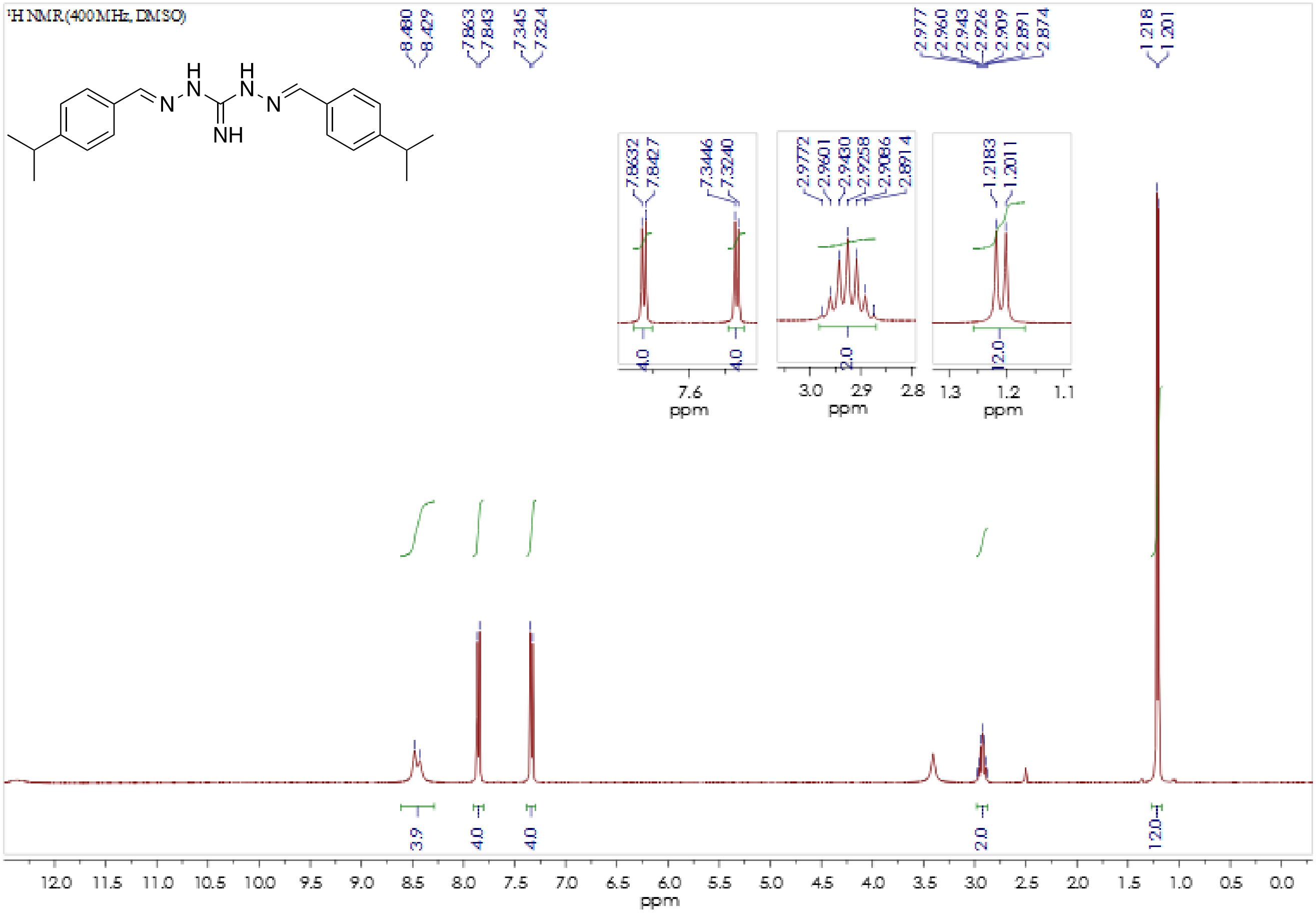

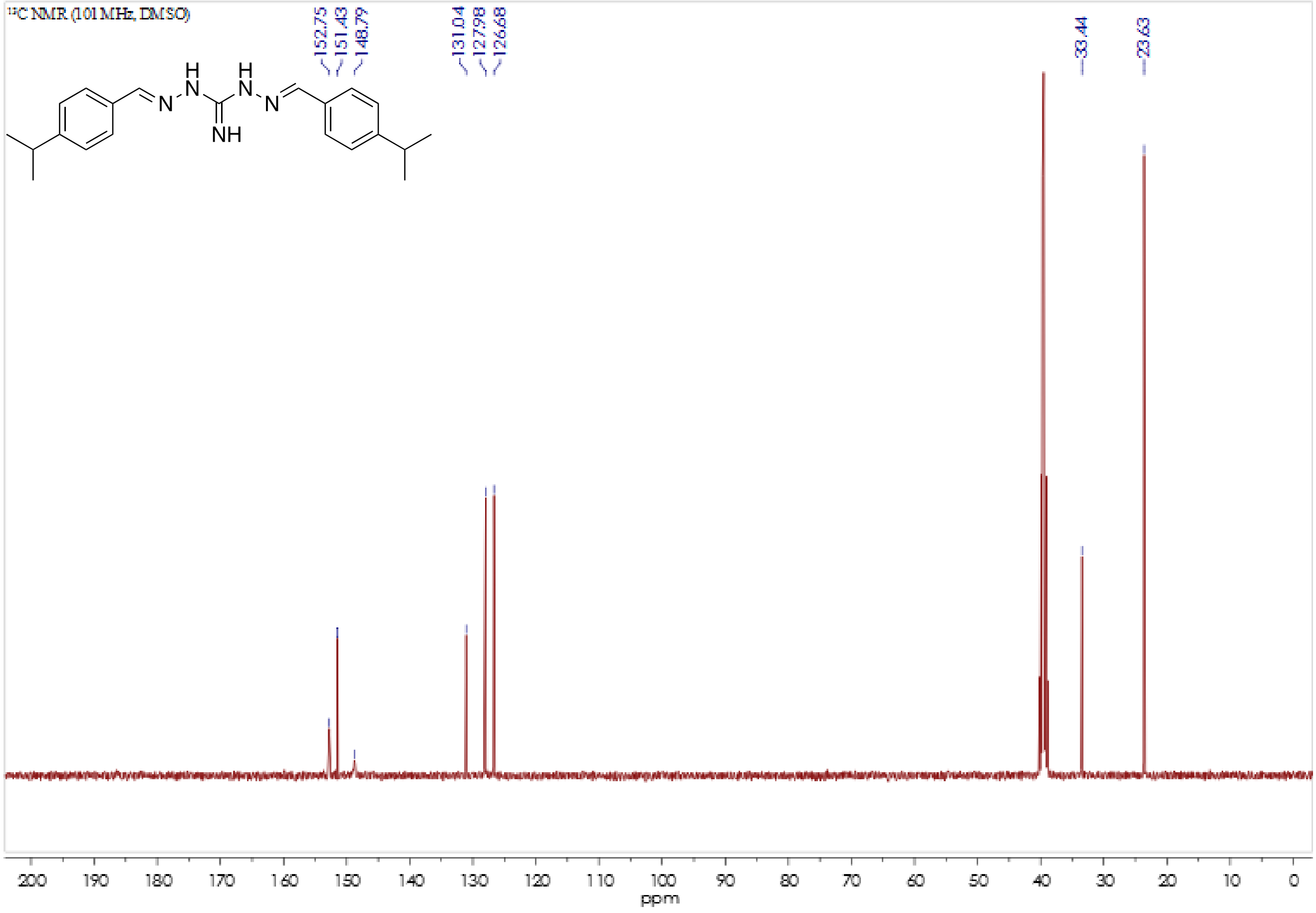

2,2'-Bis{[4-(1,1-dimethylethyl)phenyl]methylene}carbonimidic dihydrazide hydrochloride (**22**)

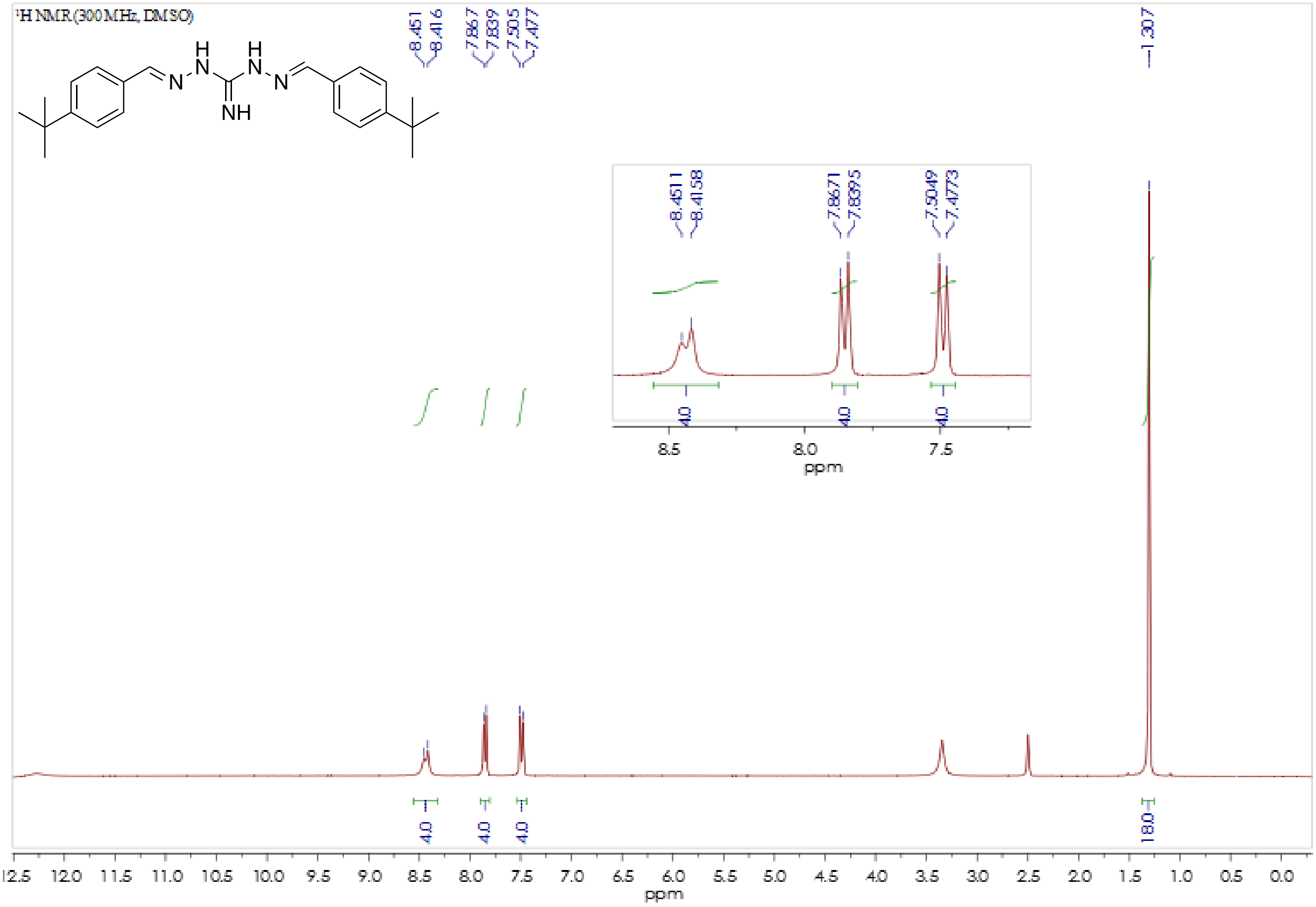

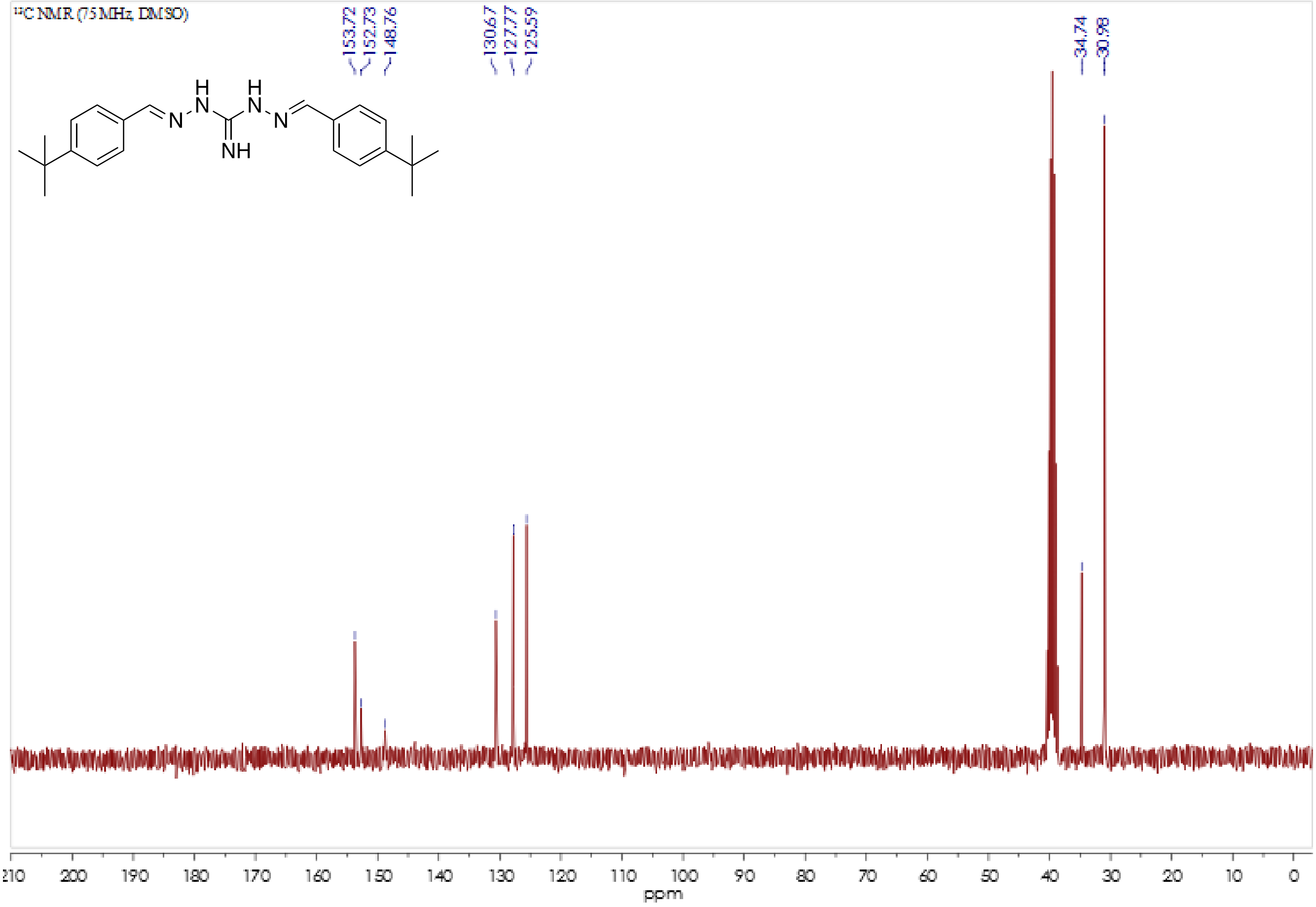

2,2'-Bis[(3-ethynylphenyl)methylene]carbonimidic dihydrazide hydrochloride (**23**)

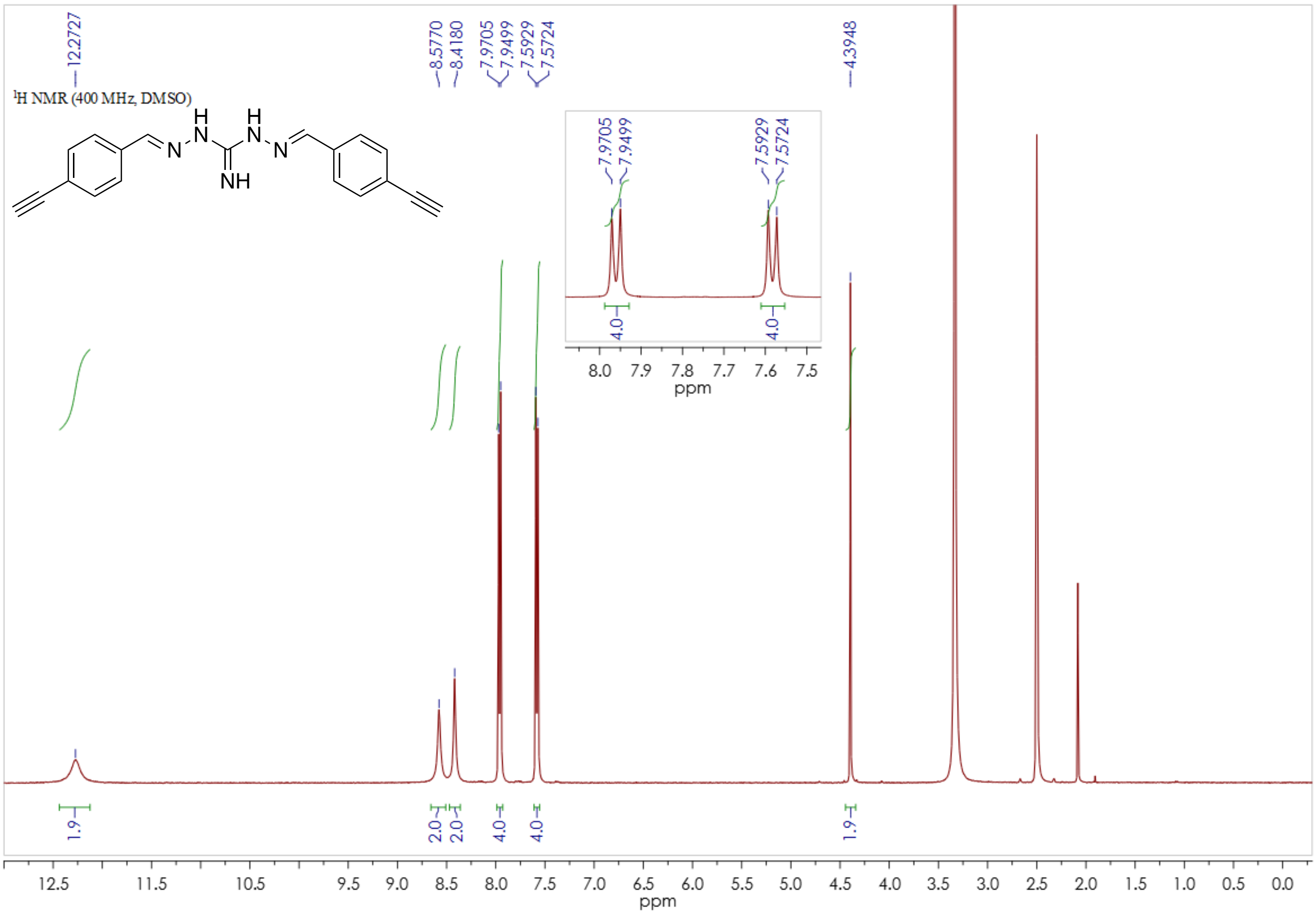

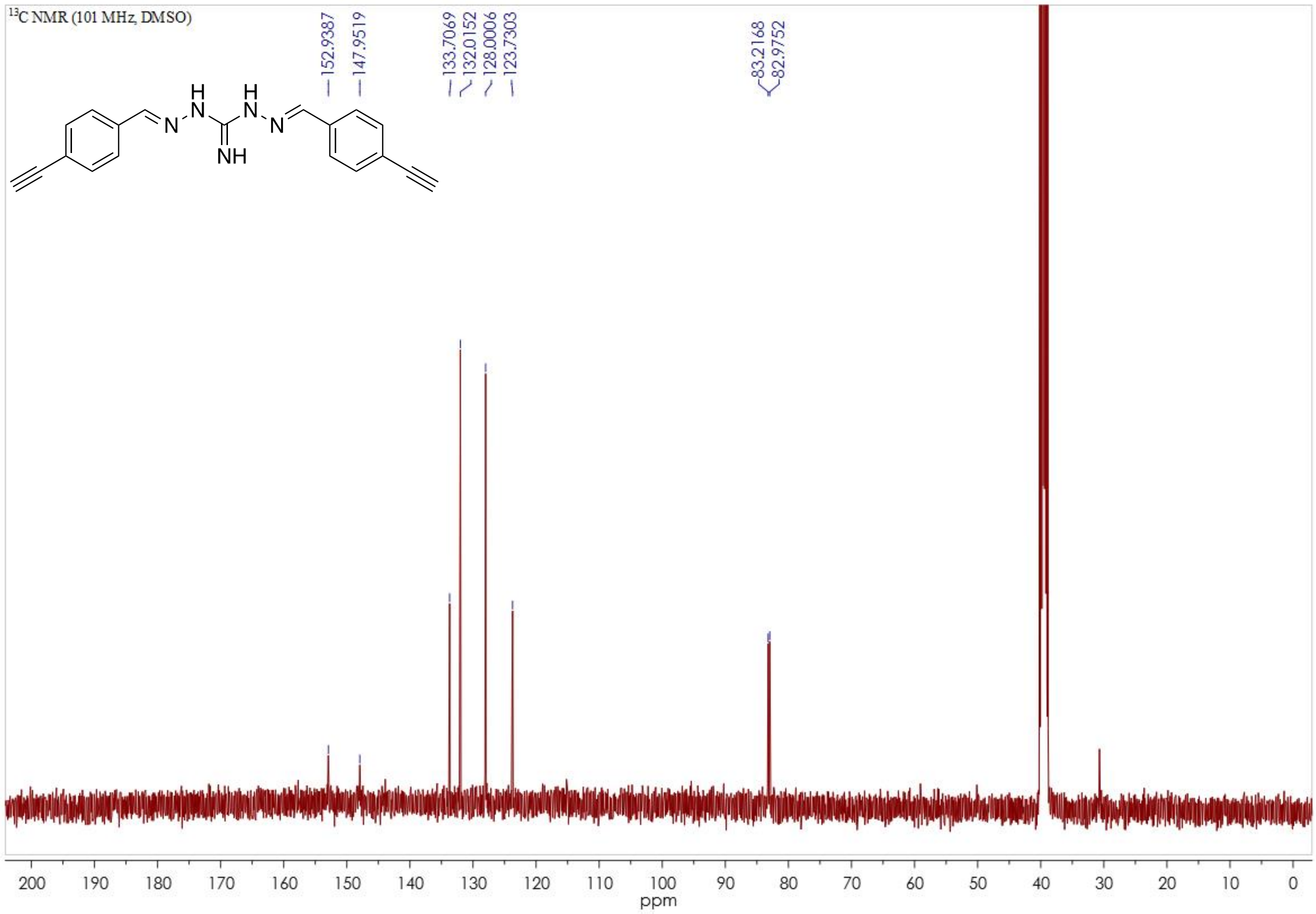

2,2'-Bis[(4-methoxyphenyl)methylene]carbonimidic dihydrazide hydrochloride (**24**)

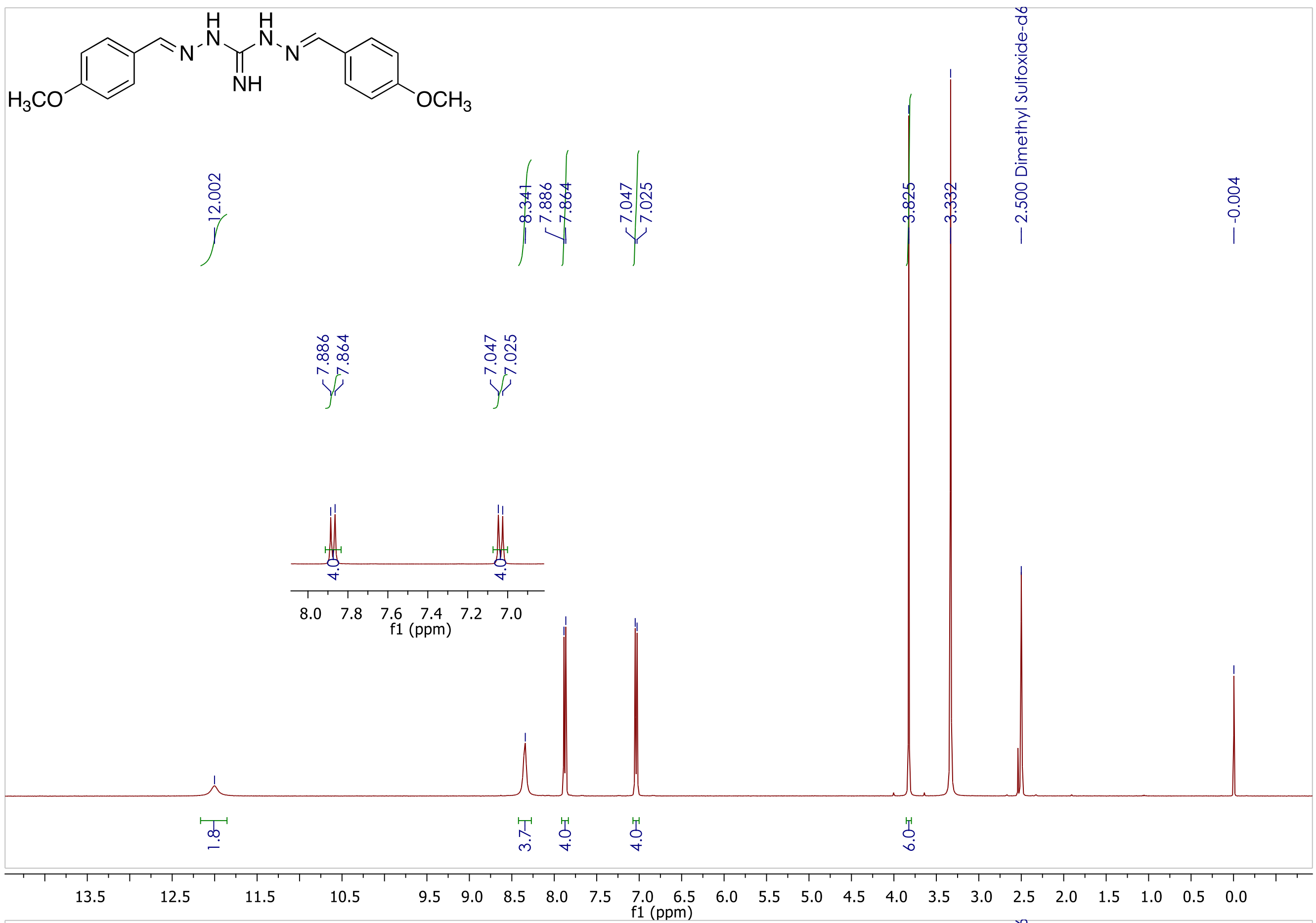

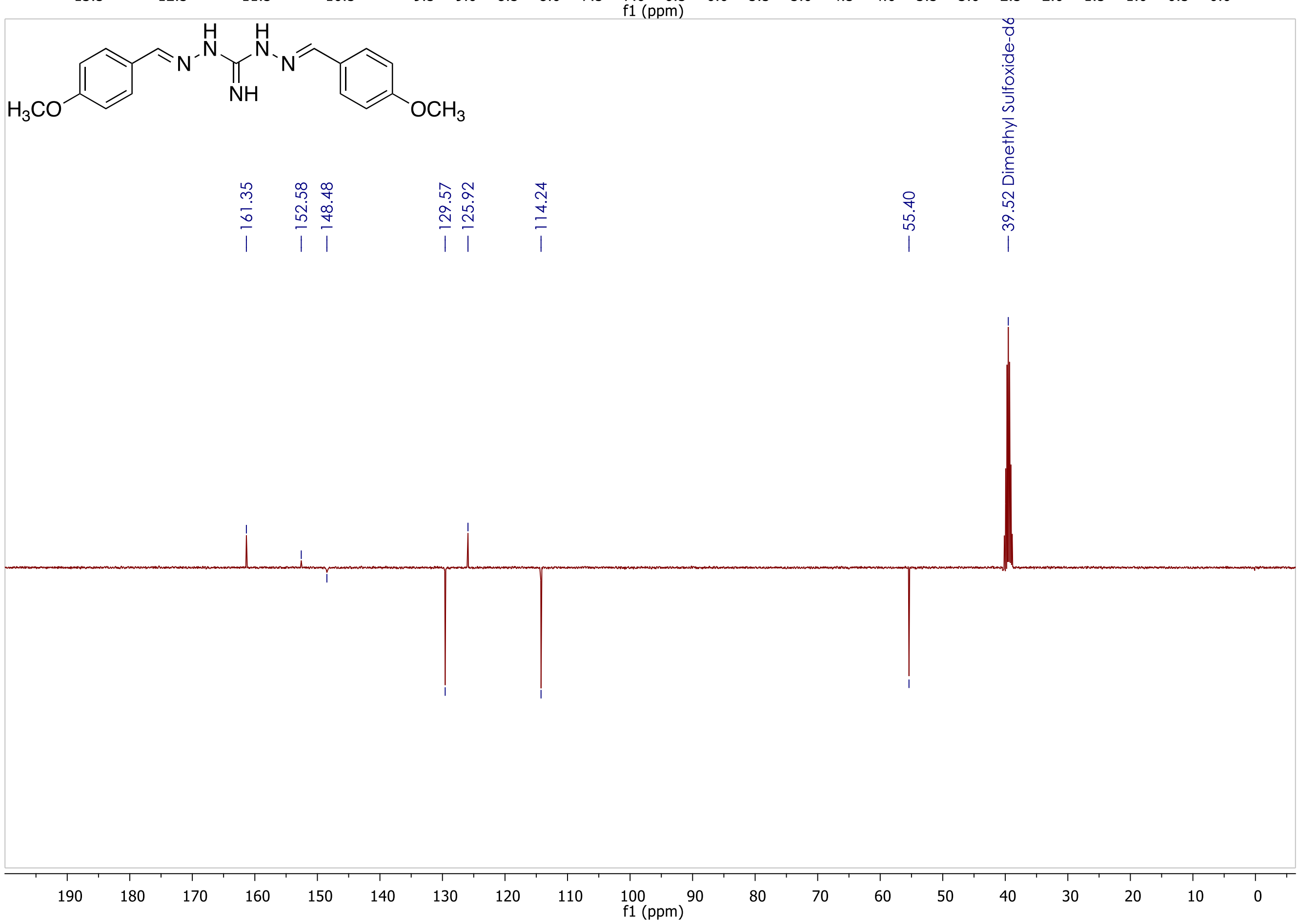

2,2'-Bis[(3-methoxyphenyl)methylene]carbonimidic dihydrazide hydrochloride (**25**)

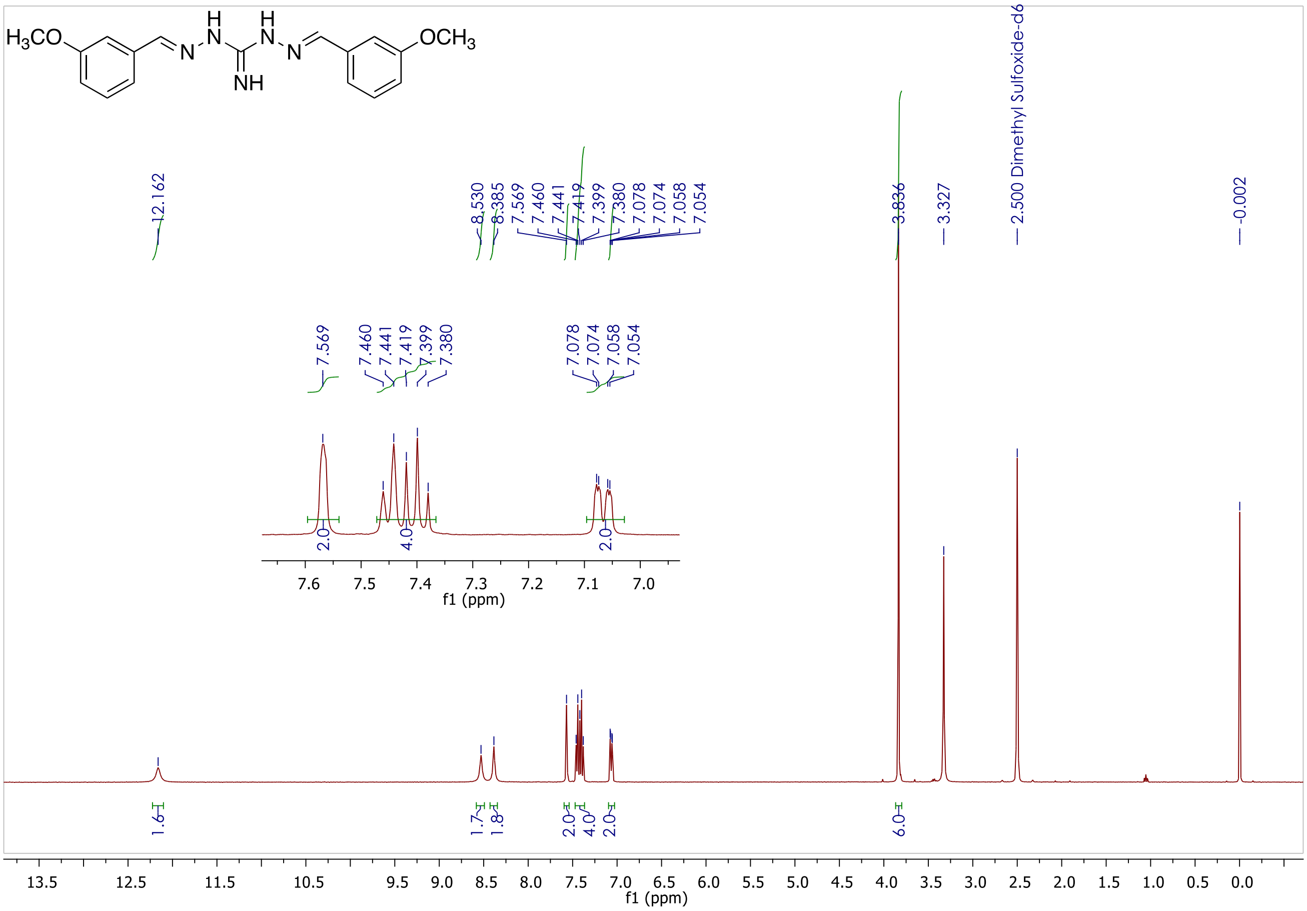

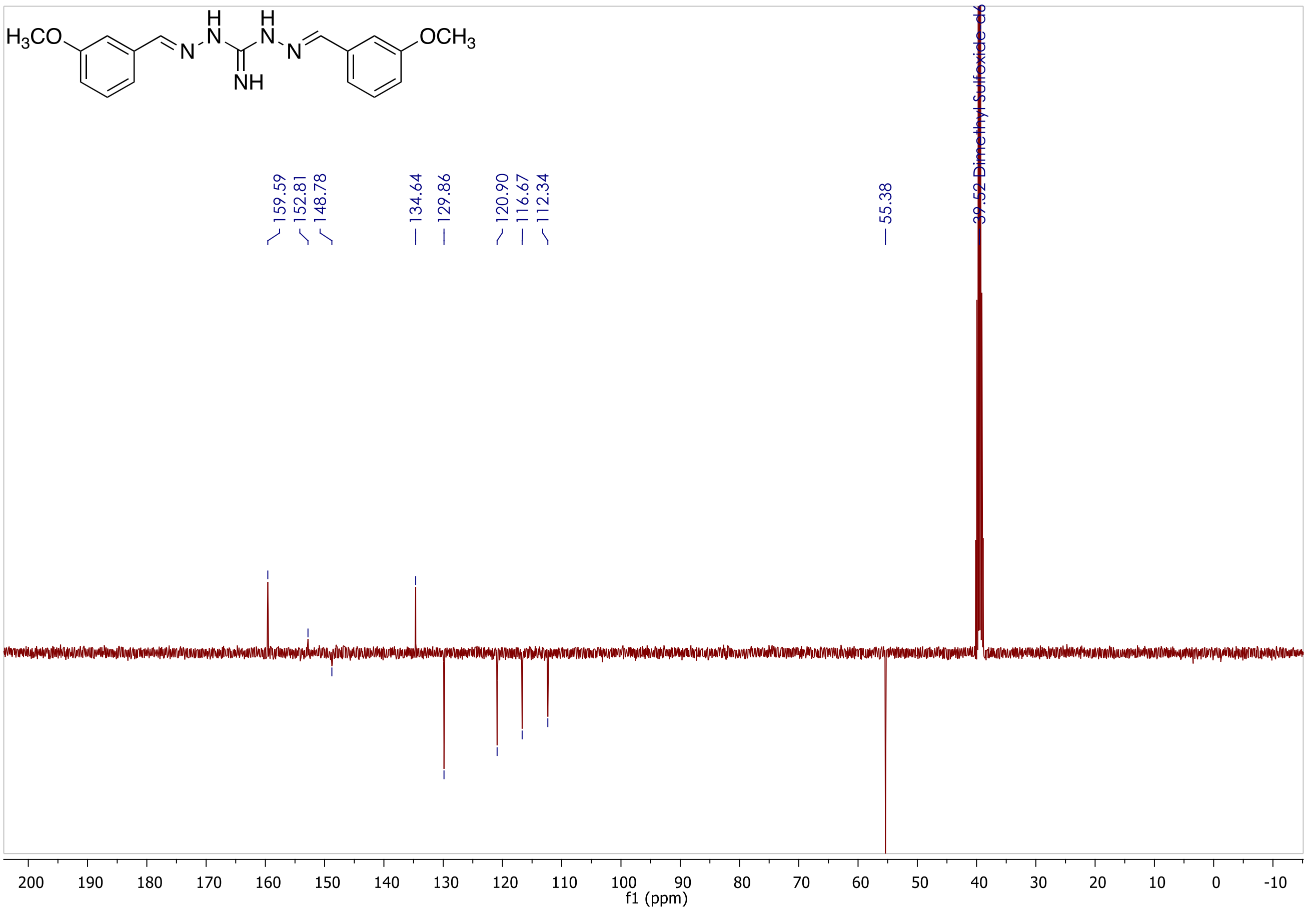

2,2'-Bis[(2-methoxyphenyl)methylene]carbonimidic dihydrazide hydrochloride (**26**)

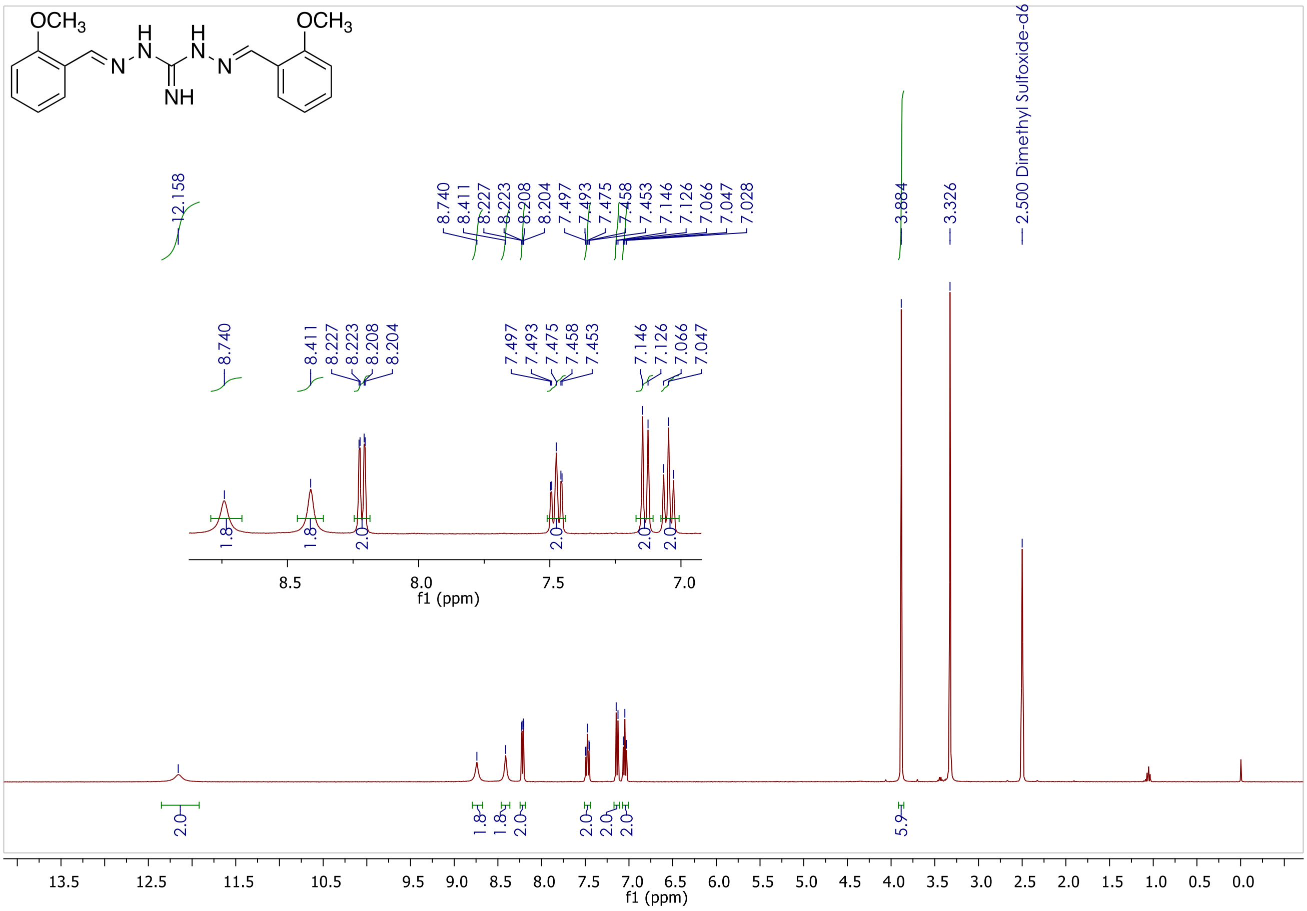

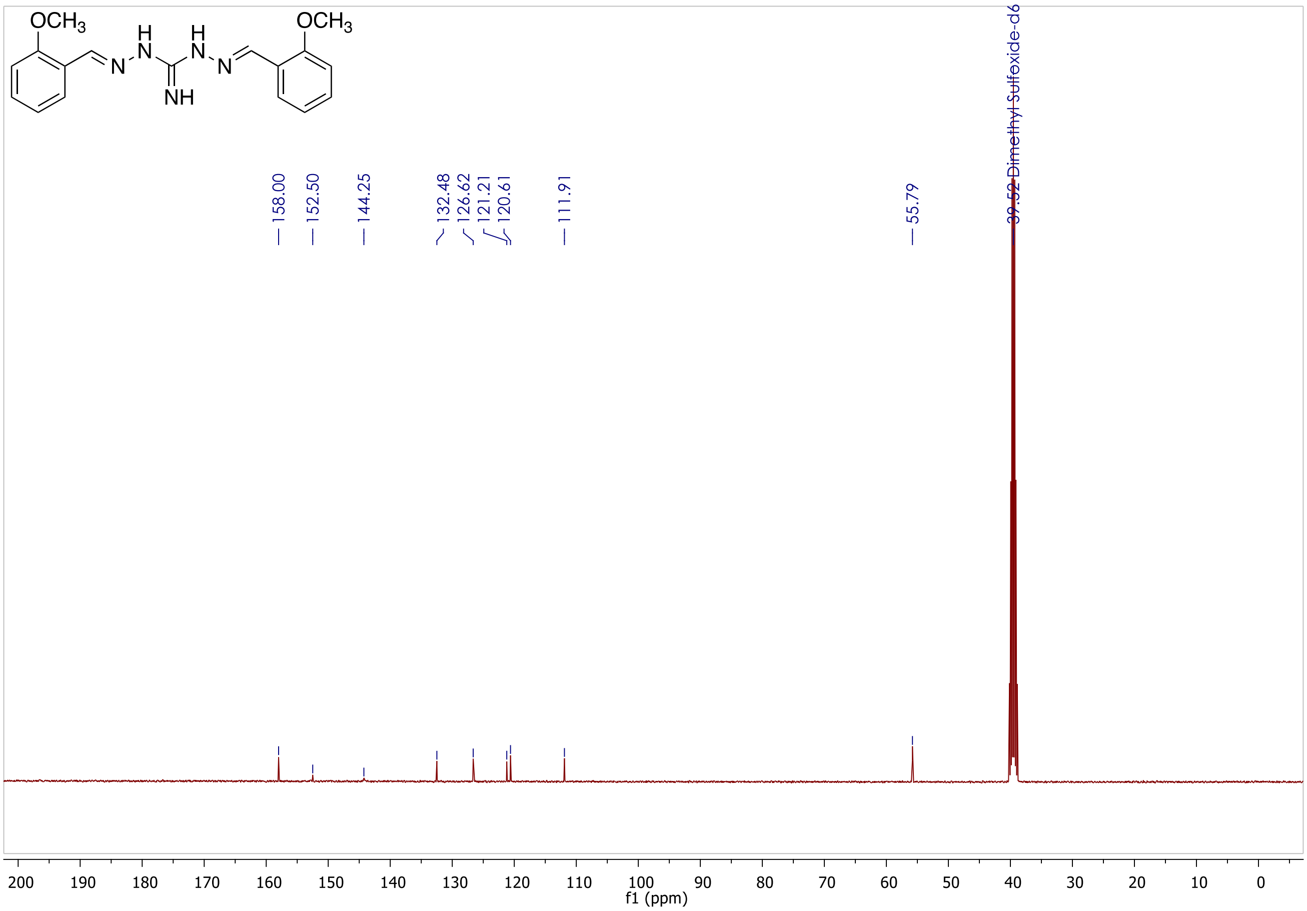

2,2'-Bis[(4-hydroxyphenyl)methylene]carbonimidic dihydrazide hydrochloride (**27**)

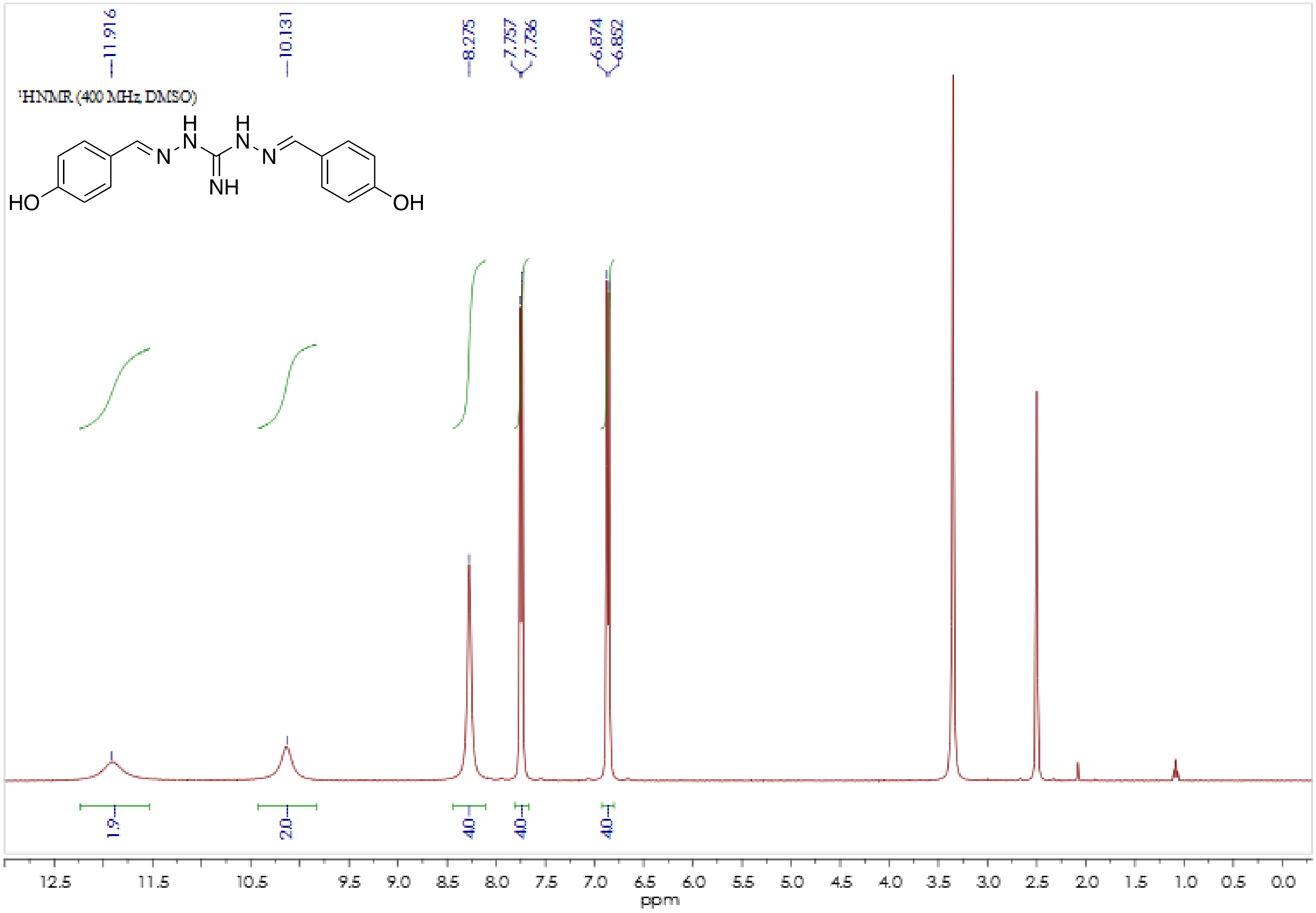

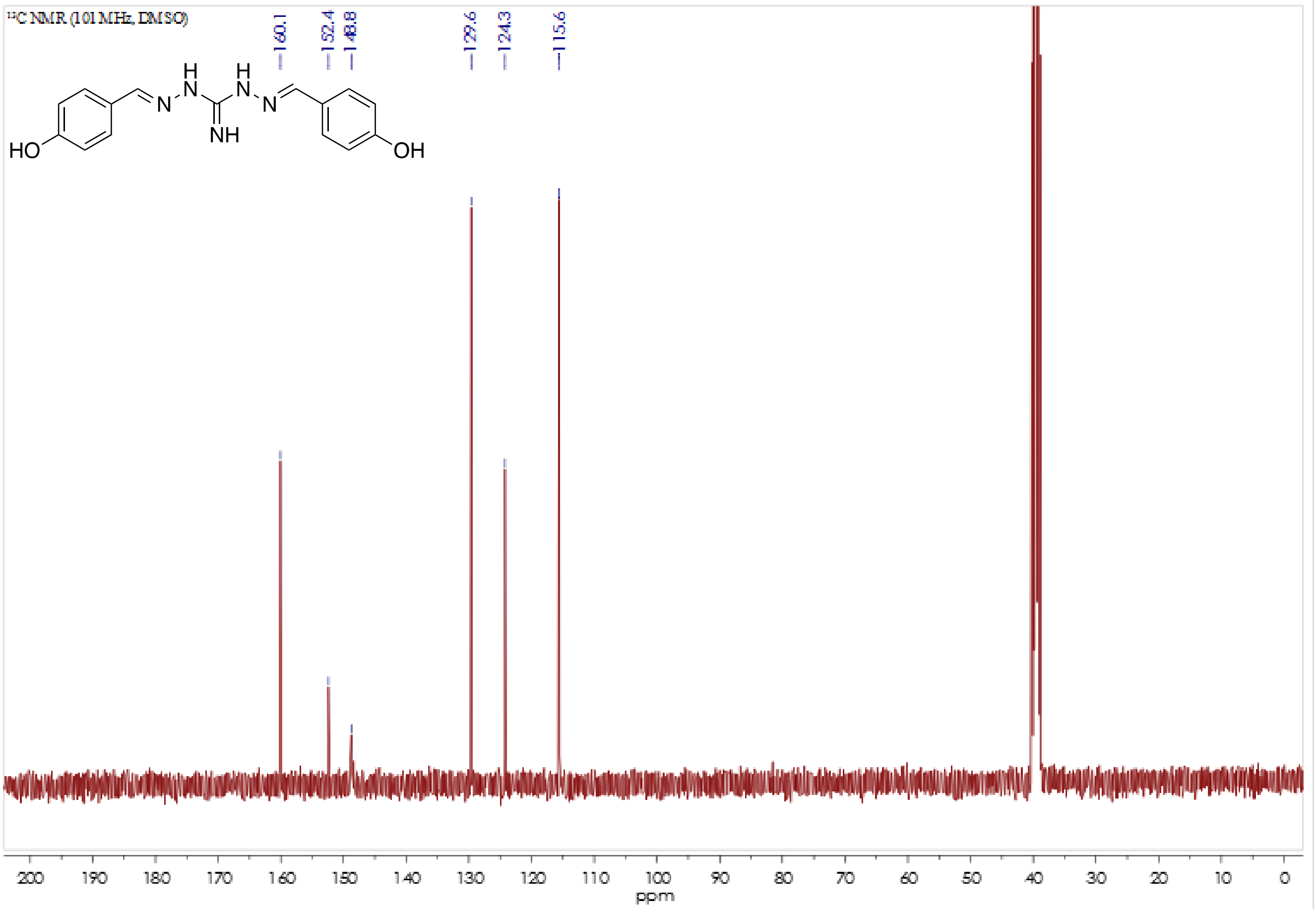

2,2'-Bis[(3-hydroxyphenyl)methylene]carbonimidic dihydrazide hydrochloride (**28**)

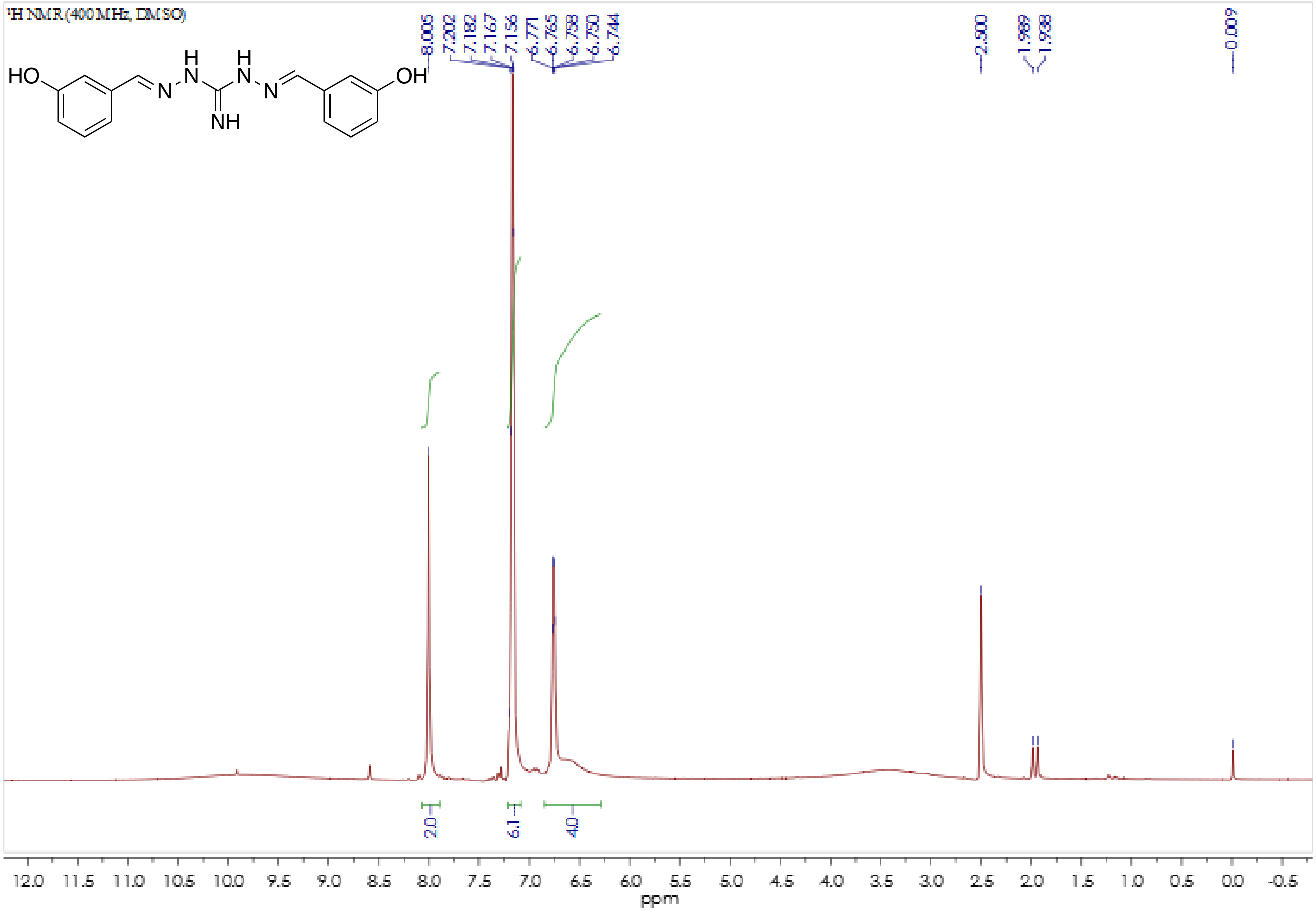

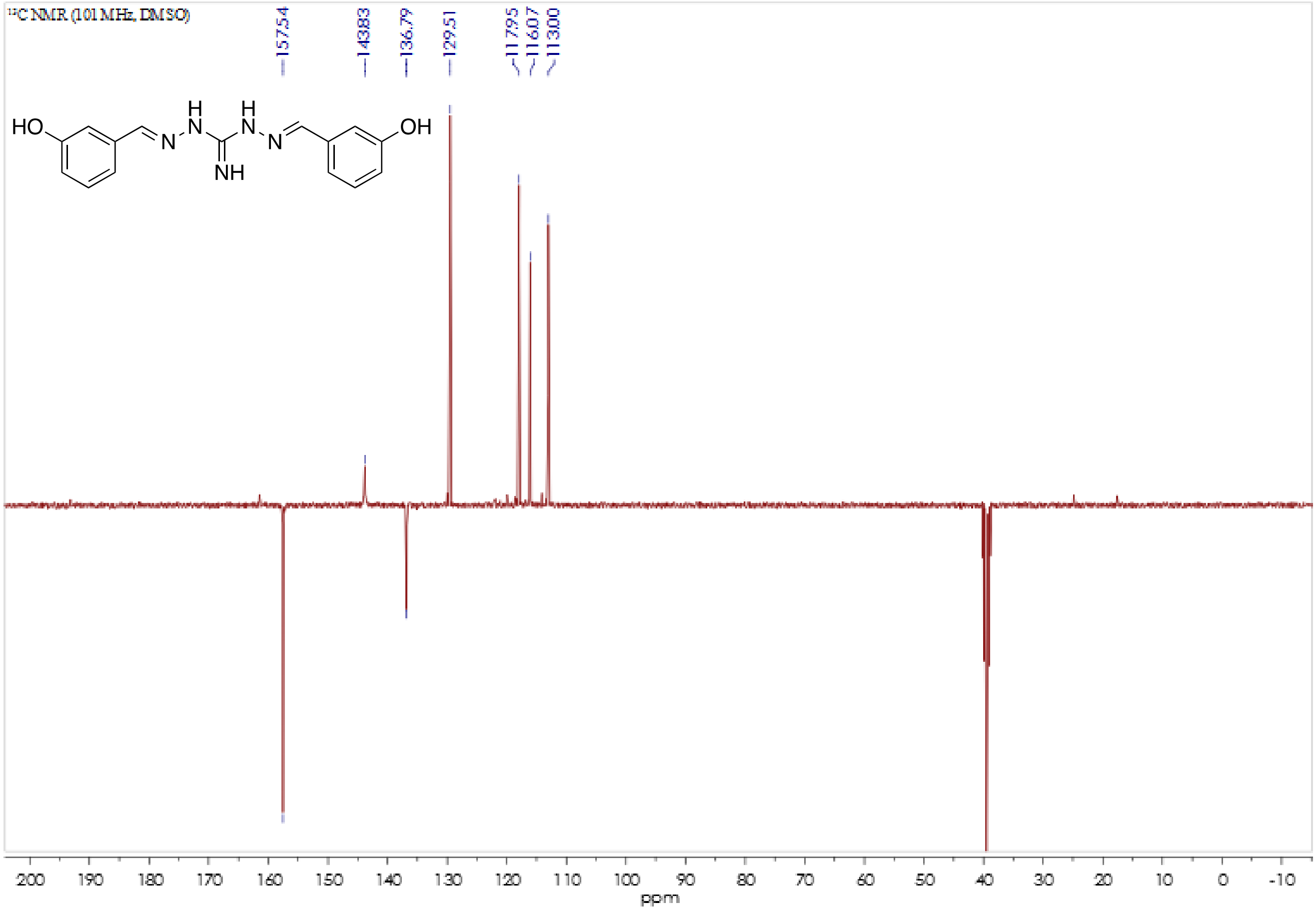

*2,2'-Bis[(2-hydroxyphenyl)methylene]carbonimidic dihydrazide hydrochloride (**29**)*

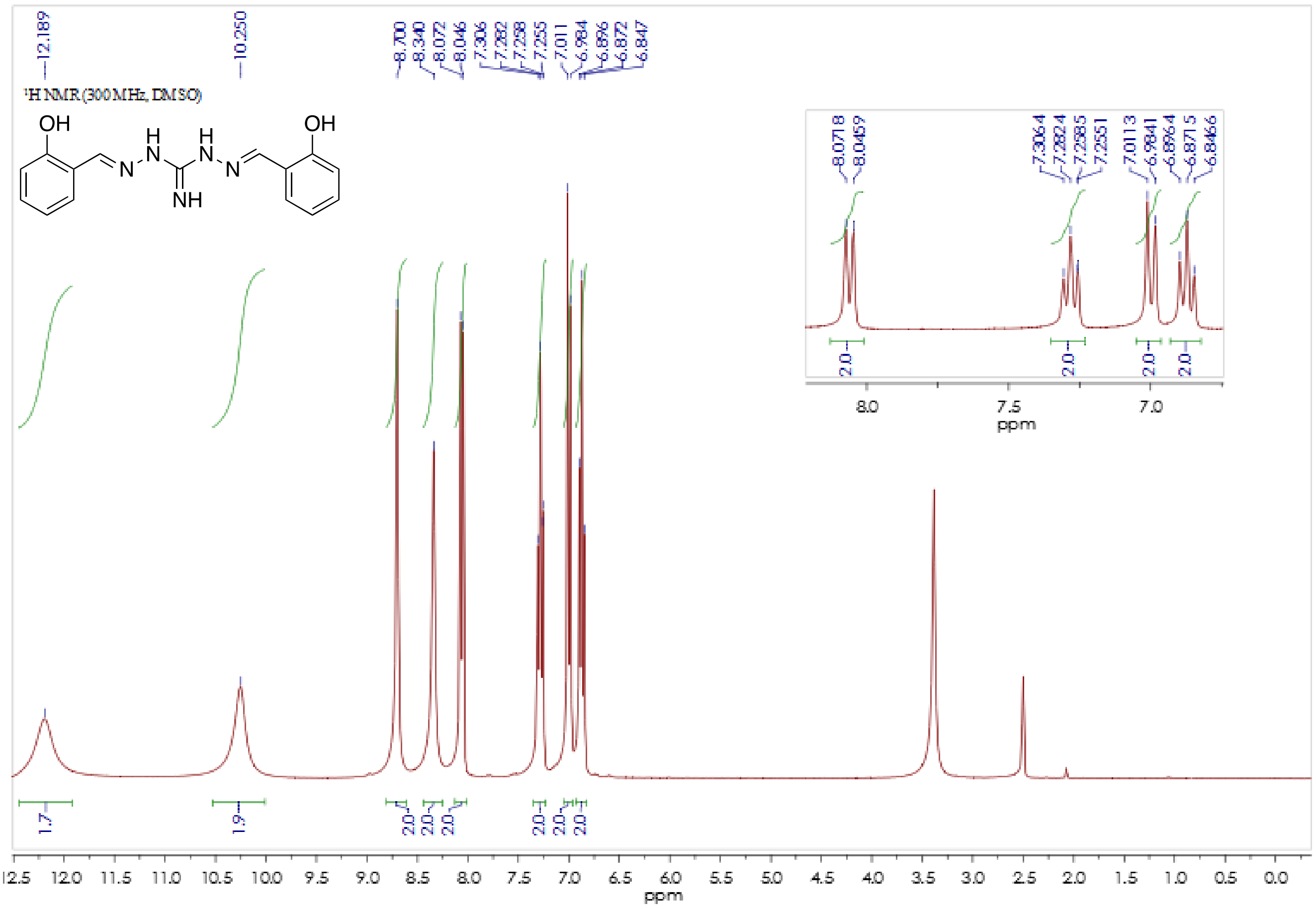

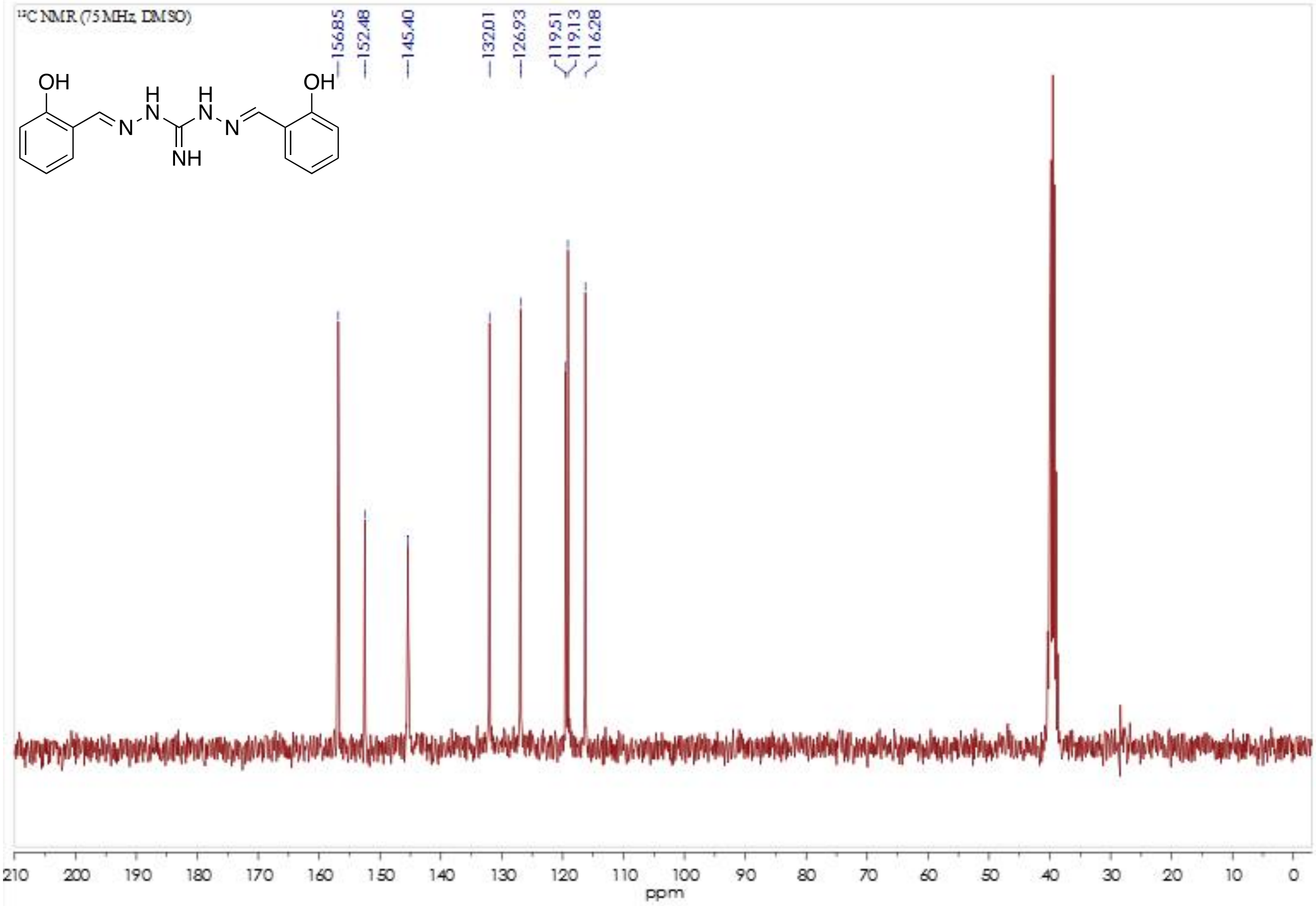

2,2'-bis{[4-(trifluoromethoxy)phenyl]methylene}carbonimidic dihydrazide hydrochloride (**30**)

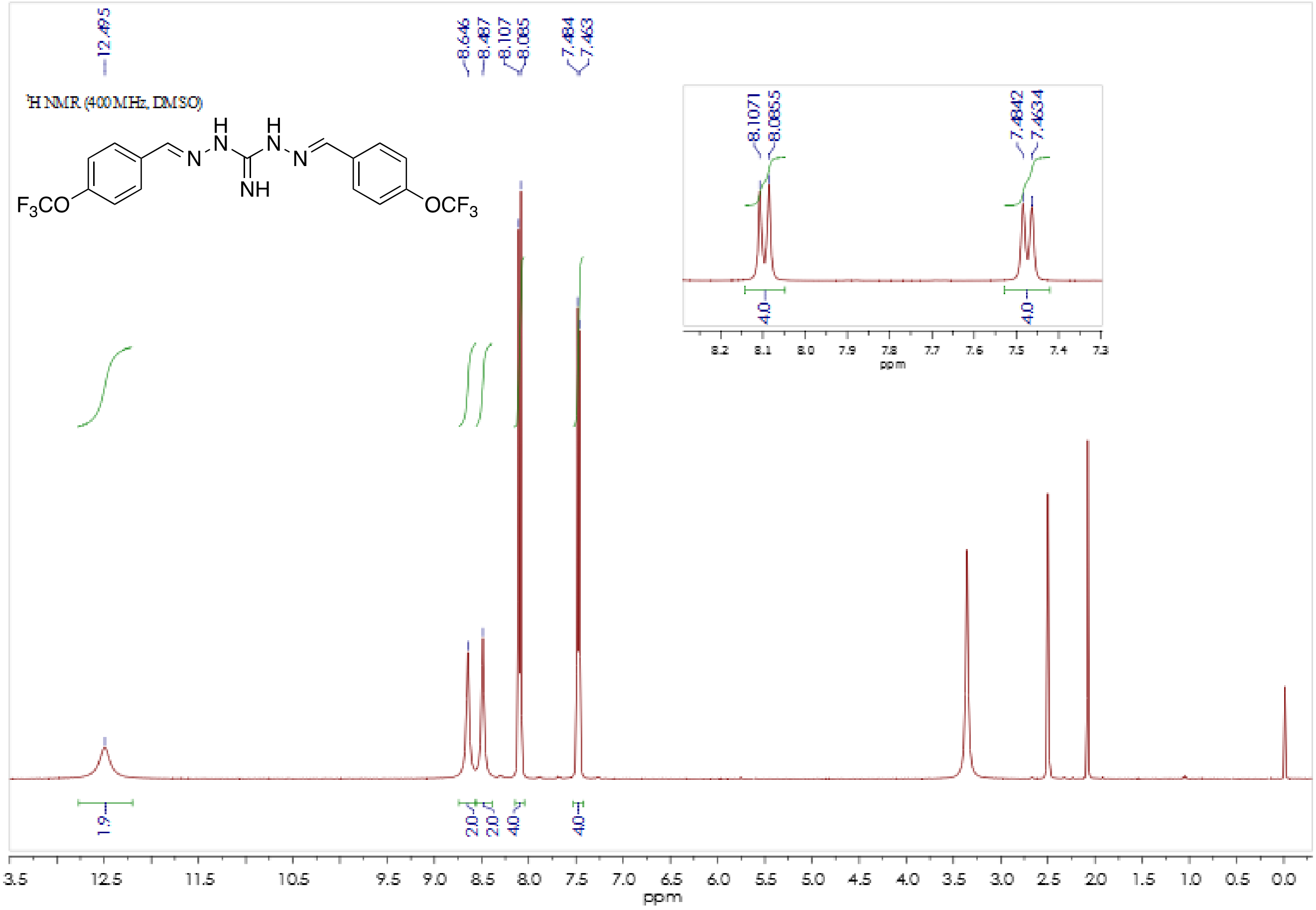

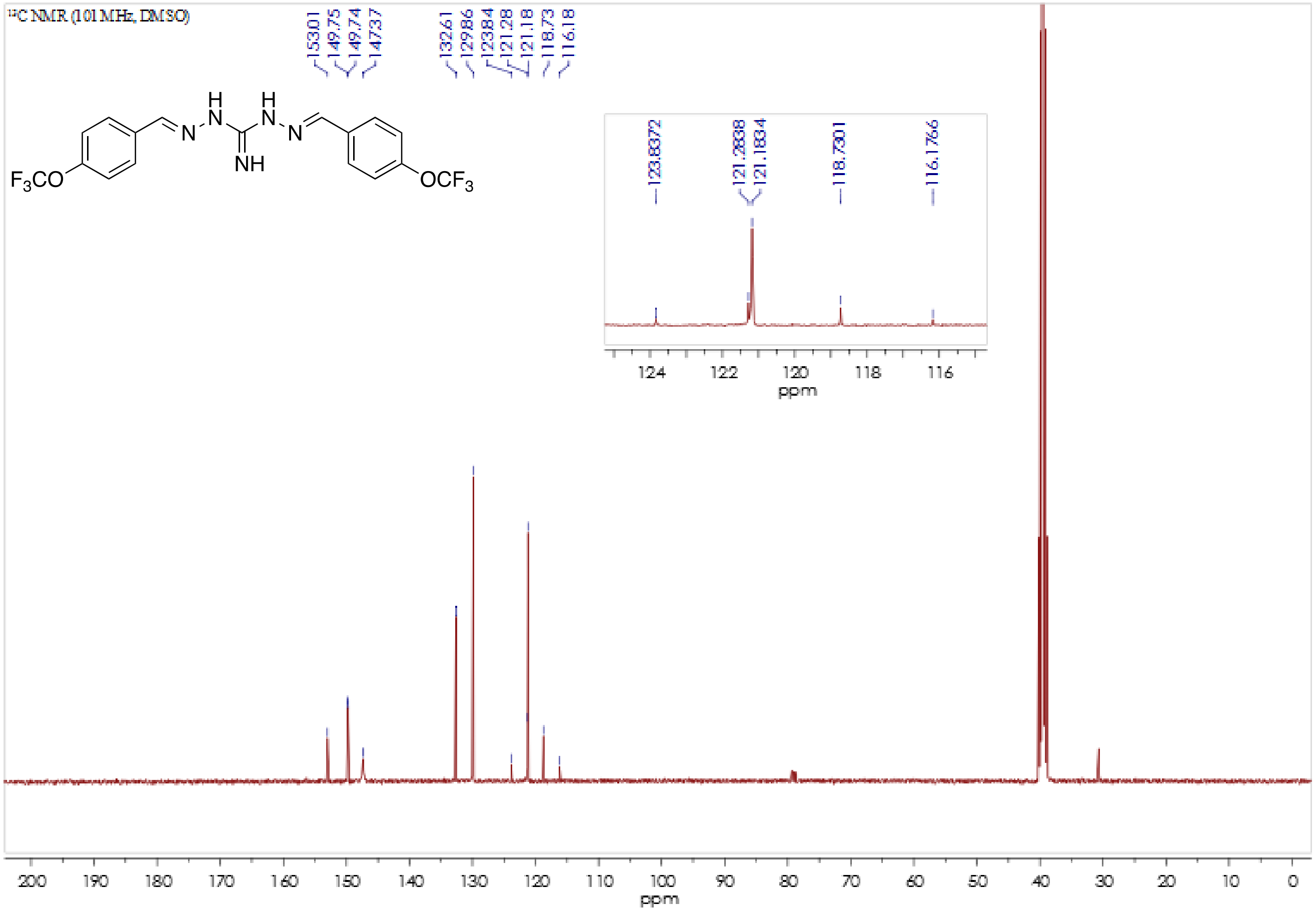

2,2'-Bis{[4-(methylsulfanyl)phenyl]methylene}carbonimidic dihydrazide hydrochloride (**31**)

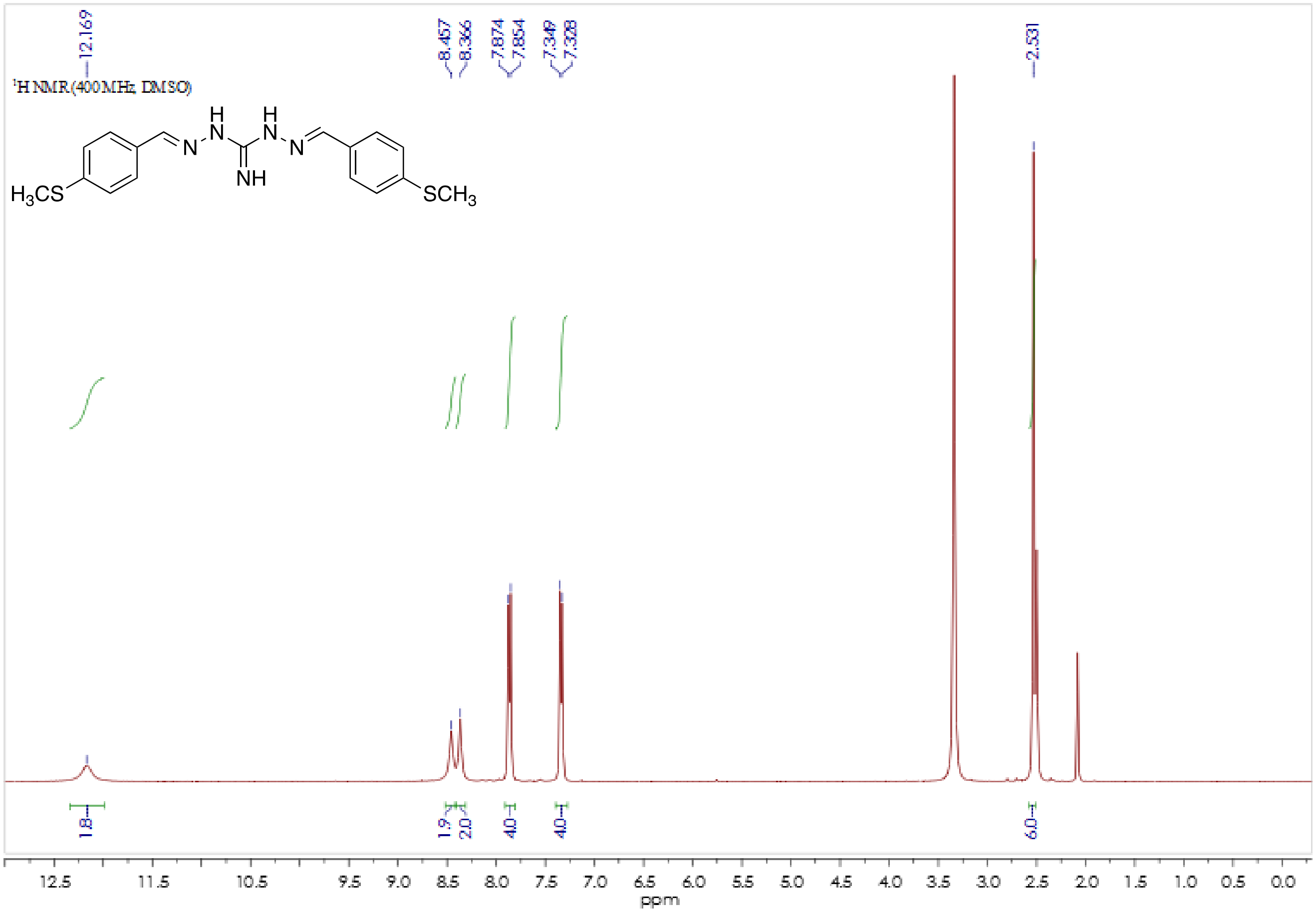

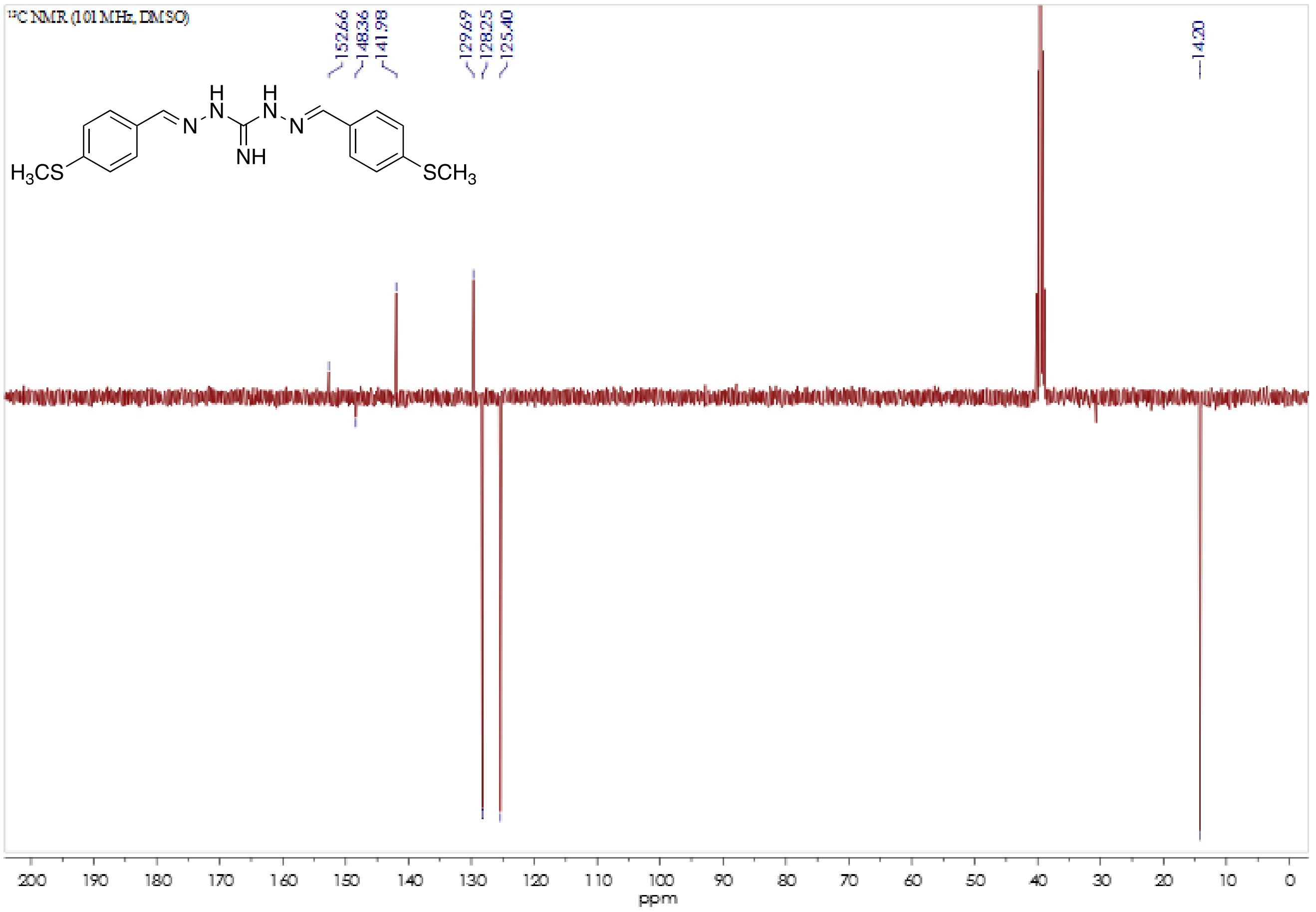

2,2'-Bis{[4-(trifluoromethylsulfanyl)phenyl]methylene}carbonimidic dihydrazide hydrochloride (**32**)

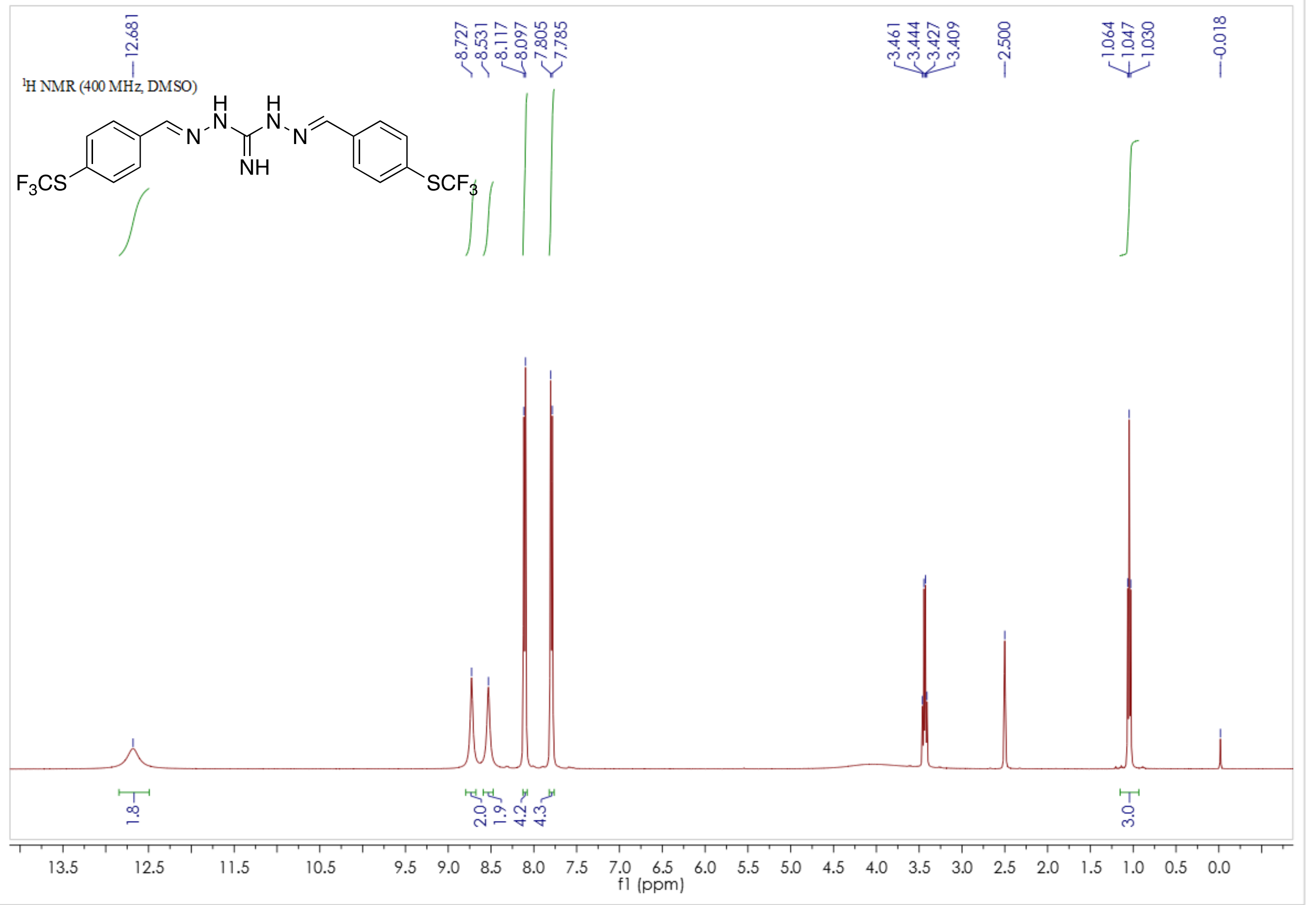

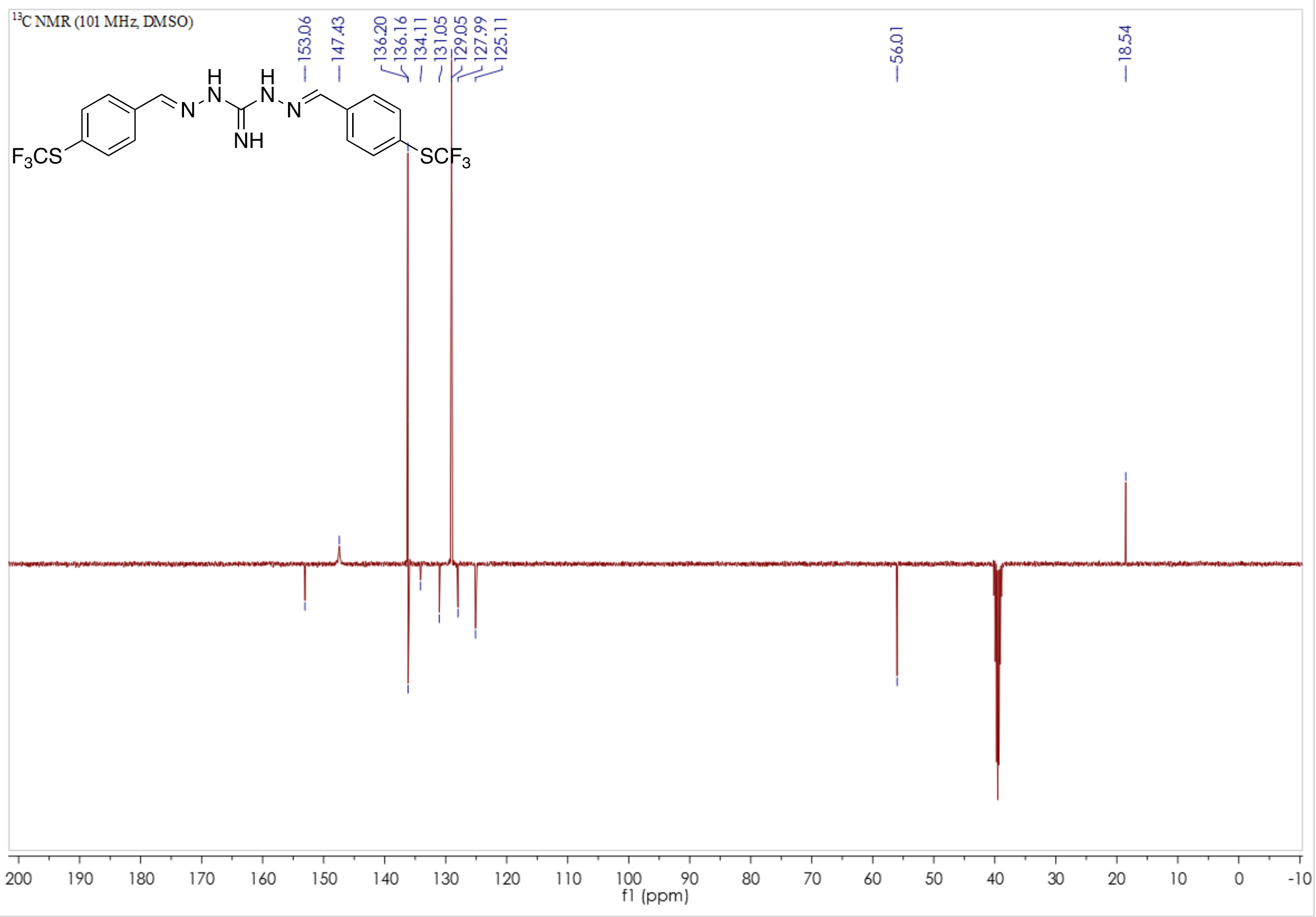

*2,2'-Bis{4-(dimethylamino)phenyl]methylene}carbonimidic dihydrazide hydrochloride (**33**)*

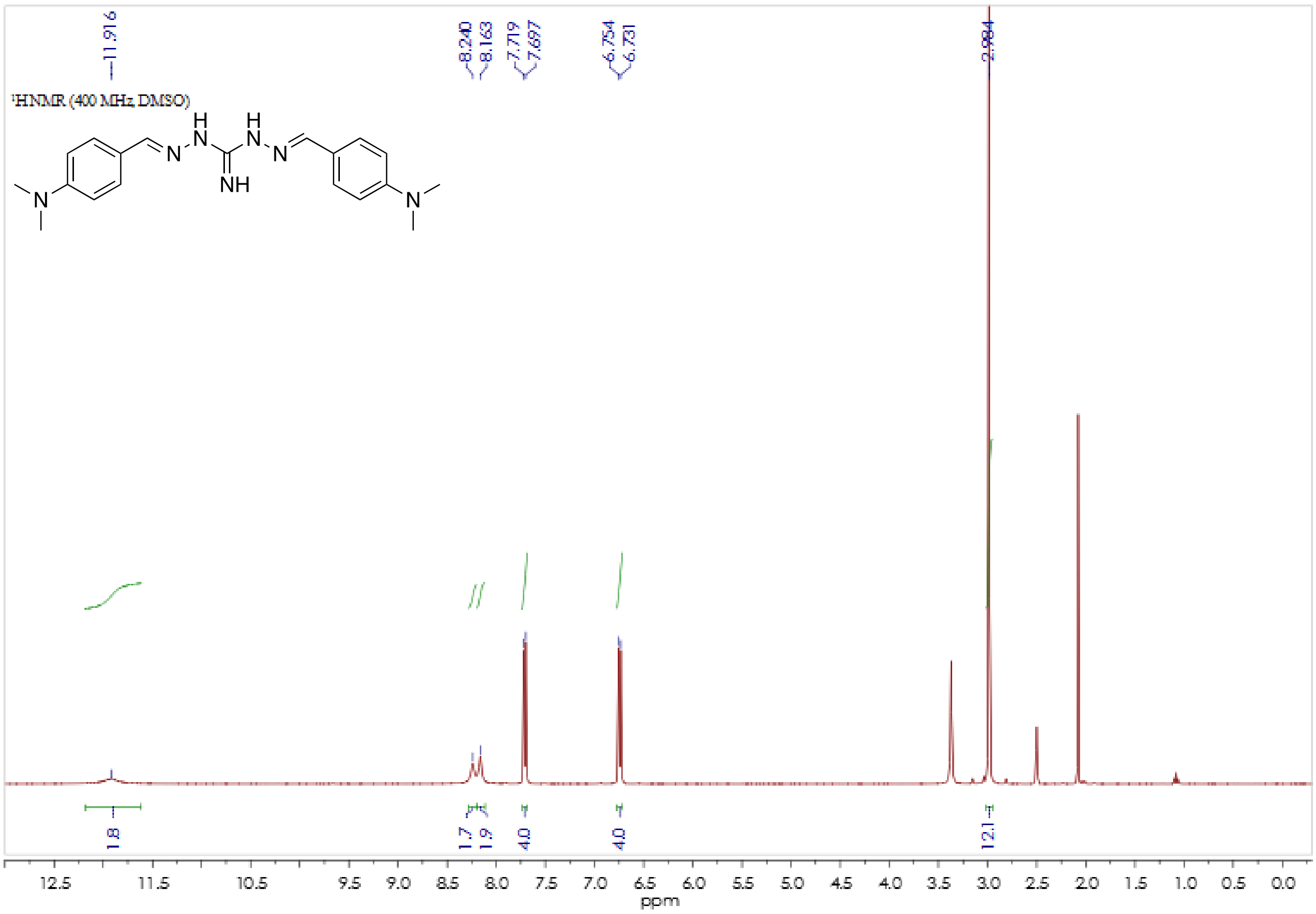

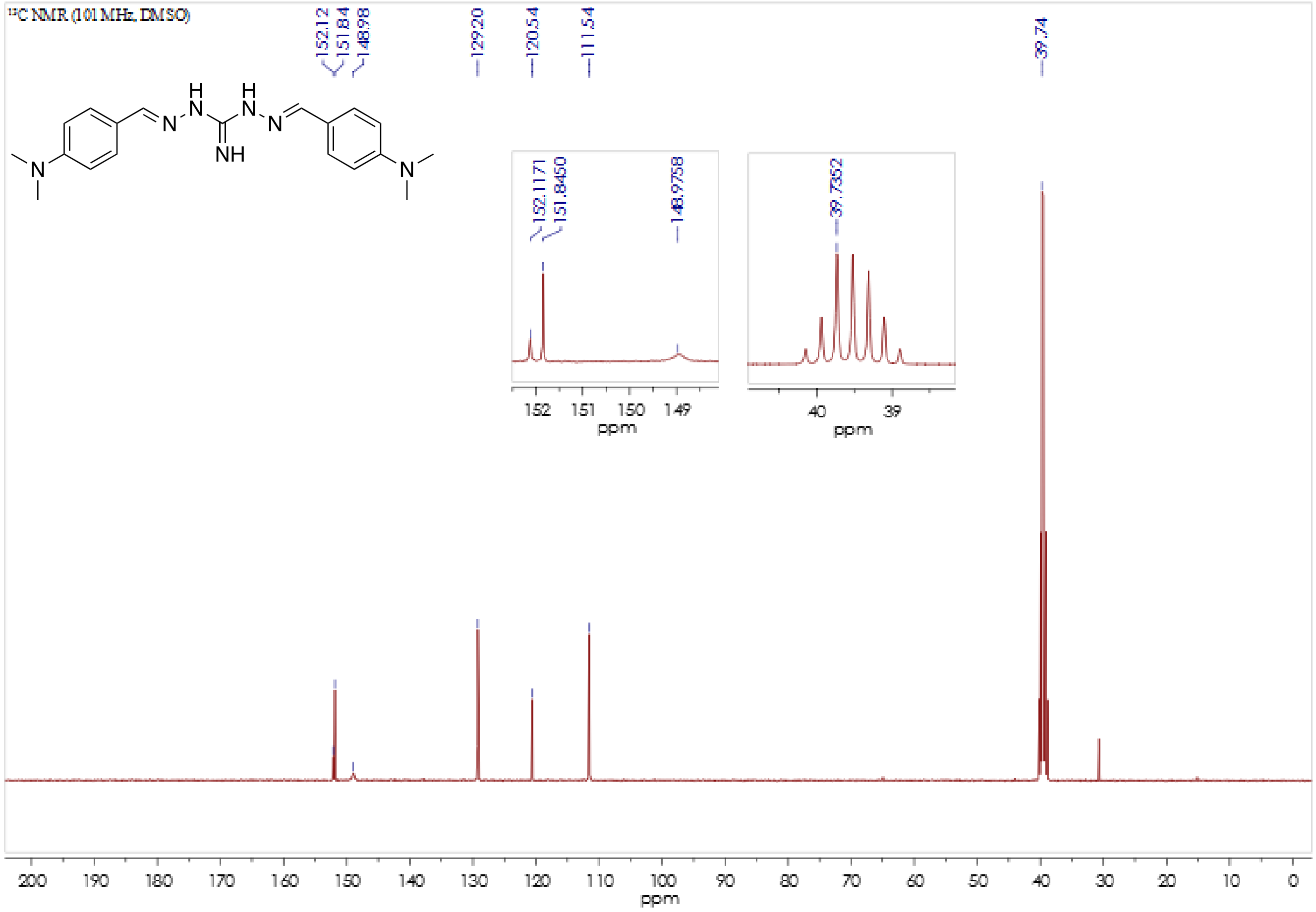

2,2'-Bis[(2,4-dichlorophenyl)methylene]carbonimidic dihydrazide hydrochloride (**34**)

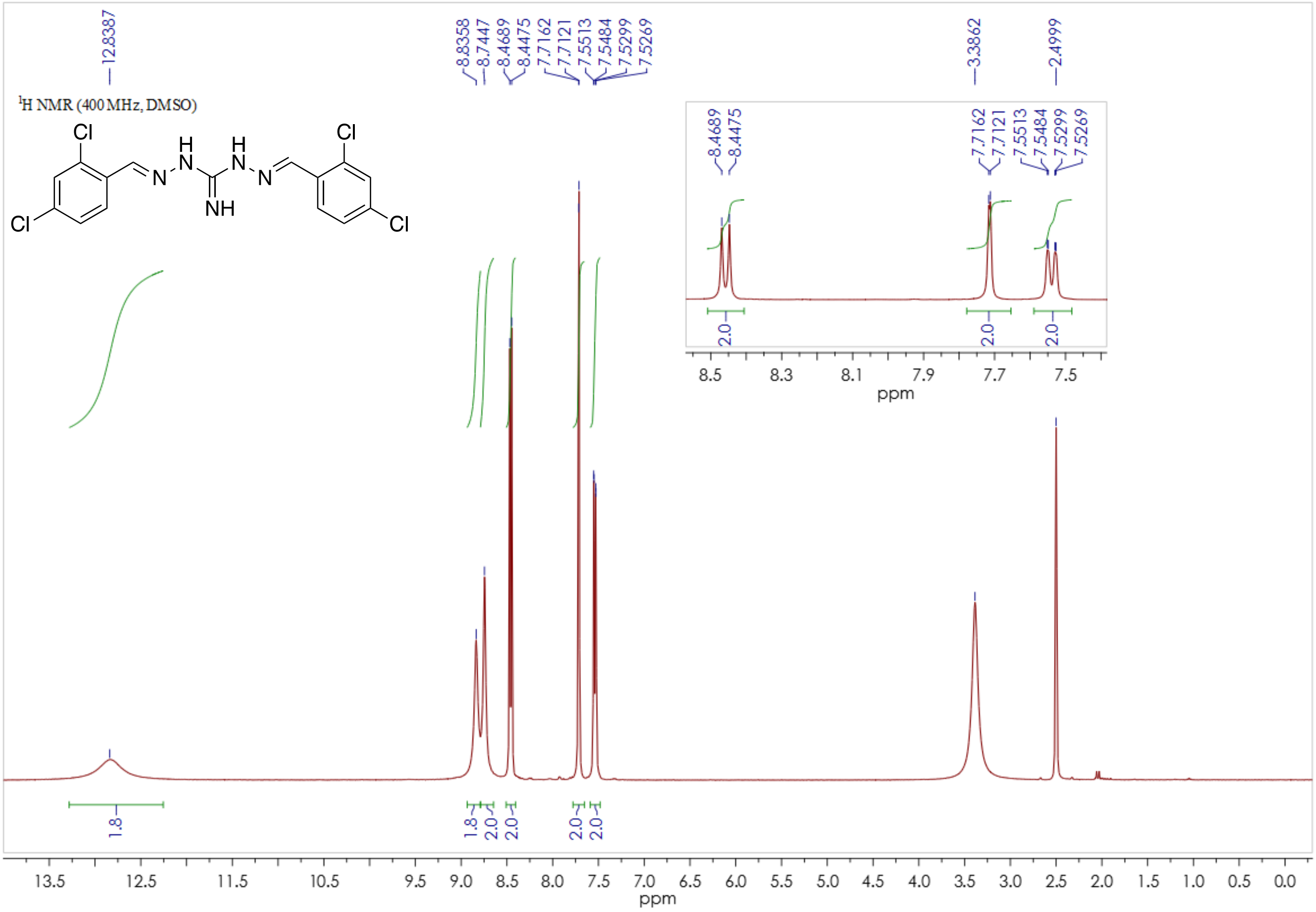

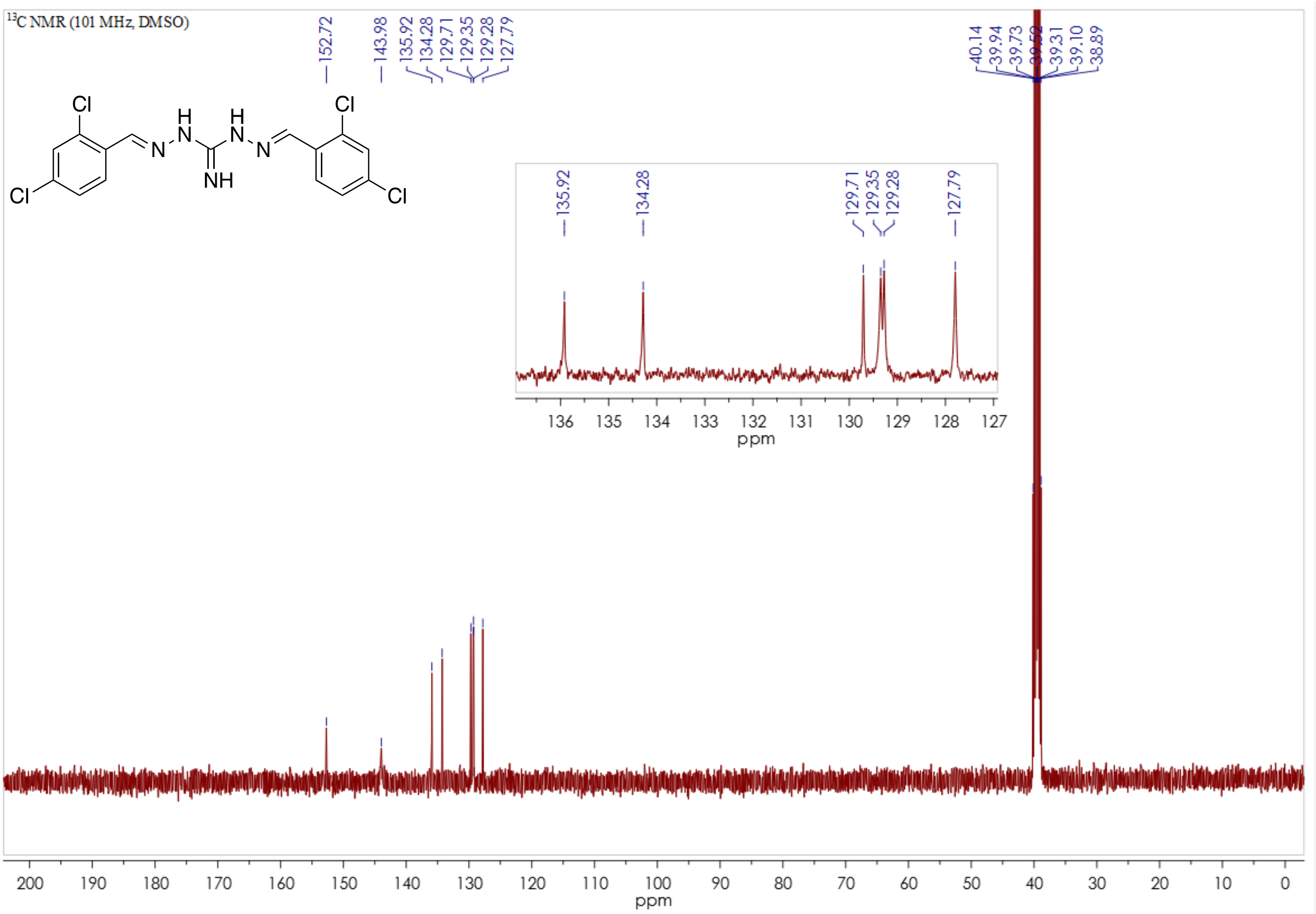

2,2'-Bis[(3,5-dichlorophenyl)methylene]carbonimidic dihydrazide hydrochloride (**35**)

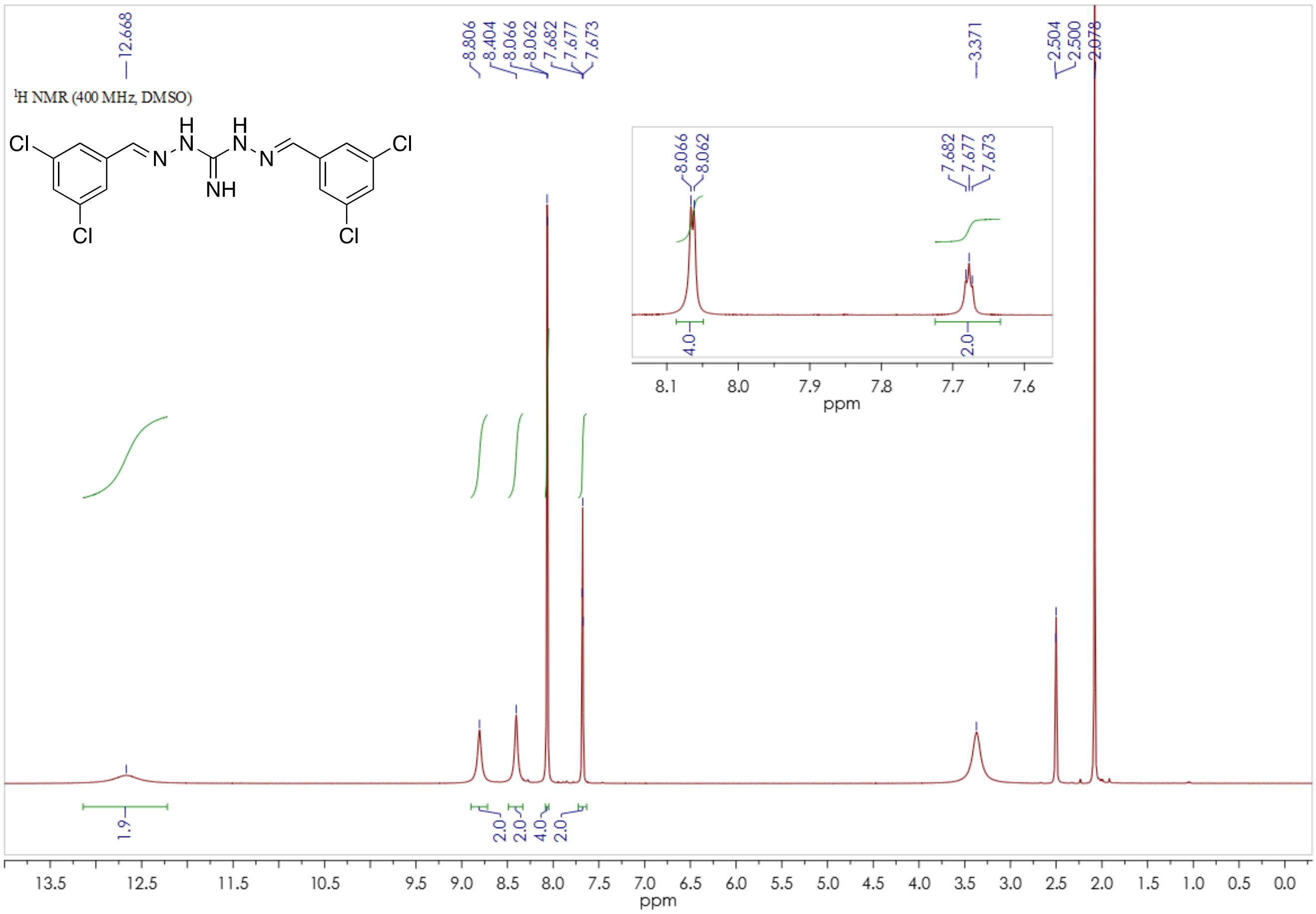

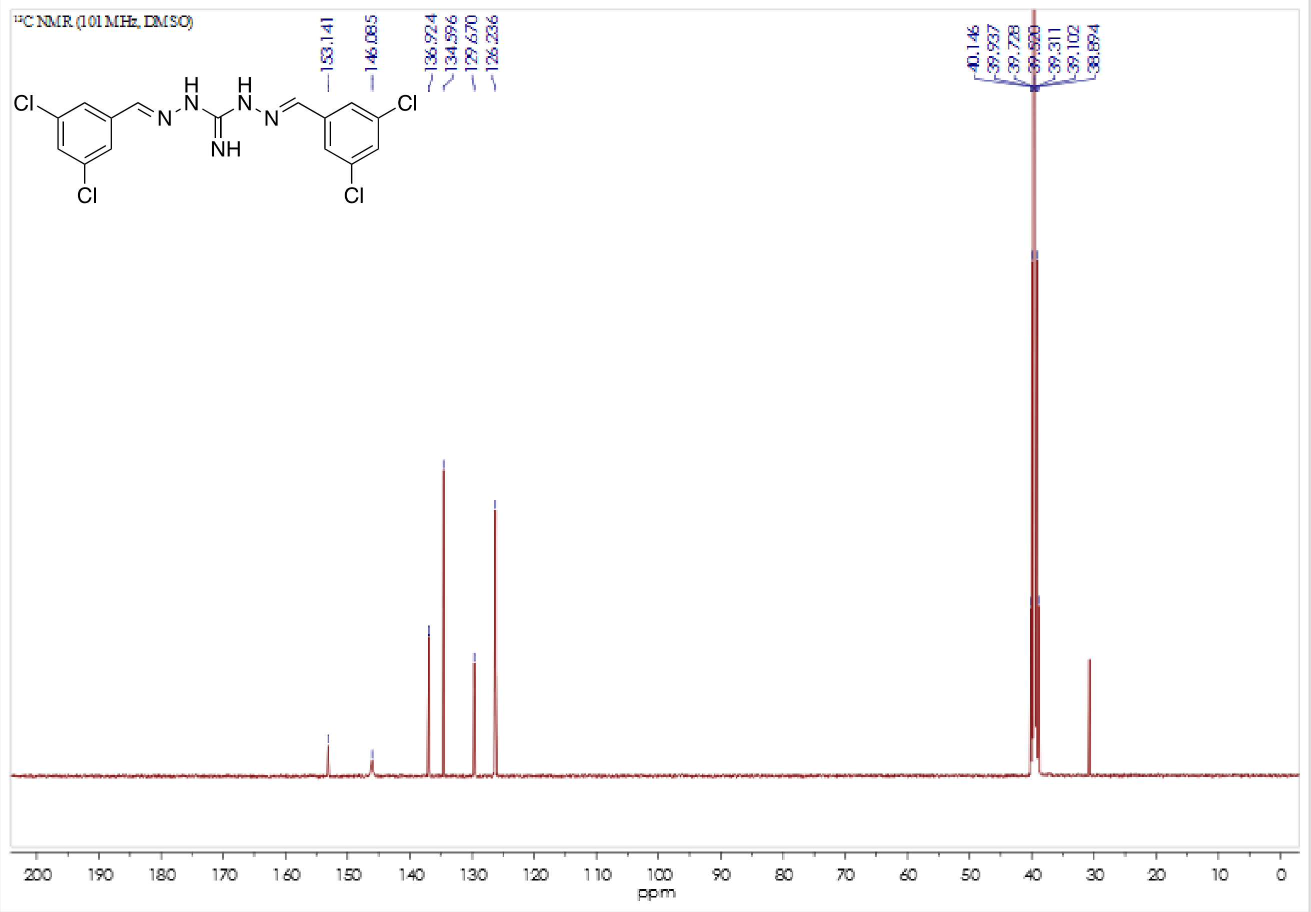

2,2'-Bis[(2,6-dichlorophenyl)methylene]carbonimidic dihydrazide hydrochloride (**36**)

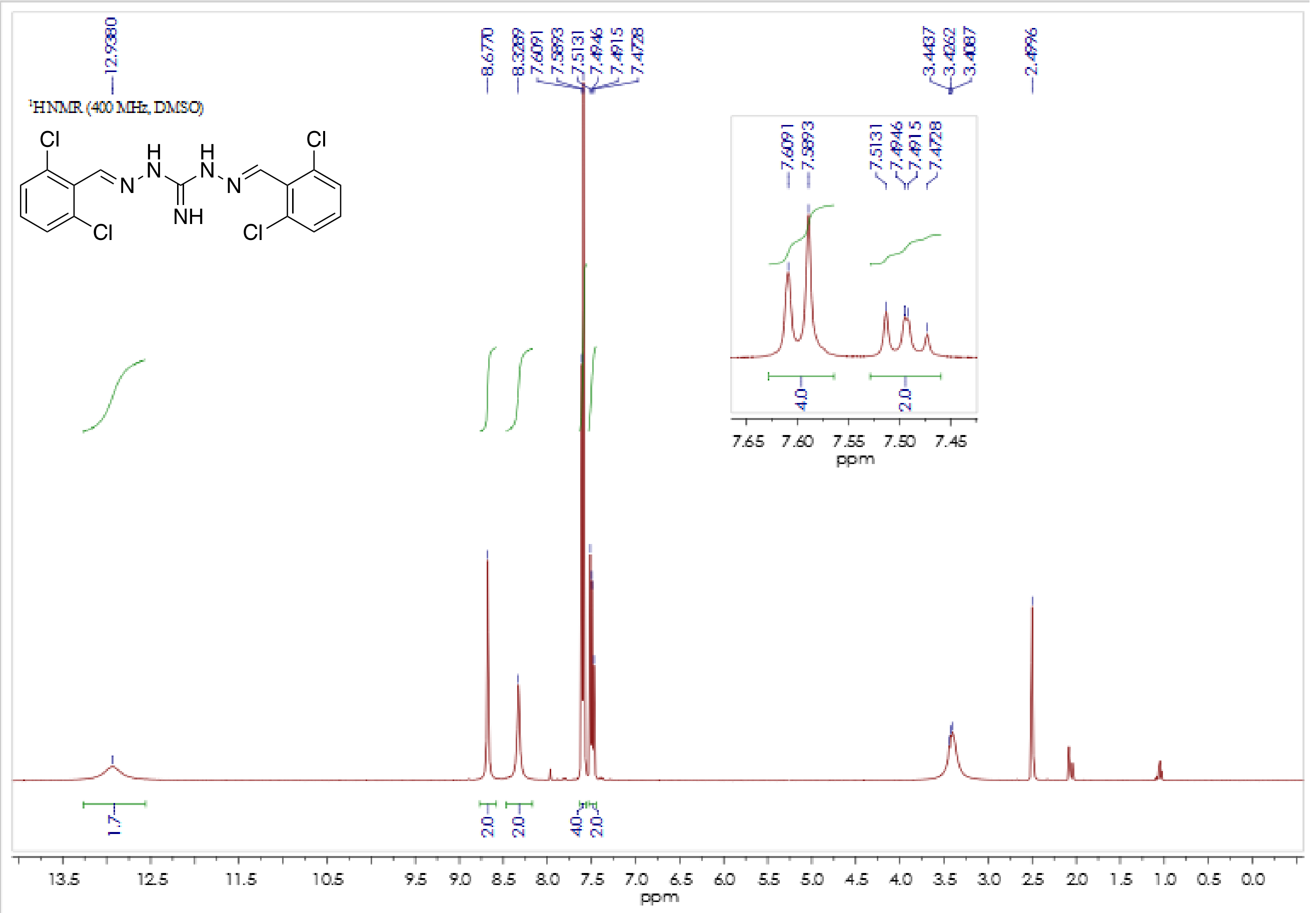

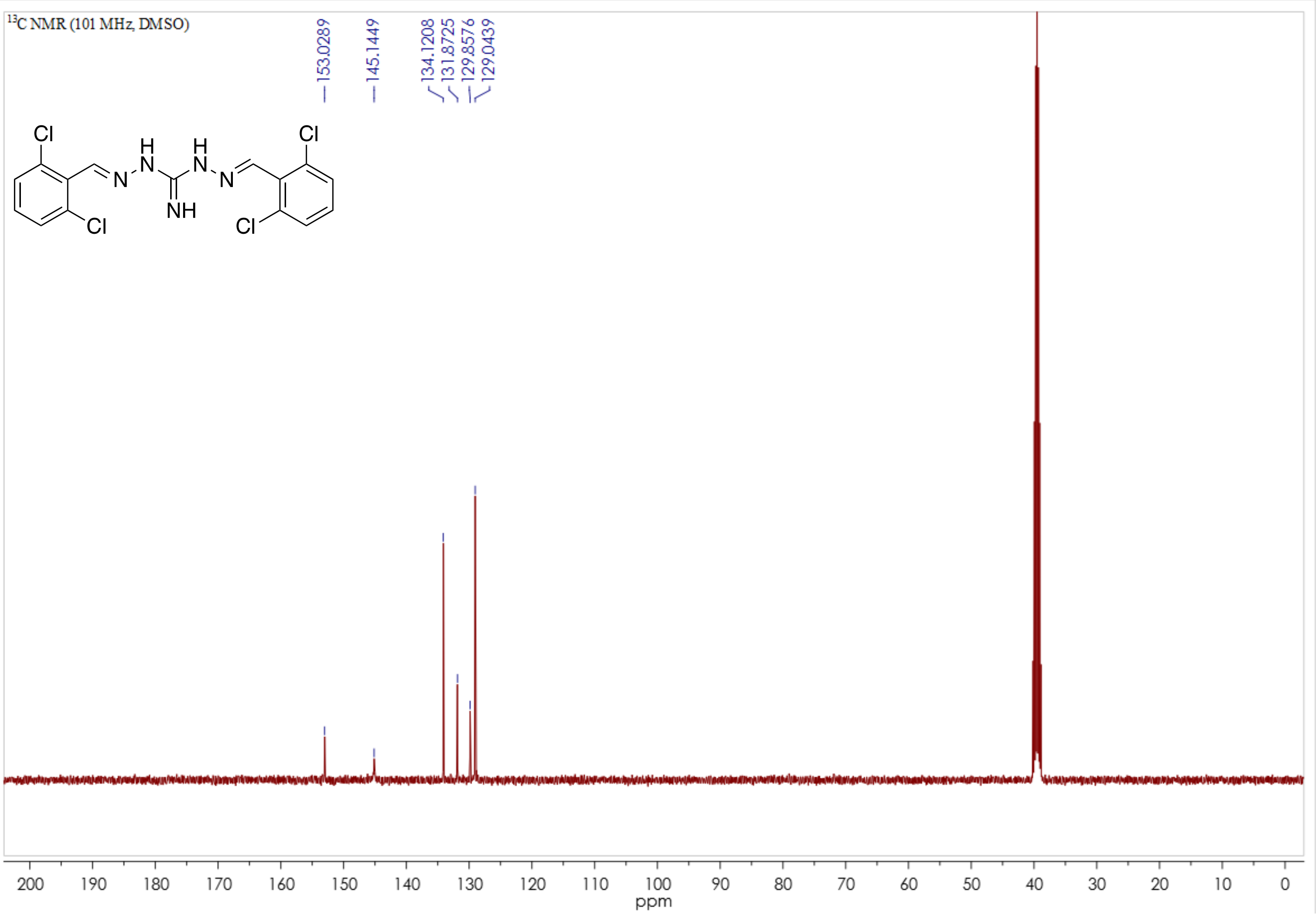

2,2'-Bis[(2,5-difluorophenyl)methylene]carbonimidic dihydrazide hydrochloride (**37**)

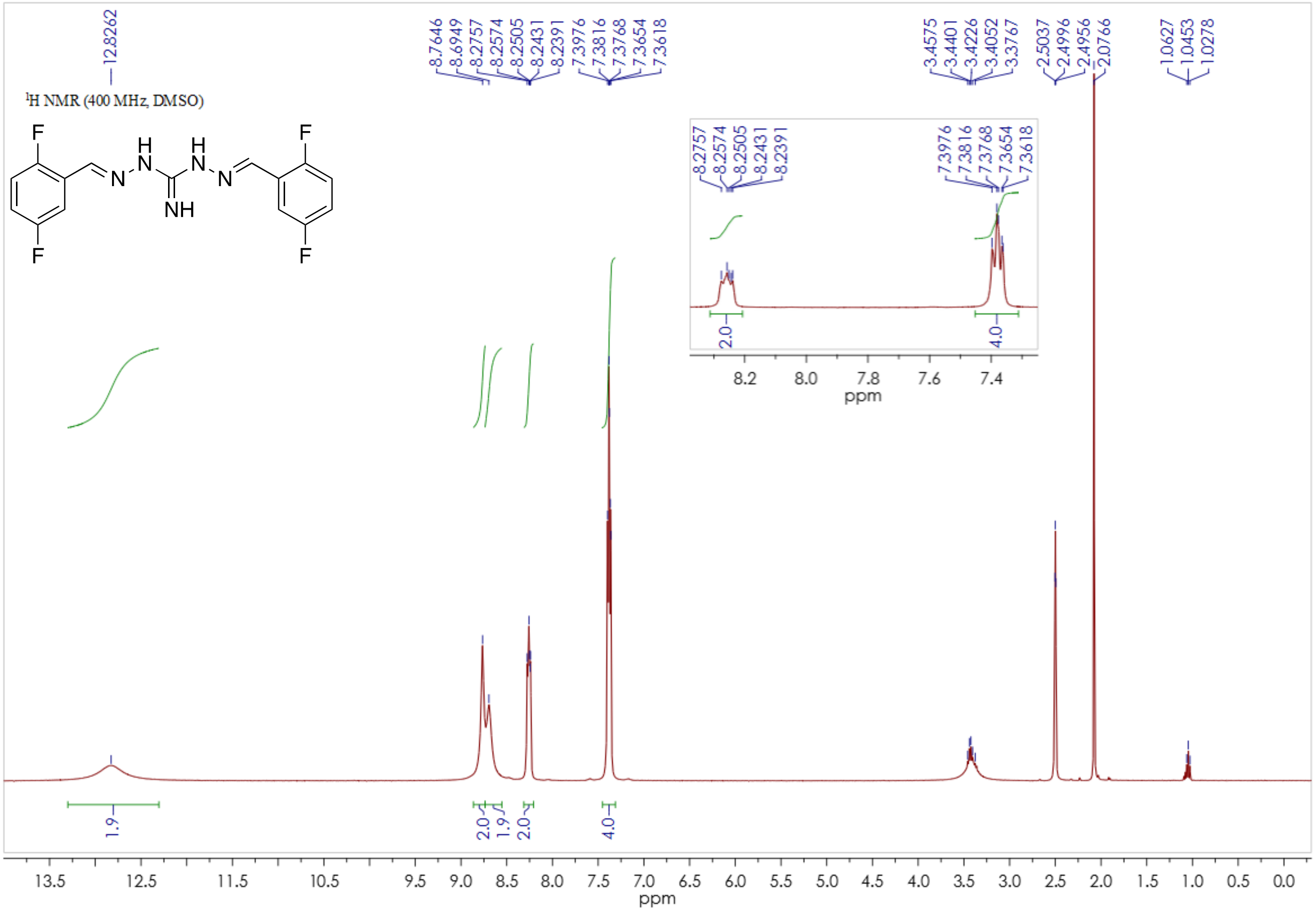

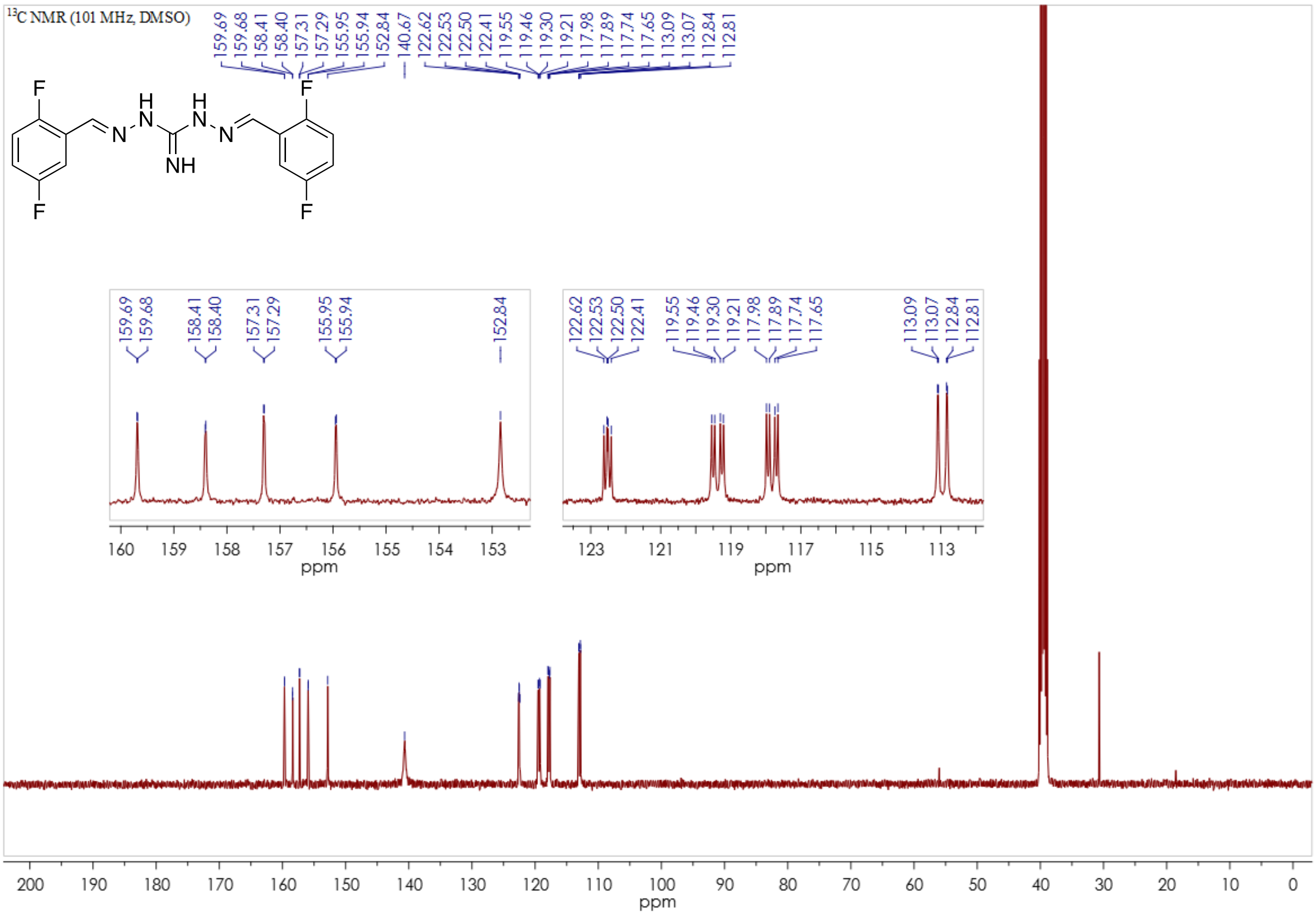

2,2'-Bis[(3,4-difluorophenyl)methylene]carbonimidic dihydrazide hydrochloride (**38**)

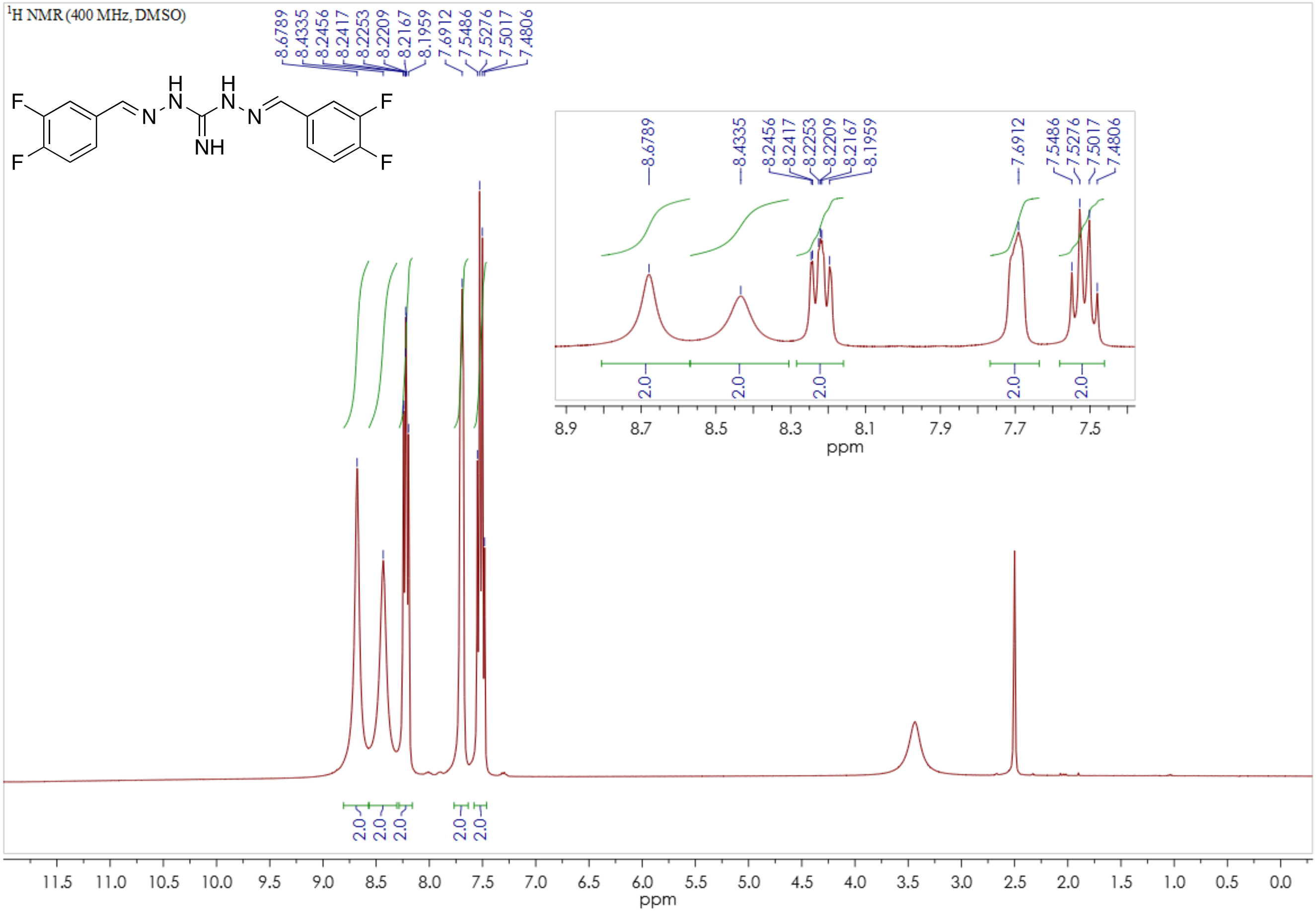

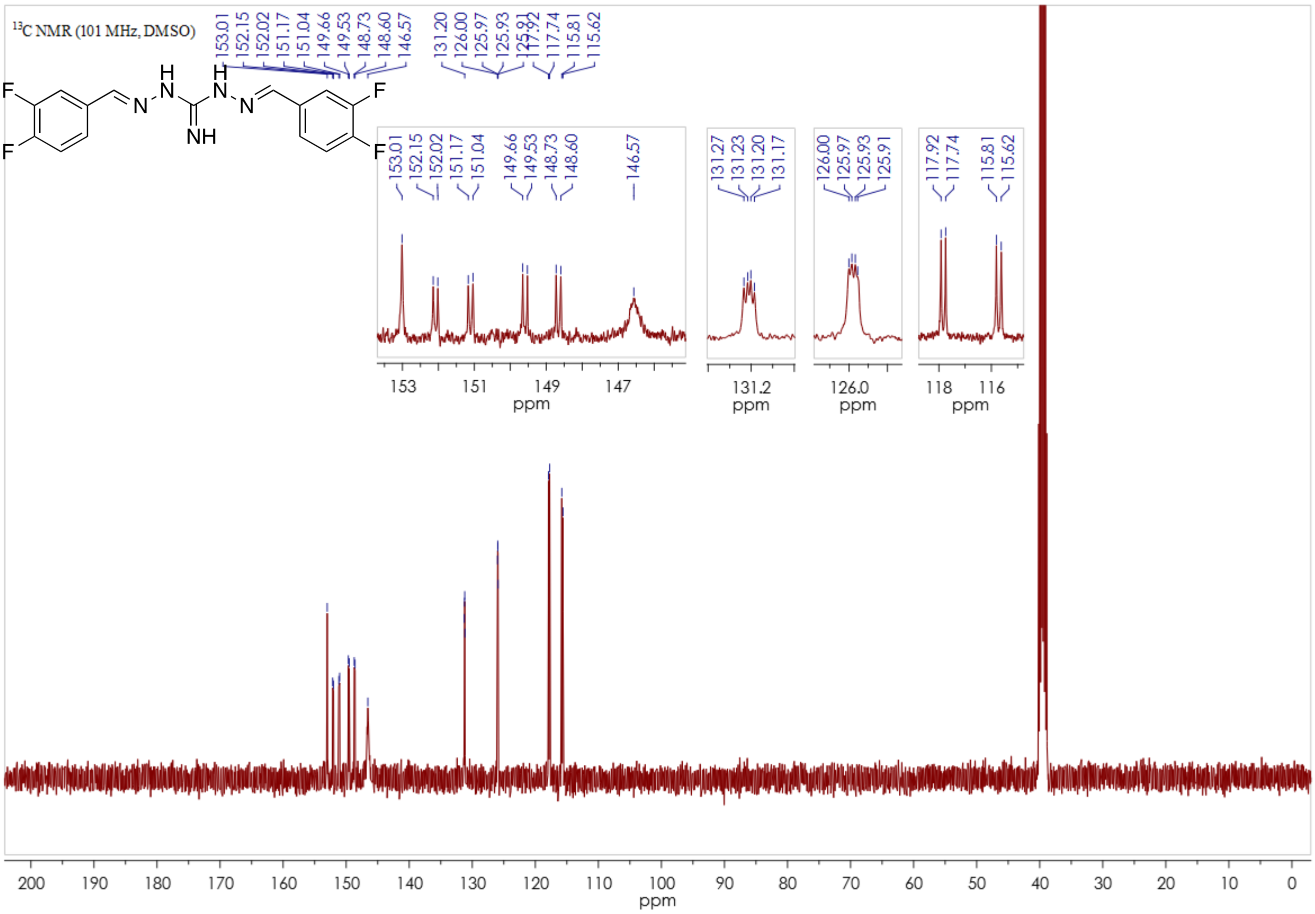

2,2'-Bis[(2,3,4,5,6-pentafluorophenyl)methylene]carbonimidic dihydrazide hydrochloride (**39**)

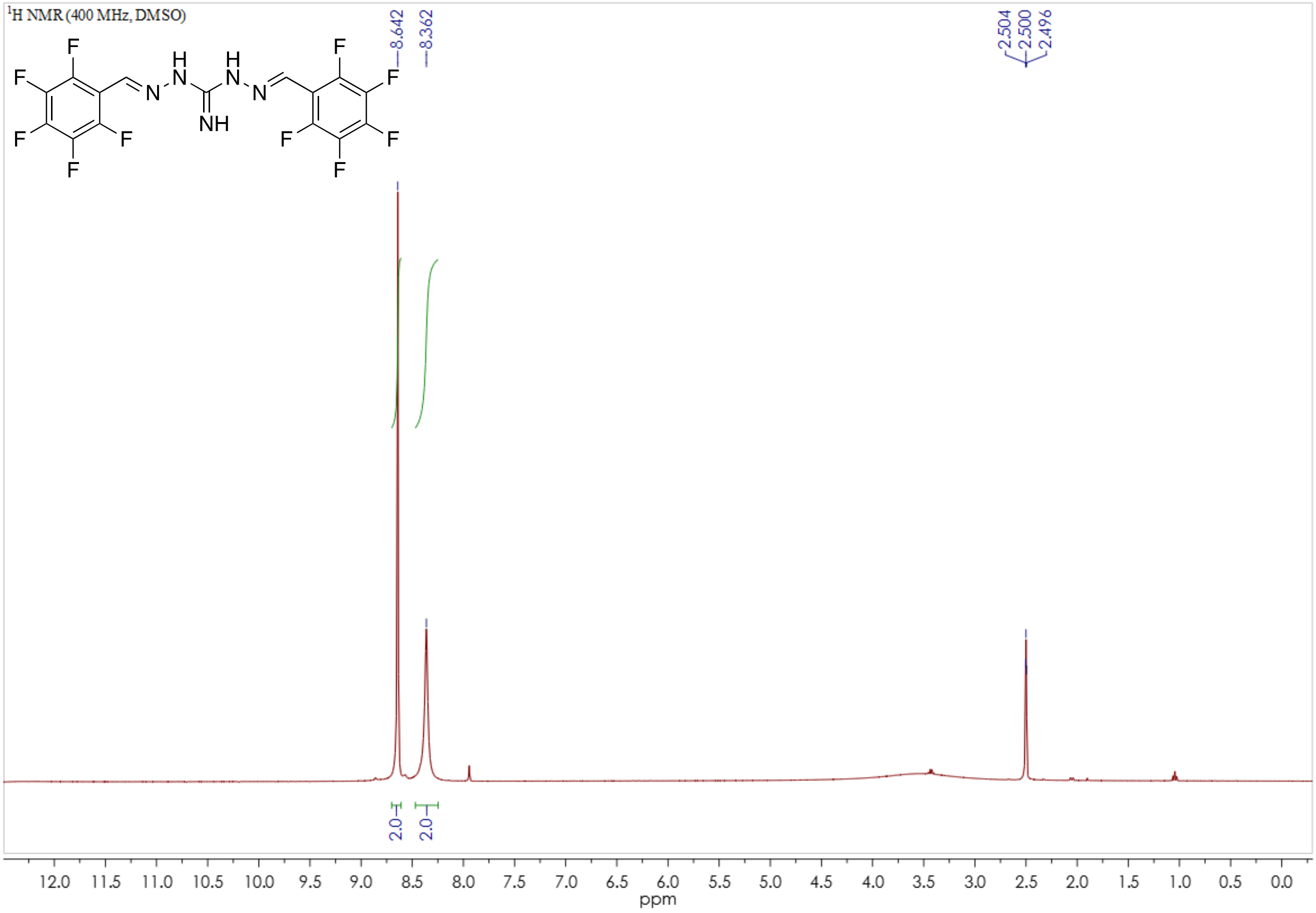

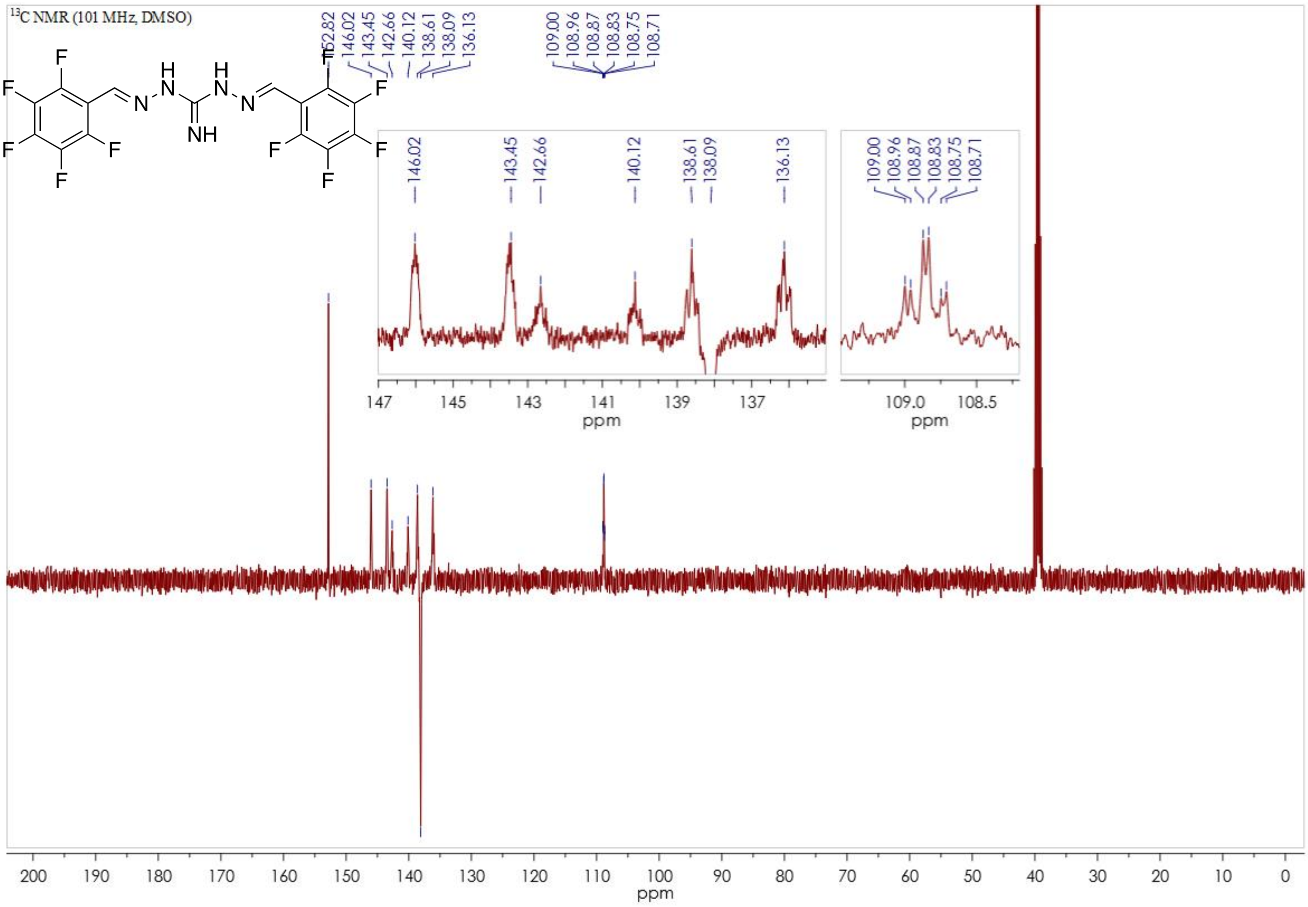

2,2'-Bis[(2-acetylamino-4-chlorophenyl)methylene]carbonimidic dihydrazide hydrochloride (**40**)

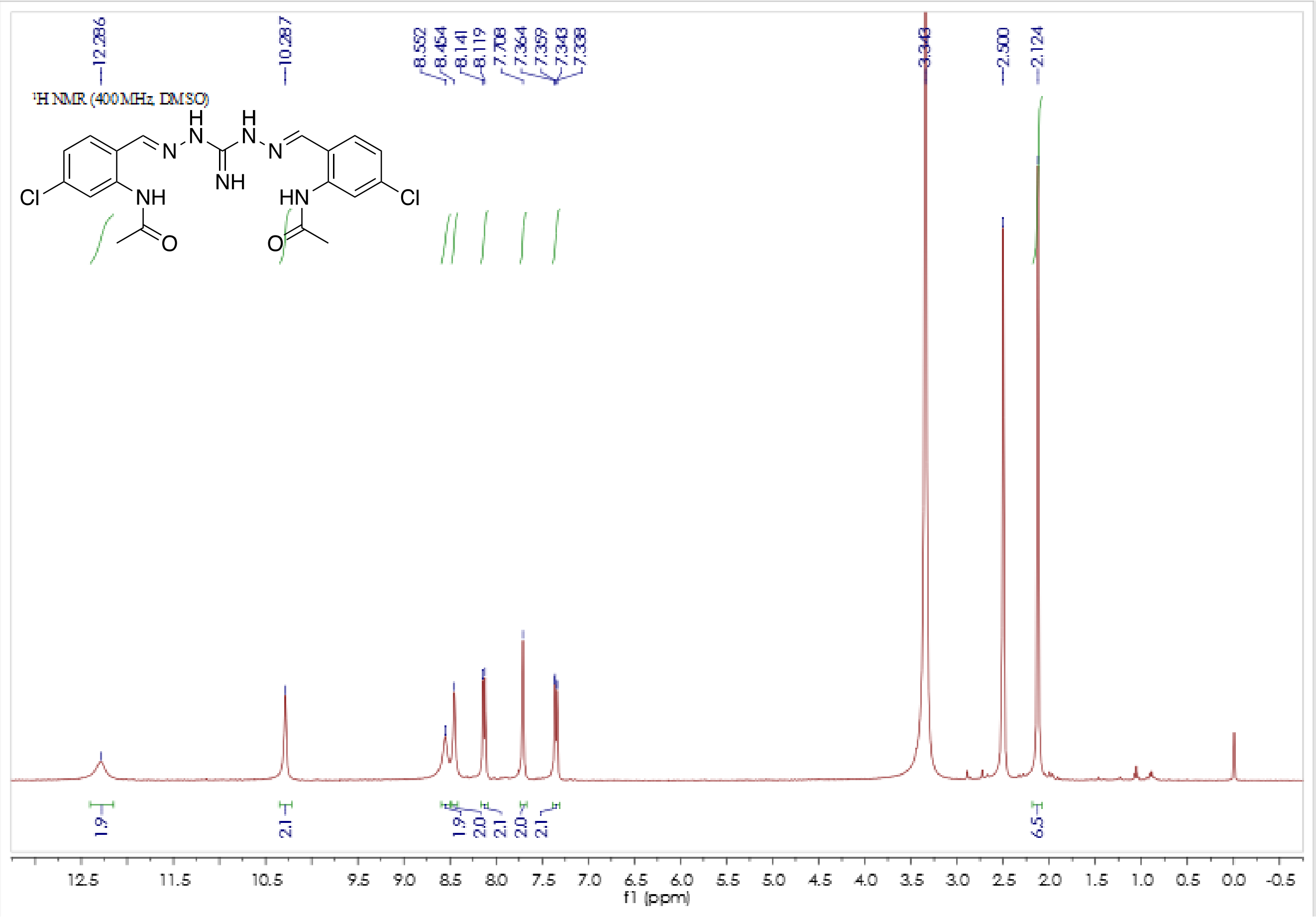

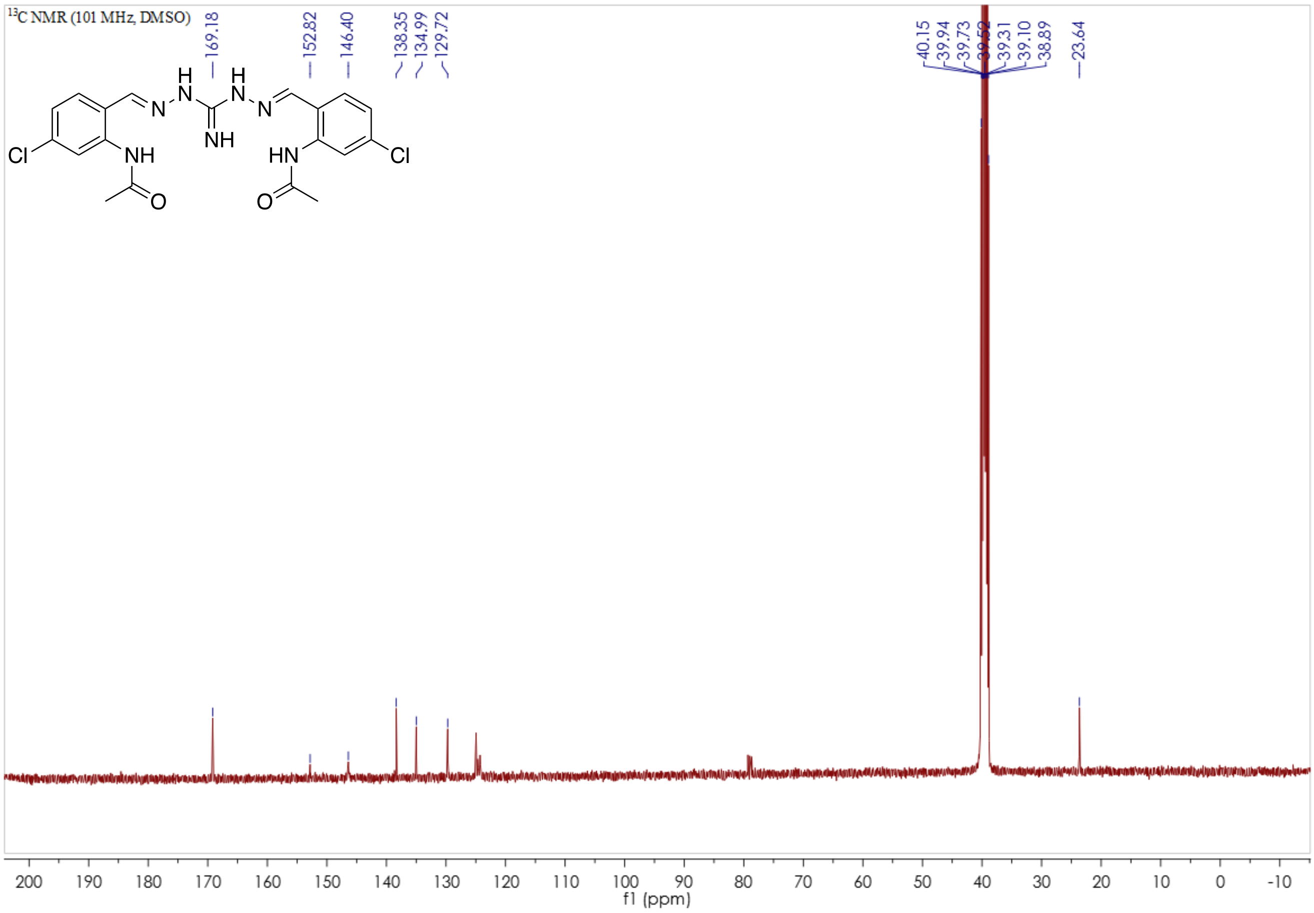

2,2'-Bis[(2-bromo-4,5-dimethoxyphenyl)methylene]carbonimidic dihydrazide hydrochloride (**41**)

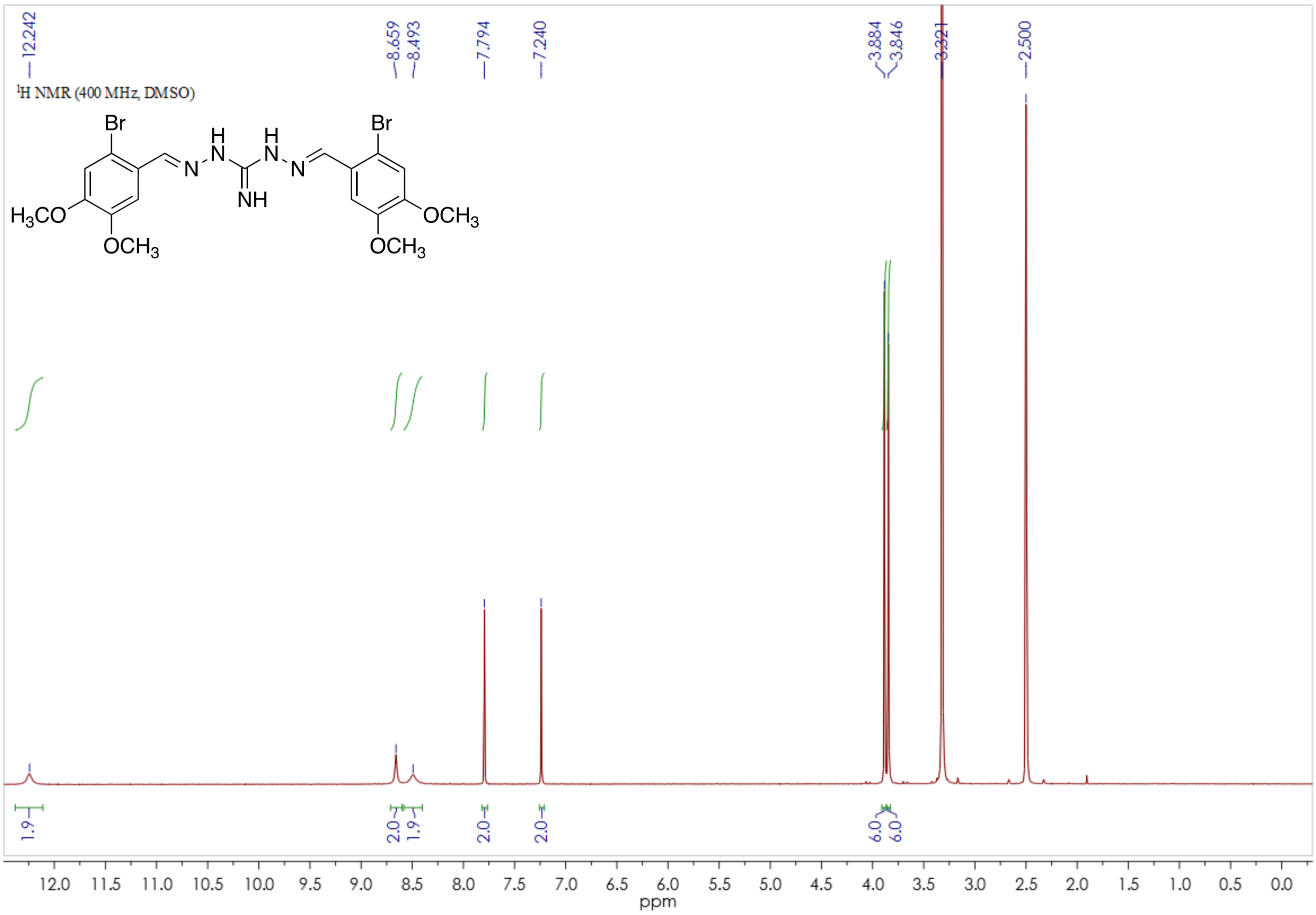

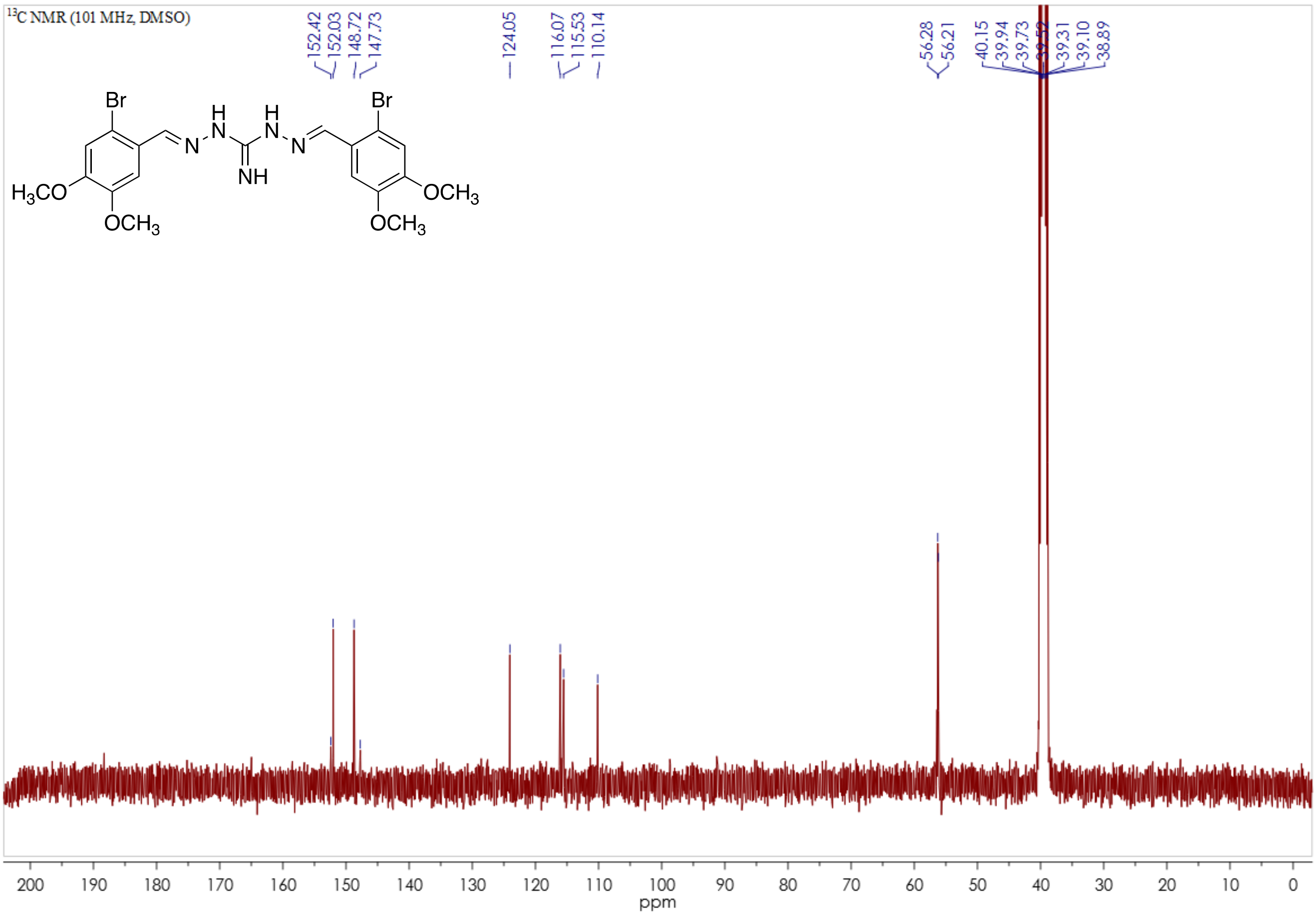

2,2'-Bis[(3-bromo-4,5-dimethoxyphenyl)methylene]carbonimidic dihydrazide hydrochloride (**42**)

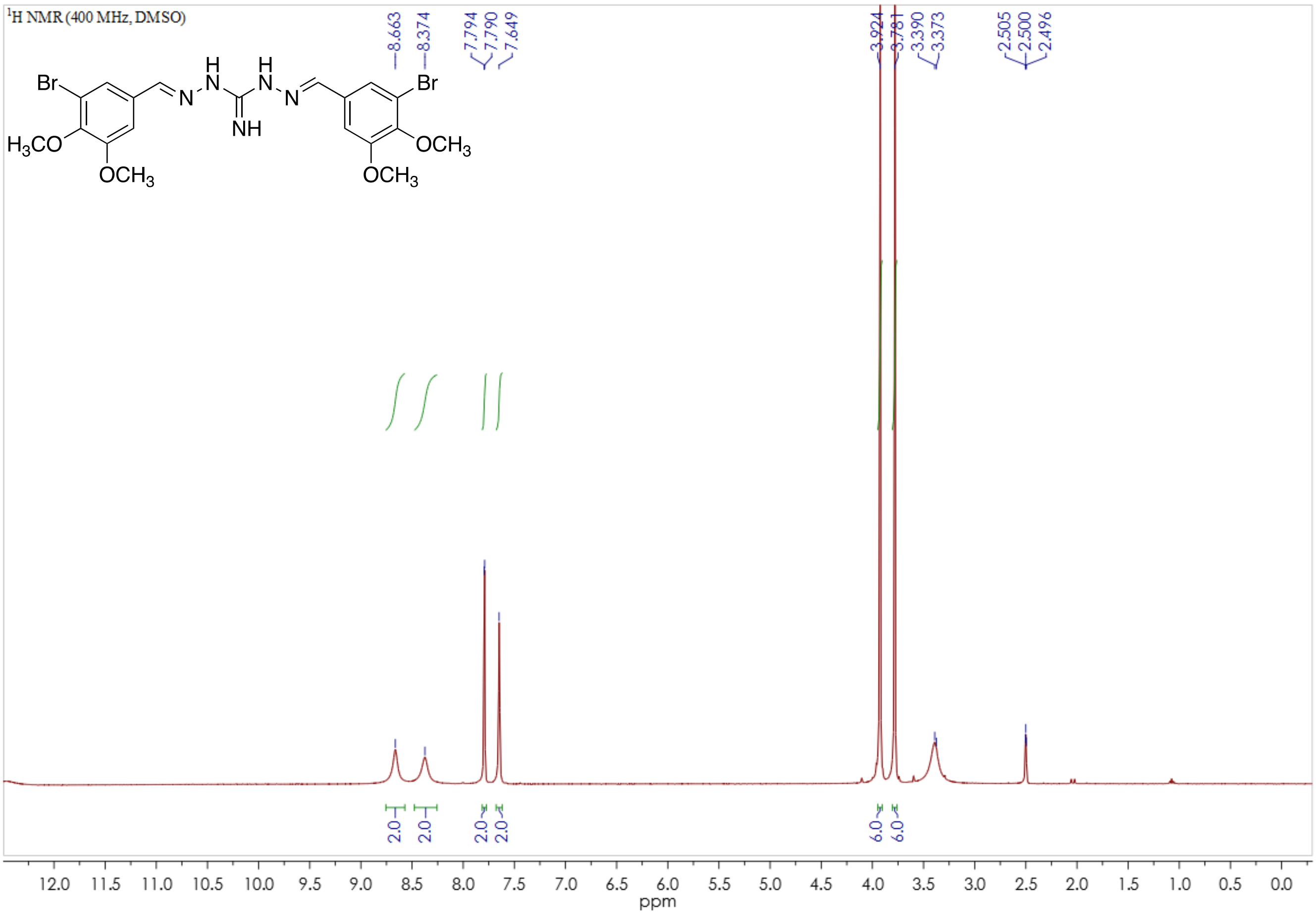

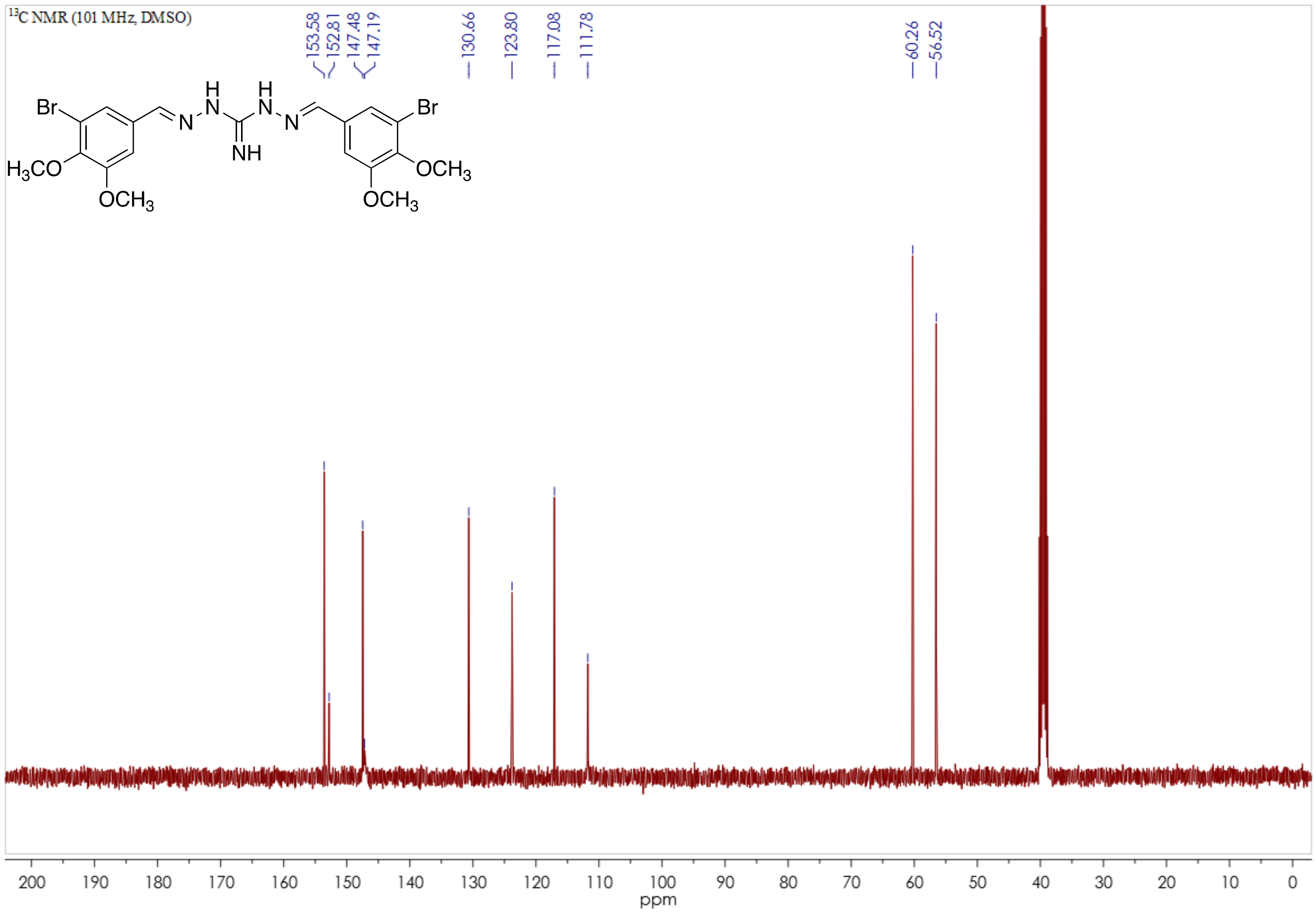

2,2'-Bis{[4-(dimethylamino)-2-hydroxyphenyl]methylene}carbonimidic dihydrazide (**43**)

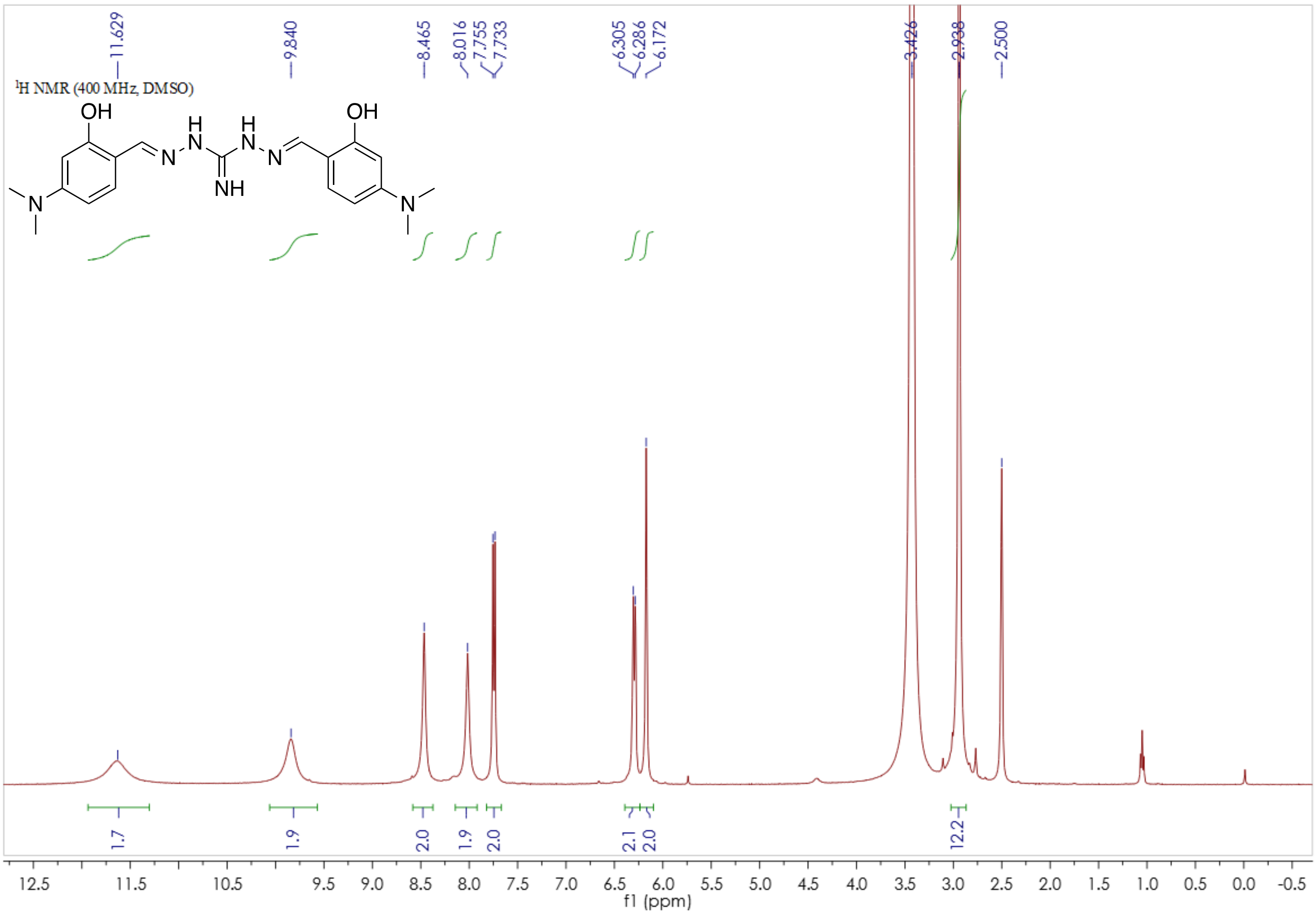

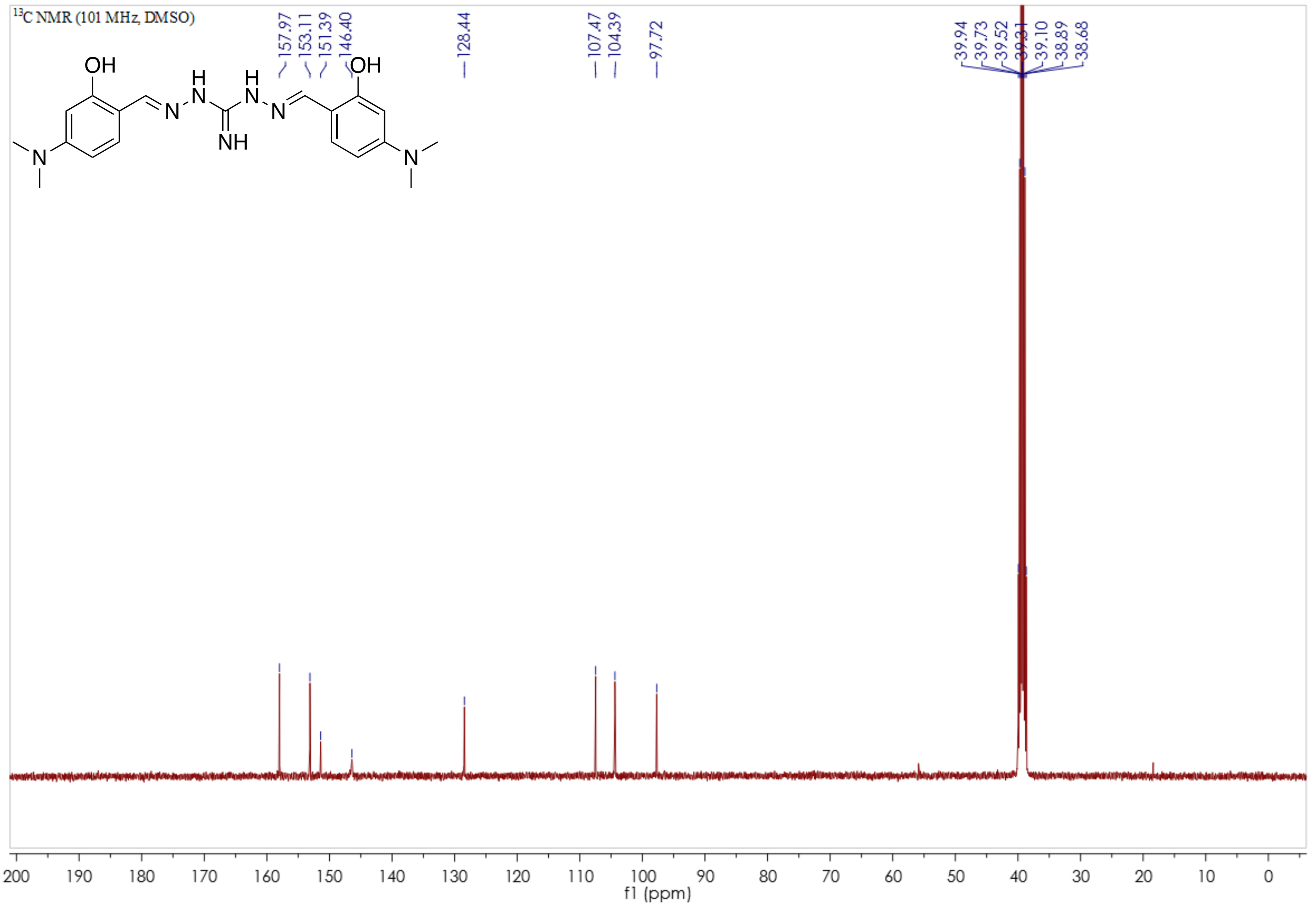

2,2'-Bis[(3,4-dimethoxyphenyl)methylene]carbonimidic dihydrazide hydrochloride (**44**)

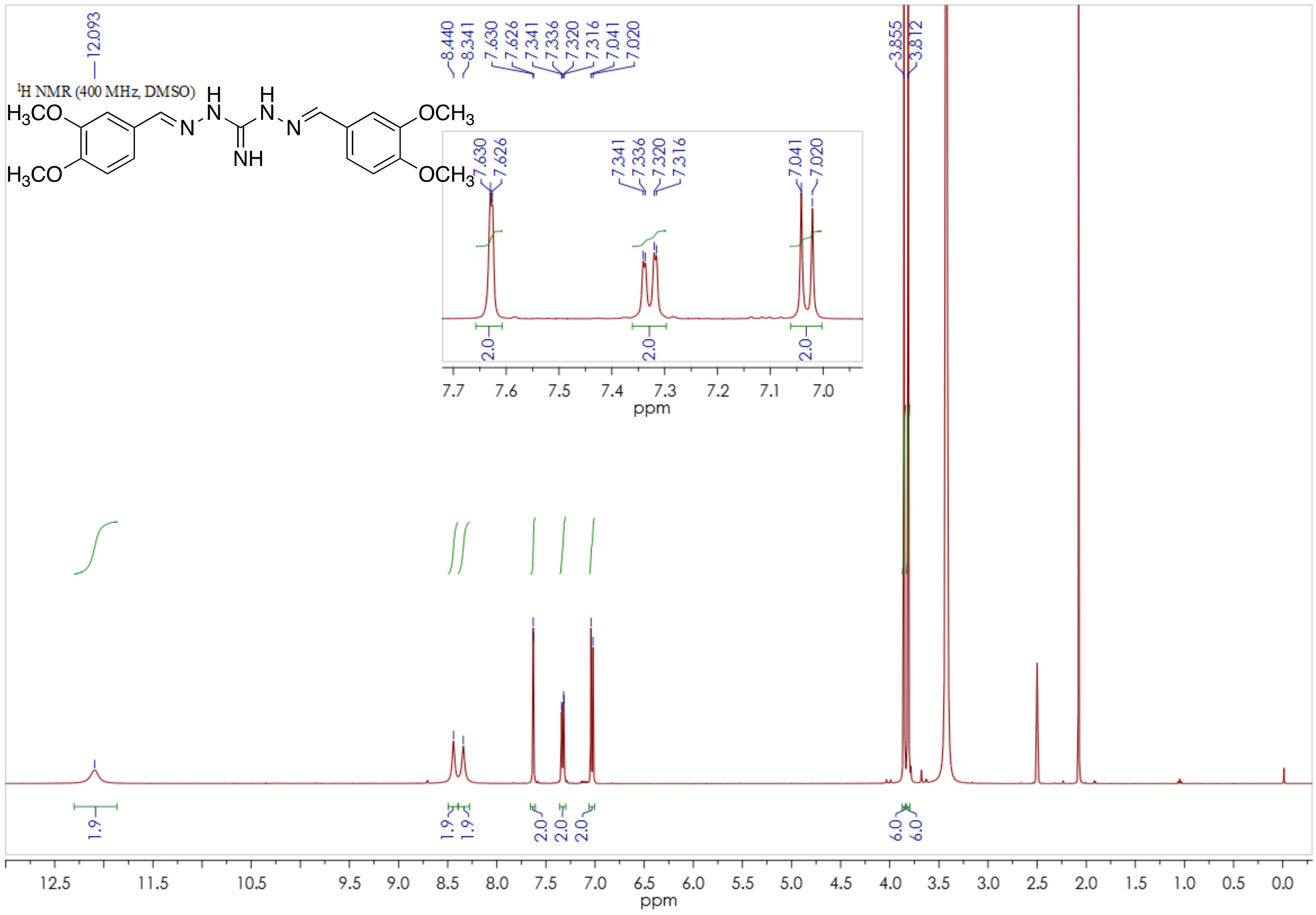

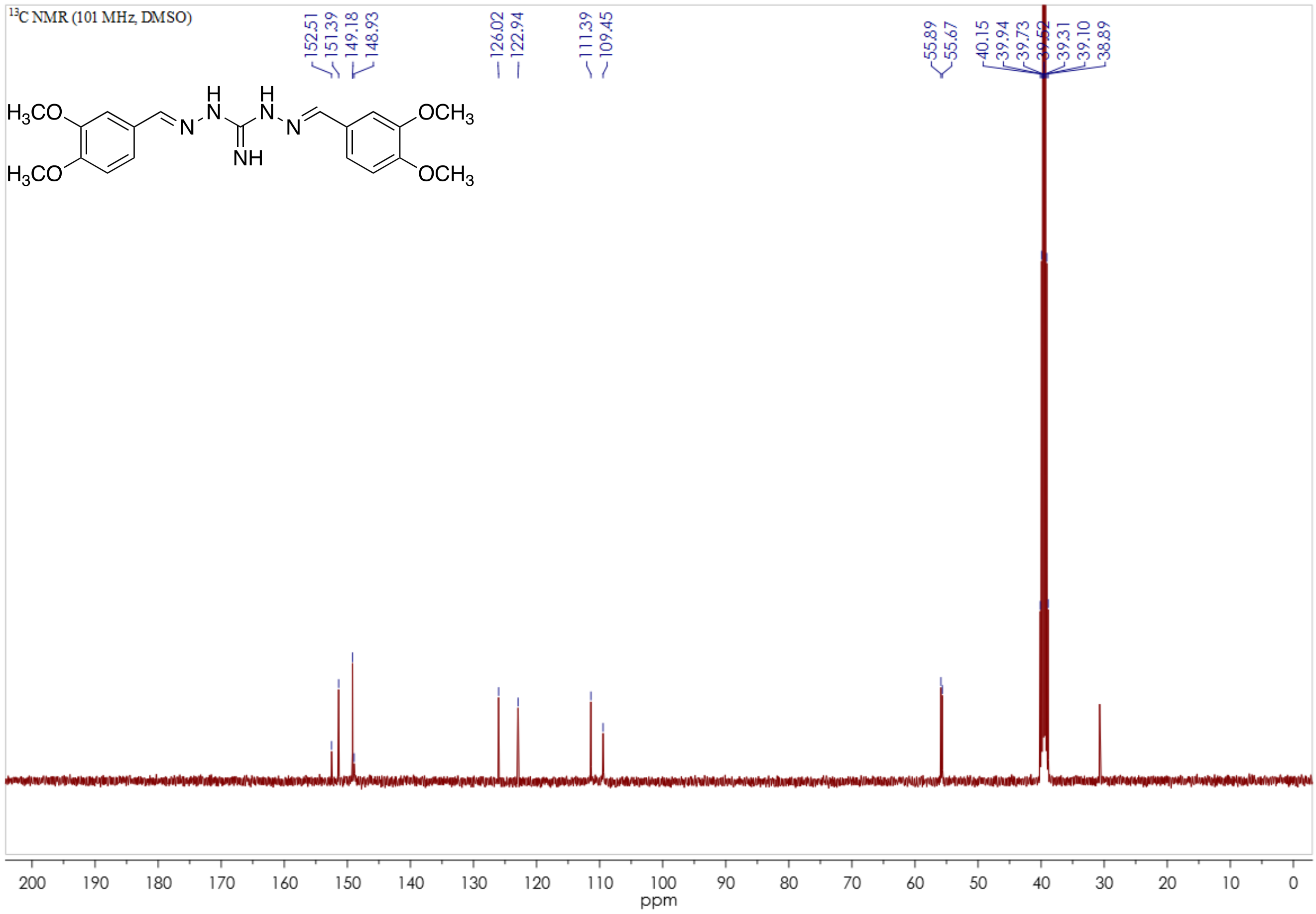

2,2'-Bis[(4-hydroxy-3-nitrophenyl)methylene]carbonimidic dihydrazide hydrochloride (**45**)

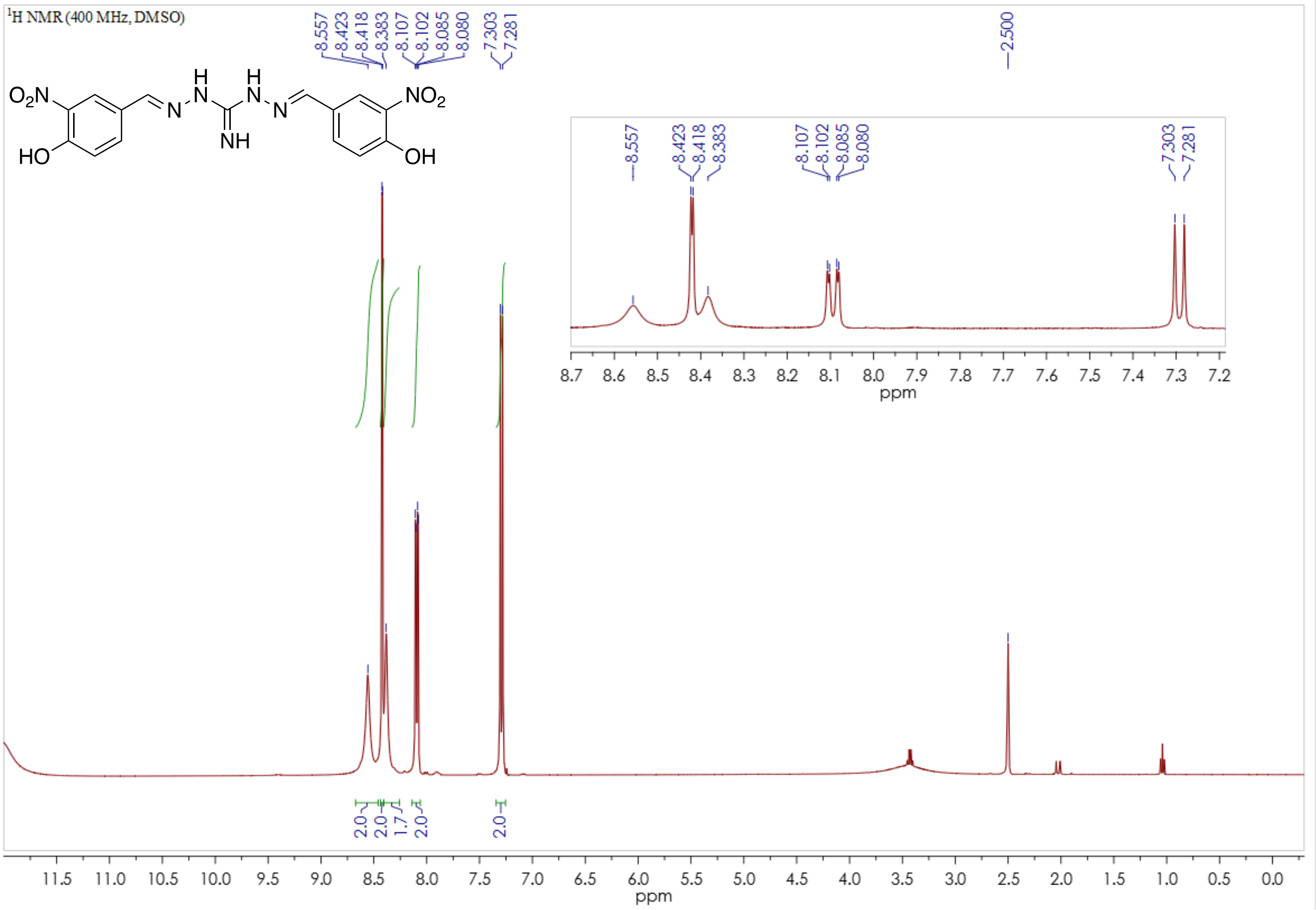

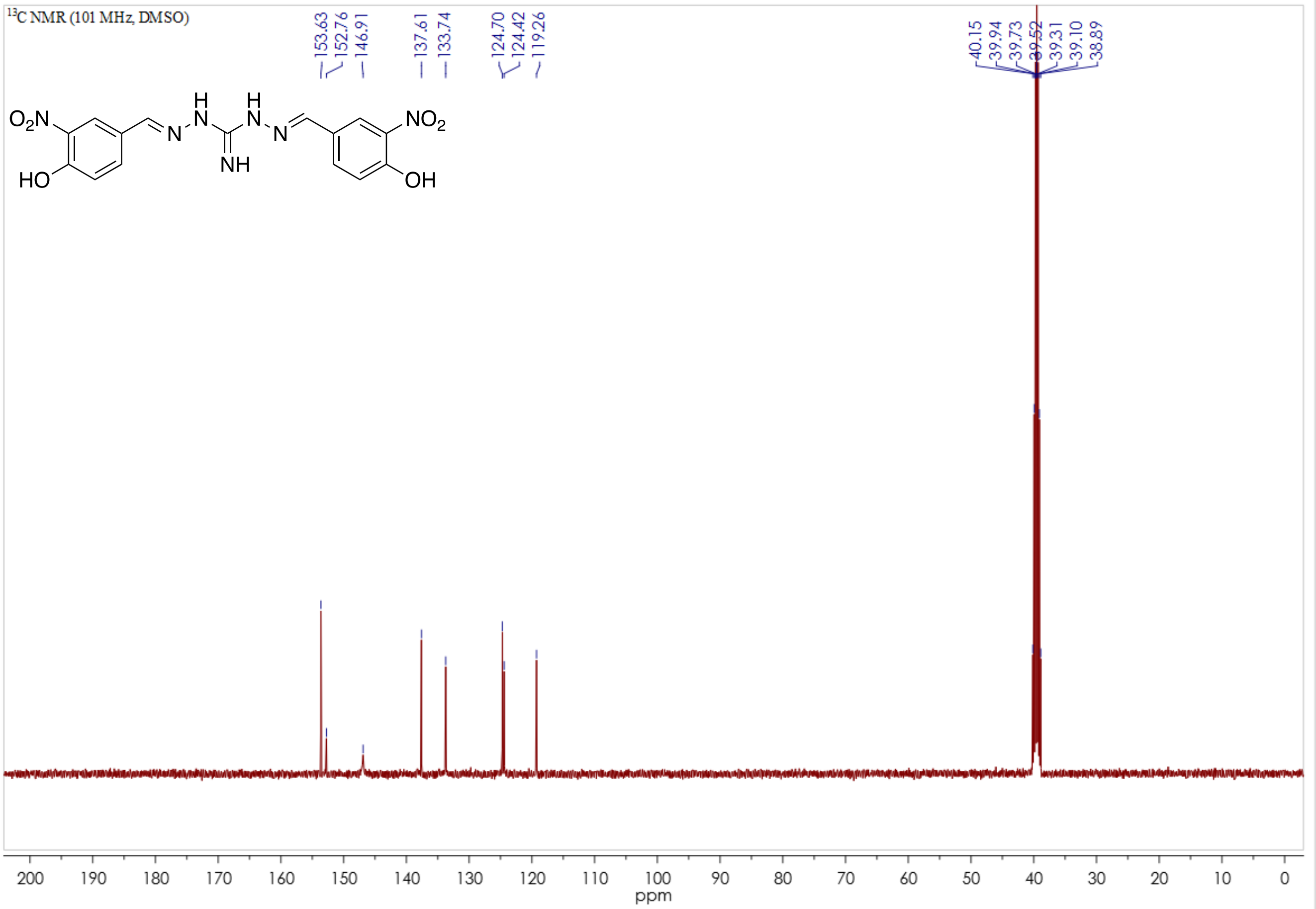

2,2'-Bis[(3-hydroxy-4-methoxyphenyl)methylene]carbonimidic dihydrazide hydrochloride (**46**)

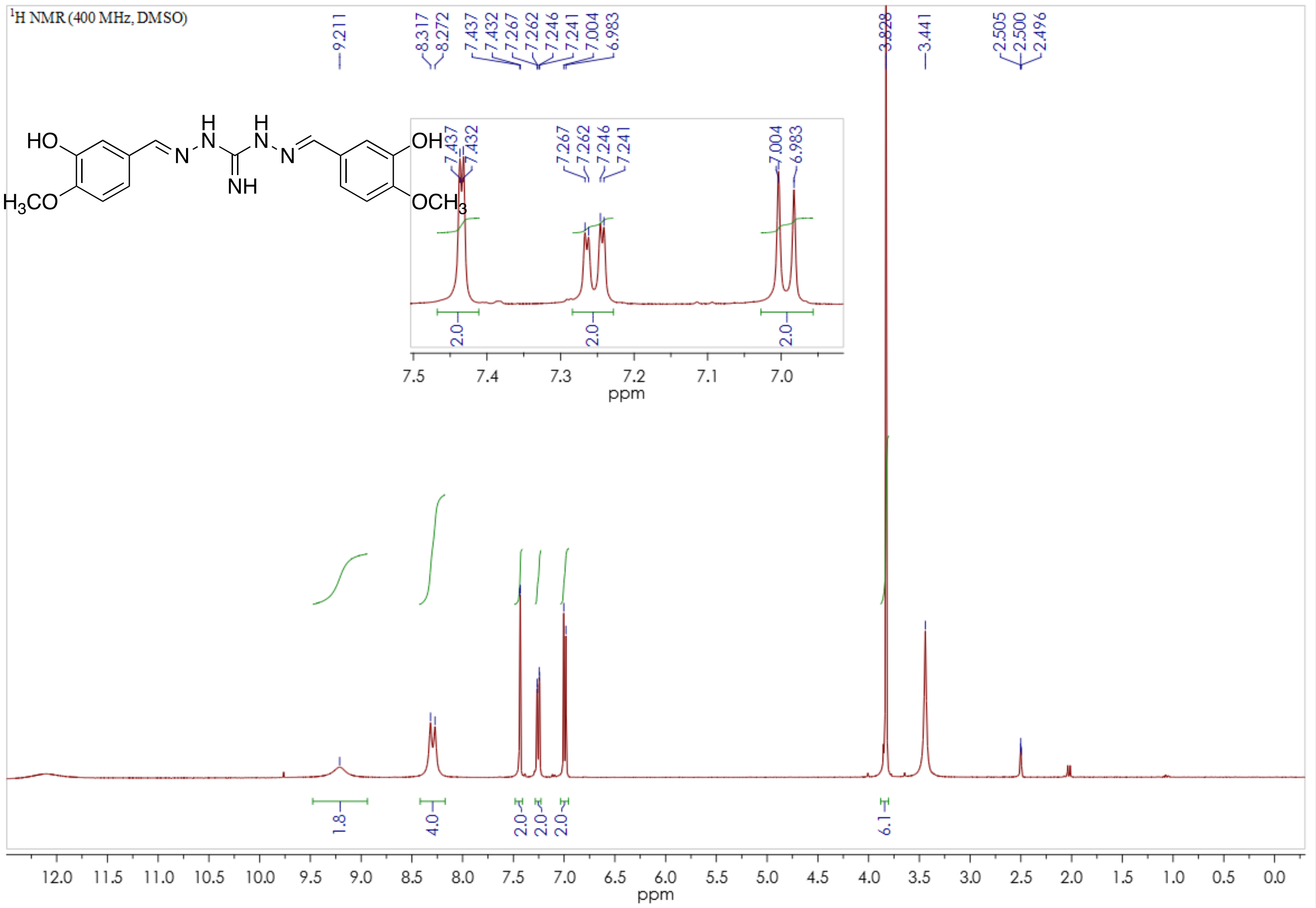

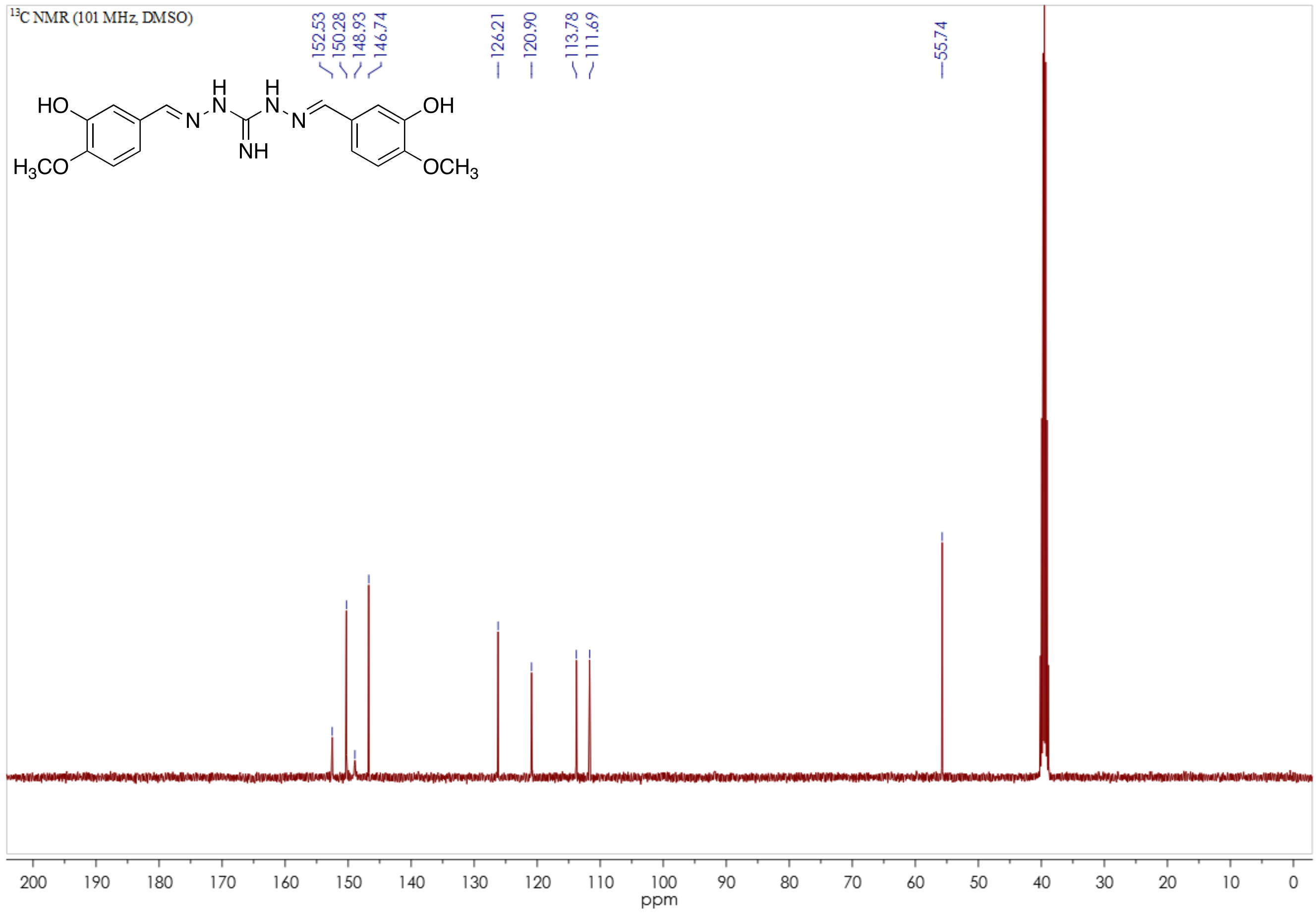

2,2'-Bis[(4-hydroxy-3-methoxyphenyl)methylene]carbonimidic dihydrazide hydrochloride (**47**)

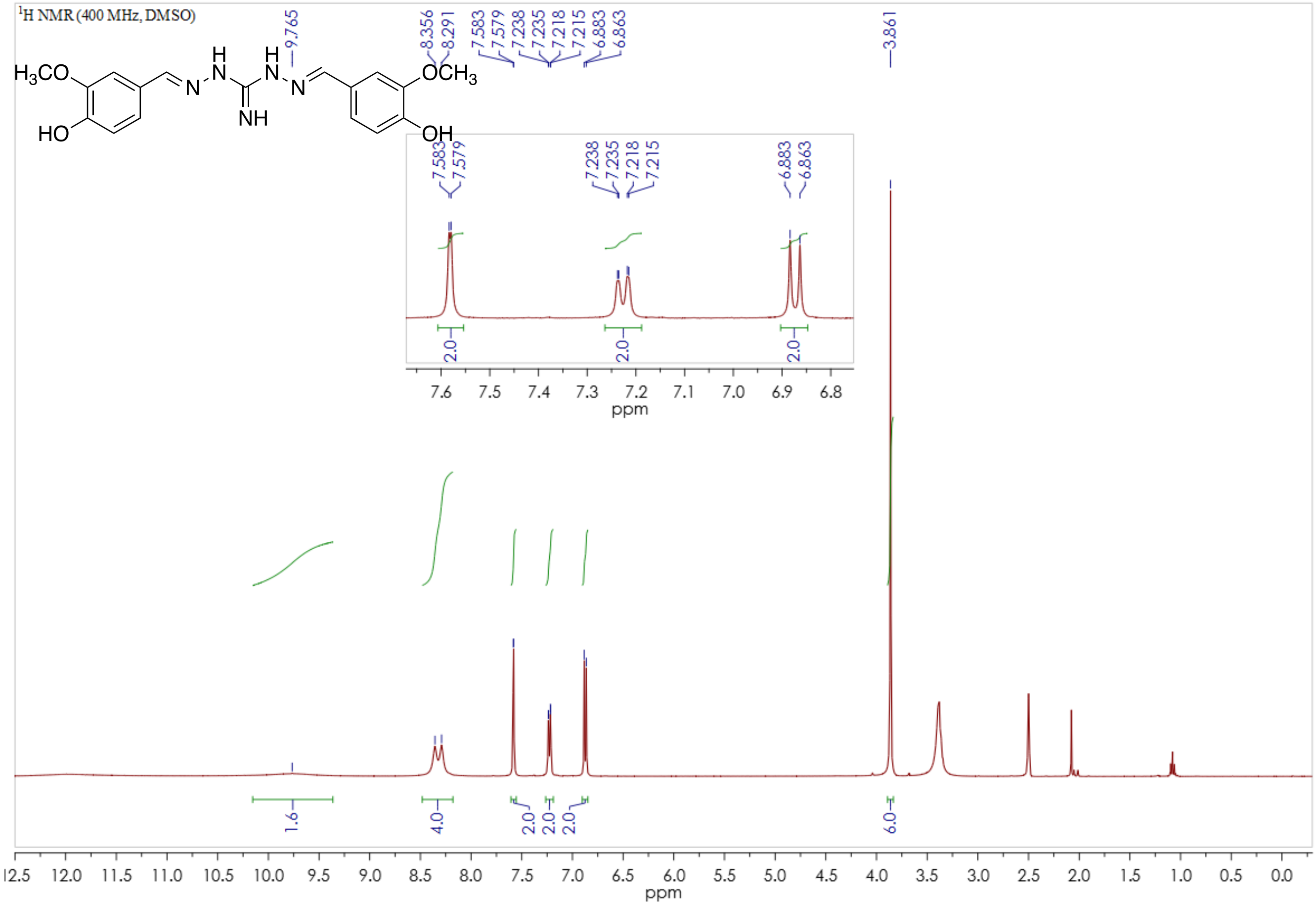

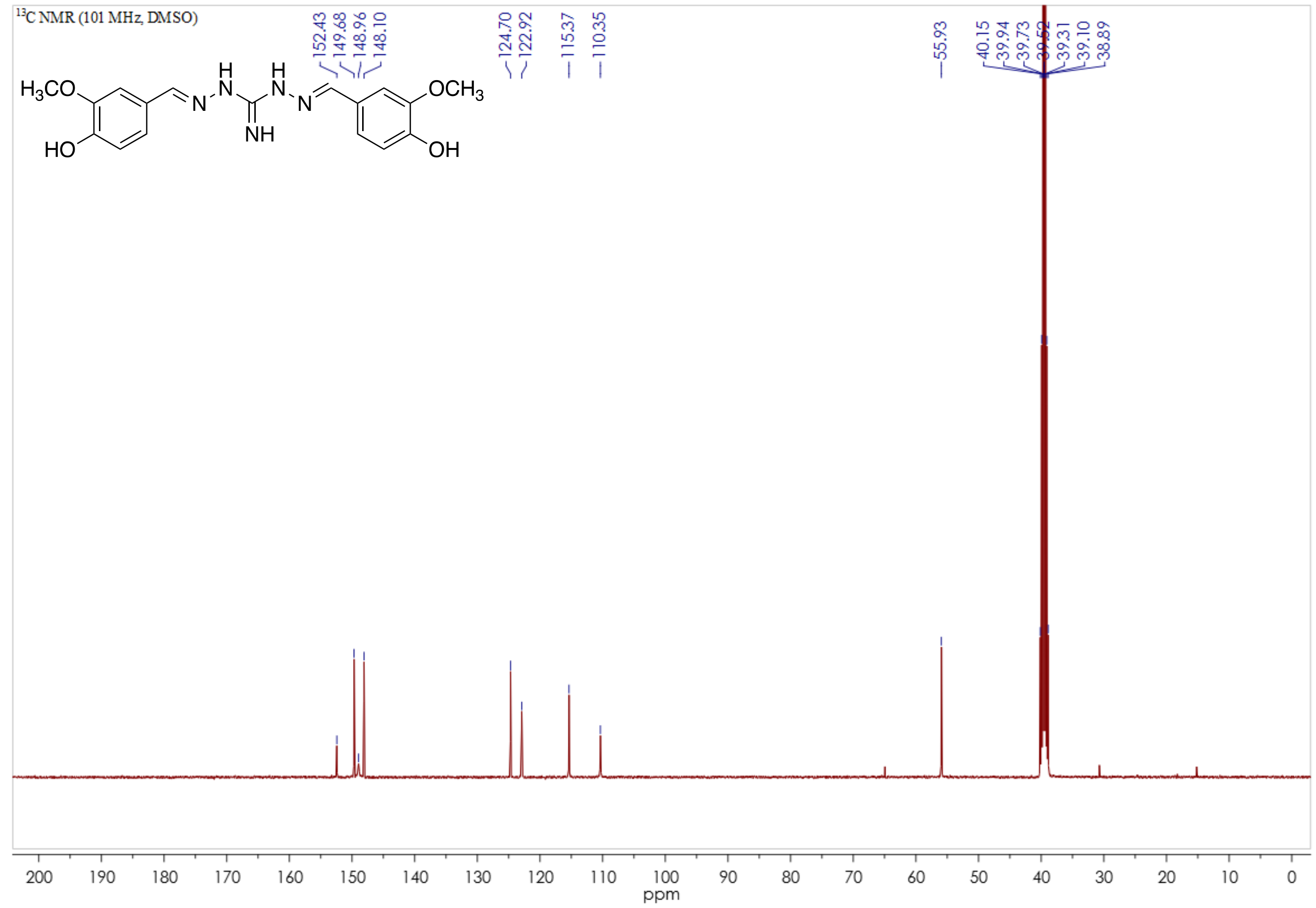

Biology Materials and Methods:

Chemicals

Metronidazole was sourced from sigma.

Chemicals used for the culture of *Giardia in vitro* were sourced as follows: glucose and L-cysteine (ACROS organics, Thermo Fisher Scientific, Scoresby, Vic), Ammonium Iron (III) citrate, ascorbic Acid (Sigma-aldrich, Castle Hill, NSW), potassium dihydrogen orthophosphate (UNIVAR, Ingleburn, NSW), bovine bile (Fluka analytical (BD)), di-potassium hydrogen orthophosphate (Fronine laboratory supplies, Riverstone, NSW).

Cell culture

*G. duodenalis (*BAH2c2 strain) was cultivated according to the method of Clark and Diamond in Keister's modified TYI-S-33 medium, supplemented with heat inactivated foetal bovine serum (Hyclone™, Thermo Scientific, Scoresby, Vic) at 37°C in plastic 9 mL screw-capped test tubes (nunc). Subcultures were performed once a confluent monolayer was observed, approximately 2 – 3 times per week (Clark and Diamond, 2002).

CaCo-2 cells, a human cell line derived from a primary colonic tumour, were sourced from Cellbank Australia and were maintained in MEM supplemented with 2 mM glutamine, 1% non-essential amino acids and 10% FCS (Thermo Scientific). Cells were passaged when confluent, approximately 1-2 times per week. Vero cells were also obtained from Cellbank Australia and maintained as above.

***In vitro* drug efficacy assays**

Resazurin Reduction Assay

The compounds were initially screened at 25 µM and any compound with > 50% inhibitory activity was further tested to identify the IC_{50} and minimum lethal concentration (MLC). For all assays the media of confluent cultures was replaced with fresh media and the cultures were placed on ice for 40 minutes to detach trophozoites. Trophozoites were enumerated using a haemocytometer and 50 000 trophozoites were added to each test well of a 96 well plate. Either single concentrations (final concentration 25 µM) for the screening assay or tripling dilutions of the test compounds (highest concentration 25 µM) for IC_{50} and MLC determinations in DMSO were added to wells. The assays were repeated in triplicate with metronidazole and a DMSO (vehicle only) used as controls. Plates were incubated under anaerobic conditions (anaerogen sachet, Thermofisher scientific) for 5 or 24 hours at 37°C following which the viability of treated cells was determined using the resazurin reduction assay (Benere et al., 2007). The media was removed and replaced with an equal volume of warm PBS. Resazurin (Sigma) was then added at 10% of the total volume of the wells. Plates were further incubated for colour development and absorbance read at 570 nm and 630 nm. Resazurin reduction was calculated according to the following formula:

$$((E_{oxi}630 \times A_{570}) - (E_{oxi}570 \times A_{630}))/((E_{red}570 \times C_{630})-(E_{red} \times C_{570})) \times 100$$

Where: $E_{oxi}630$ = 34798, $E_{oxi}570$ = 80586, A_{570} = absorbance at 570nm, A_{630} = absorbance at 630 nm, $E_{red}570$ = 155677, $E_{red}630$ = 5494, C_{630} = absorbance of negative control well at 630 nm and C_{570} = absorbance of negative control well at 570 nm.

Antibacterial activity

The antibacterial activity was confirmed using *S. aureus* ATCC 29213 and *E. coli* ATCC 25922 in Leuria Bertanini broth for a random selection of compounds (Thermofisher Scientific). Compounds were diluted in DMSO to 100X the final desired concentration and 2 µl added to 178 µl of broth. Bacterial cells were suspended in 0.9 % saline to a concentration equivalent to a 0.5 McFarland standard, diluted 1:20 in saline and 20 µl of diluted suspension added to the assay plate to give a final cell concentration of ~ 5 X 10^5 CFU/ml. Cells were incubated for 18-24 hrs before interpretation of results. Antibacterial activity of remaining analogues was sourced from the previous publication describing this work (Abraham et al*, J Med Chem* **2016**, *59*, 2126-2138).

***In vitro* cytotoxicity**

CaCo-2 or Vero cells, maintained as outlined above, were collected via trypsinisation and enumerated using trypan blue and a haemocytometer. Select analogues were prepared in DMSO so that the final concentration in the assay was 25 µM. 1 X 10^5 cells/ml were added to each assay with the appropriate drug dilution. Assays were completed in 96 well plates and incubated for 24 hours at 37°C, 5% CO_2 in a humidified incubator. After incubation cells were

washed with warm PBS before the addition of 10% resazurin in PBS. Assays were incubated to allow colour development and absorbance read at 630 and 570nm. Activity of the analogues against mammalian cells was calculated according to the formula stated above.

Statistical analysis

The results for the *in vitro* drug efficacy studies were analysed using GraphPad Prism 6 software. For the resazurin reduction assay the mean and standard error of the mean was determined with each assay completed in triplicate. The IC_{50} was calculated using the log (inhibitor) vs. normalised response – variable slope function in Graphpad Prism.

***In vivo* toxicity in mice**

Suckling mice (arc:arc(s) – swiss origin mice) were obtained from the animal resource center at Murdoch (WA) and were 3-4 weeks old at the time of the trial. Mice were given 100 mg/kg of robenidine, **41** or metronidazole once per day in a volume of 0.1 ml for 3 days. Control groups were given an equivalent volume of saline or the formulation used to deliver the compounds. Mice were observed for behavioral signs of toxicity and gross pathology was examined at the end of the exposure period. Animal ethics granted through Murdoch University (permit number: R2855/16).
